# Transcriptome profile analysis reflects rat liver and kidney damage following chronic ultra-low dose Roundup exposure

**DOI:** 10.1186/s12940-015-0056-1

**Published:** 2015-08-25

**Authors:** Robin Mesnage, Matthew Arno, Manuela Costanzo, Manuela Malatesta, Gilles-Eric Séralini, Michael N. Antoniou

**Affiliations:** Gene Expression and Therapy Group, Faculty of Life Sciences & Medicine, Department of Medical and Molecular Genetics, King’s College London, 8th Floor Tower Wing, Guy’s Hospital, Great Maze Pond, London, SE1 9RT UK; Genomics Centre, King’s College London, Waterloo Campus, 150 Stamford Street, London, SE1 9NH UK; Department of Neurological and Movement Sciences, University of Verona, Verona, 37134 Italy; Institute of Biology, EA 2608 and Risk Pole, MRSH-CNRS, Esplanade de la Paix, University of Caen, Caen, 14032 Cedex France

**Keywords:** Pesticides, Glyphosate, Transcriptome, Chronic toxicity, Liver, Kidney

## Abstract

**Background:**

Glyphosate-based herbicides (GBH) are the major pesticides used worldwide. Converging evidence suggests that GBH, such as Roundup, pose a particular health risk to liver and kidneys although low environmentally relevant doses have not been examined. To address this issue, a 2-year study in rats administering 0.1 ppb Roundup (50 ng/L glyphosate equivalent) via drinking water (giving a daily intake of 4 ng/kg bw/day of glyphosate) was conducted. A marked increased incidence of anatomorphological and blood/urine biochemical changes was indicative of liver and kidney structure and functional pathology. In order to confirm these findings we have conducted a transcriptome microarray analysis of the liver and kidneys from these same animals.

**Results:**

The expression of 4224 and 4447 transcript clusters (a group of probes corresponding to a known or putative gene) were found to be altered respectively in liver and kidney (*p* < 0.01, *q* < 0.08). Changes in gene expression varied from −3.5 to 3.7 fold in liver and from −4.3 to 5.3 in kidneys. Among the 1319 transcript clusters whose expression was altered in both tissues, ontological enrichment in 3 functional categories among 868 genes were found. First, genes involved in mRNA splicing and small nucleolar RNA were mostly upregulated, suggesting disruption of normal spliceosome activity. Electron microscopic analysis of hepatocytes confirmed nucleolar structural disruption. Second, genes controlling chromatin structure (especially histone-lysine N-methyltransferases) were mostly upregulated. Third, genes related to respiratory chain complex I and the tricarboxylic acid cycle were mostly downregulated. Pathway analysis suggests a modulation of the mTOR and phosphatidylinositol signalling pathways. Gene disturbances associated with the chronic administration of ultra-low dose Roundup reflect a liver and kidney lipotoxic condition and increased cellular growth that may be linked with regeneration in response to toxic effects causing damage to tissues. Observed alterations in gene expression were consistent with fibrosis, necrosis, phospholipidosis, mitochondrial membrane dysfunction and ischemia, which correlate with and thus confirm observations of pathology made at an anatomical, histological and biochemical level.

**Conclusion:**

Our results suggest that chronic exposure to a GBH in an established laboratory animal toxicity model system at an ultra-low, environmental dose can result in liver and kidney damage with potential significant health implications for animal and human populations.

**Electronic supplementary material:**

The online version of this article (doi:10.1186/s12940-015-0056-1) contains supplementary material, which is available to authorized users.

## Background

Glyphosate-based herbicides (GBH), such as Roundup, are the major pesticides used worldwide. GBH are currently applied on at least 24 % of the total global cropland (Benbrook C, personal communication), and also used extensively in domestic and urban environments. Residues of GBH are routinely detected in foodstuffs [1, 2] and also drinking water contaminated via rain, surface runoff and leaching into groundwater, thereby increasing possible routes of exposure [3]. Epidemiological data on the human body burden of GBH residues is very limited but evidence suggests that glyphosate and its metabolites are wide-spread [4].

Glyphosate’s primary perceived mode of herbicidal action is to inhibit 5-enolpyruvylshikimate-3-phosphate synthase (EPSPS) of the shikimate aromatic amino acid biosynthesis pathway present in plants and some bacteria. Since this pathway is absent in vertebrates, it is generally assumed that glyphosate poses minimal health risks to mammals, including humans [5]. However, converging evidence suggests that GBH residues pose a particular risk to kidney and liver function. Hepatic effects of glyphosate were first observed in the 1980s, including its ability to disrupt liver mitochondrial oxidative phosphorylation [6]. As glyphosate can act as a protonophore increasing mitochondrial membrane permeability to protons and Ca^2+^ [7], it can trigger the production of reactive oxygen species resulting in observed oxidative stress [8]. Elevation in oxidative stress markers is detected in rat liver and kidney after subchronic exposure to GBH at the United States permitted glyphosate concentration of 700 μg/L in drinking water [9]. Hepatic histological changes and alterations of clinical biochemistry are detected in rats consuming 4.87 mg/kg body weight (bw) glyphosate every 2 days over 75 days [10].

Metabolic studies in a variety of laboratory and farm animals show levels of glyphosate and aminomethylphosphonic acid (AMPA, the principal breakdown product of glyphosate) in kidney and liver tissues that are 10- to 100-fold or even greater than the levels found in fat, muscle, and most other tissues [11]. In farm animals, elevated glyphosate urinary levels are correlated with alterations in blood serum parameters indicative of liver and kidney oxidative stress and depletion in nutrient trace element levels [12].

In addition to these cytotoxic effects, studies have suggested that GBH can disrupt several endocrine-signaling systems, including estrogen [13] and retinoic acid [14]. Endocrine disruptive effects may explain reproductive developmental impairment in rats exposed to sub-lethal doses of GBH [15]. Effects on retinoic acid signalling pathways have been proposed to account for the potential teratogenic effects of GBH in mammals [16] and amphibians [14].

Nevertheless, it should be noted that most results from these GBH toxicity studies were obtained at doses far greater than general human population exposure. Doses tested were typically over the glyphosate acceptable daily intake (ADI), which is currently set at 0.3 mg/kg bw/day within the European Union and 1.75 mg/kg bw/day in the USA based on hepatorenal toxicity measurements after chronic exposure in rats, although GBH toxicity was not investigated in life-long experiments.

In order to address this issue, a 2-year study was conducted where rats were administered via drinking water at a concentration of 0.1 ppb Roundup, thus containing not only glyphosate but also adjuvants [17]. The glyphosate equivalent concentration was 0.05 μg/L and corresponds to an admissible concentration within the European Union (0.1 μg/L) and USA (700 μg/L). The results showed that Roundup caused an increased incidence of anatomical signs of pathologies, as well as changes in urine and blood biochemical parameters suggestive of liver and kidney functional insufficiency in both sexes. In an effort to confirm these findings through a more quantitative molecular biological approach and obtain insight into the alterations in gene expression profiles associated with the observed increased signs of kidney and liver anatomorphological pathologies, we conducted a full transcriptomic analysis of these organs from the female cohort of animals. A large number of transcript clusters (>4000) were found to be altered in their level of expression in both the liver and kidneys of the Roundup treated group relative to controls and to a very high statistical significance. The alterations in gene expression profiles are typical of disturbances measured in cases of fibrosis, necrosis, phospholipidosis, mitochondrial membrane dysfunction and ischemia. Therefore our results confirm the ultra-low dose Roundup-induced increased incidence of hepatorenal pathologies suggested by observations at an anatomical, histological and biochemical level.

## Methods

### Experimental design

The tissues analyzed in this study were obtained from animals as previously described [17]. Briefly, the experimental protocol was as follows. Following 20 days of acclimatization, 5 week old Harlan Sprague–Dawley rats were randomly assigned on a weight basis into groups of 10 animals. Animals were fed with the standard diet A04 (Safe, France) including 33 % maize DKC 2675 over 2 years. All feed formulations consisted of a balanced diet, chemically measured as substantially equivalent. All animals were kept in polycarbonate cages (820 cm^2^, Genestil, France). The location of each cage within the experimental room was regularly changed. The litter (Toplit classic, Safe, France) was replaced twice weekly. The animals were maintained at 22 ± 3 °C under controlled humidity (45 to 65 %) and air purity with a 12 h-light/dark cycle, with free access to food and water. All reagents used were of analytical grade. The animal experimental protocol was conducted in accordance with the regulations of the local ethics committee in an animal care unit authorized by the French Ministries of Agriculture and Research (Agreement Number A35-288-1). Animal experiments were performed according to ethical guidelines of animal experimentation (regulation CEE 86/609).

Groups of 10 animals had access to either plain water (control) or to the same water supplemented with 1.1e-8 % of Roundup (0.1 ppb or 50 ng/L glyphosate equivalent dilution). The commercial formulation of Roundup used was Grand Travaux Plus (450 g/L glyphosate, approval 2020448; Monsanto, Belgium). The required level of Roundup dilution in drinking water was confirmed by measurement of glyphosate concentration by HPLC-MS/MS. Similarly, glyphosate stability in solution was studied and validated during the 7 day period between two preparations of the test, treatment solutions. Glyphosate and AMPA were not found in the feed at the limit of detection of 5 mg/kg.

### Toxicity analysis

Twice-weekly monitoring allowed careful observation and palpation of animals, recording of clinical signs, identification and measurement of any tumours, food and water consumption, and individual body weight. Measurement of mortality rates, anatomopathology (on 34 different organs), serum biochemistry (31 parameters) and urine composition (11 parameters) have been extensively described [17].

For transmission electron microscopy, liver fragments were fixed in pre-chilled 2 % paraformaldehyde/2.5 % glutaraldehyde in 0.1 M phosphate buffered saline pH 7.4 at 4 °C for 3 h and processed as previously described [18]. Evaluation of cell, cytoplasm and nuclear area, and nuclear-cytoplasmic ratio was determined from measurements on 30 hepatocytes per animal. Cell nuclear parameters (% heterochromatin, pore density, fibrillar centers area, % fibrillar centers, % dense fibrillar components and % granular components) were measured on 10 nuclei per animal.

### Tissue sampling and RNA extraction

Animals were sacrificed at the same time of day during the course of the study either to comply with animal welfare regulations to avoid unnecessary suffering (for example, resulting from 25 % body weight loss, presence of tumours over 25 % bodyweight, hemorrhagic bleeding, or prostration) or at the termination of the study period of 2 years. Animals were sacrificed by exsanguination under isoflurane anesthesia. The liver was divided into two portions; one was snap frozen in liquid nitrogen/dry ice and stored at −80 °C. One kidney was also snap frozen. (Note: due to handling errors at the company employed to conduct the experiment, one kidney sample from the Roundup treatment group was unavailable for analysis).

Transverse cross sectional slices of liver and kidneys were processed for total RNA extraction using MagMax-96 for Microarrays Total RNA Isolation Kit (Ambion, Life Technologies Ltd, Paisley, UK).

### Microarray hybridization

Total RNA (500 ng) was labelled using terminal deoxynucleotidyl transferase (TdT) in the presence of a proprietary biotinylated compound using the Ambion whole transcript Expression kit and the whole transcript Terminal Labelling kit (Affymetrix UK Ltd., High Wycombe, UK), following standard protocols. We employed the Affymetrix GeneChip® Rat Gene 2.0 ST Array containing approximately 610,400 probes grouped into 214,300 exon-level and 26,400 gene-level probe sets. The median number of probes per transcript is 22, usually distributed along the entire transcript sequence. Overall, this microarray covers 28,407 refseq transcripts including 16,771 protein coding transcripts. By comparison, version 5.0 annotation of the rat (strain BN/SsNHsdMCW) genome assembly contains 30,404 transcripts including 22,777 coding genes. The analysis was performed at the level of transcript clusters. We also used the MetaCore Analytical Suite to perform the transcription factor and the pathway and toxicity process analysis based on network objects recognized by MetaCore.

Hybridisation cocktails were applied to Affymetrix Rat Gene 2.0 microarrays, and processed in accordance with the manufacturer’s recommended procedure using the GCS3000 microarray system (Affymetrix). Array data was exported as cell intensity (CEL) files for further analysis.

### Microarray data analysis

CEL files were normalised together in the Expression Console software package (Affymetrix), using the Robust Multi-array Average (RMA) sketch algorithm (gene-level). Data was quality control assessed by using standard metrics and guidelines for the Affymetrix microarray system. Normalised data files (CHP files) were imported into Omics Explorer 3.0 (Qlucore) for further quality control and statistical analysis. It was decided to include all genes in the tests as there is no simple measure of presence/absence with RMA normalized data from Rat Gene 2.0 ST arrays. Although it risks a higher false discovery rate, all data collected were subjected to the statistical tests so as not to inadvertently filter out important genes based on an arbitrary detection threshold. Data used for the functional analysis were selected at the cut off values of *p* < 0.01 with FC >1.1 to use large gene lists as recommended [19]. The *q*-values, an estimate of the false discovery rate, were the *p*-values corrected using the Benjamini-Hochberg procedure and were below 8 %.

Gene ontology, pathways, gene networks, transcription factor binding and disease ontology were analyzed using the Thomson Reuters MetaCore Analytical Suite recognizing network objects (proteins, protein complexes or groups, peptides, RNA species, compounds among others) and/or the NIH Database for Annotation, Visualization and Integrated Discovery Bioinformatics Resources 6.7, recognizing individual coding genes, using recommended analytical parameters [19]. These microarray data have been submitted to Gene Omnibus and are accessible through accession number GSE66060.

### Microarray data validation by real-time qPCR

In order to validate the list of differentially expressed/regulated genes revealed by microarray analysis, we randomly selected a subset of 18 genes for analysis by real-time quantitative PCR (RT-qPCR) using the original RNA samples as starting material. Total RNA (2 μg) was converted to cDNA with the High Capacity RNA-to-cDNA kit (Applied Biosystems, Life Technologies Ltd, Paisley, UK), and gene expression measurements were assessed by RT-qPCR using TaqMan Gene Expression assays and TaqMan Universal PCR Master Mix (Applied Biosystems). Housekeeping genes used as endogenous controls were of two types: 1) three standard historically used housekeeping genes (*Gapdh*, *Hprt1*, *Actb*) (from the GeNorm list) as being among the most invariant transcript clusters from microarray datasets; that is, genes with the lowest standard deviation across all relevant samples, and with high or comparable expression level, and 2) *Pes1*, a gene which is not sex- and/or multi-hormone-regulated in rat studies [20]. Delta Ct methods were used to normalise the expression of the chosen genes of interest against the average of the endogenous control genes. Group fold changes and log ratios (relative to control groups) were calculated using the RQ method in DataAssist v3.0 software (Applied Biosystems).

## Results

### Tissue selection

The rat liver and kidney tissues that formed the starting material for this investigation were obtained from animals that formed part of a chronic (2 year) toxicity study looking at the effects of Roundup pesticide [17]. Roundup was administered via drinking water to Sprague–Dawley rats at a regulatory admissible dose (50 ng/L glyphosate equivalent dilution) and which is representative of what may be found in contaminated tap water. At this dose, the glyphosate equivalent average daily intake of Roundup was approximately 4 ng/kg bw/day of glyphosate.

Male animals suffered from liver and kidney damage more acutely than females, resulting in an increased rate of premature death [17]. Most male rats were discovered after death had occurred. This resulted in organ necrosis making them unsuitable for further analysis and thus they were excluded from transcriptome profiling. We therefore focused our investigation on female animals where freshly dissected tissues from cohorts of 9–10 euthanised treated and untreated rats were available.

Female control and Roundup-treated animals were respectively euthanized at 701 +/− 62 and 635 +/− 131 days (Additional file 1). Anatomopathological analysis of organs from these animals revealed that the liver and kidneys were the most affected organs [17]. Roundup-treated female rats showed 3 times more anatomical signs of pathology (15 in 8 rats) than the control group (6 in 4 rats). In addition, serum and urine biochemical analysis showed increased levels of serum triglycerides. While no severe anatomical kidney damage was detected, biochemical analysis revealed a decrease in blood Na, Cl, P and K levels and a corresponding increase in urine suggesting ion leakage and decreased urinary creatinine. Taken together these alterations in blood and urinary biochemical parameters suggest an impairment of kidney function. Overall, twice the number of biochemical parameters was disturbed in kidney than what can be expected by chance. Furthermore, a testosterone/estrogen imbalance was evident with testosterone serum levels significantly increased by 97 % by comparison to controls, while estradiol serum levels were decreased by 26 %. These observations together with pituitary gland disturbances suggest endocrine disrupting effects.

**Fig. 1:**
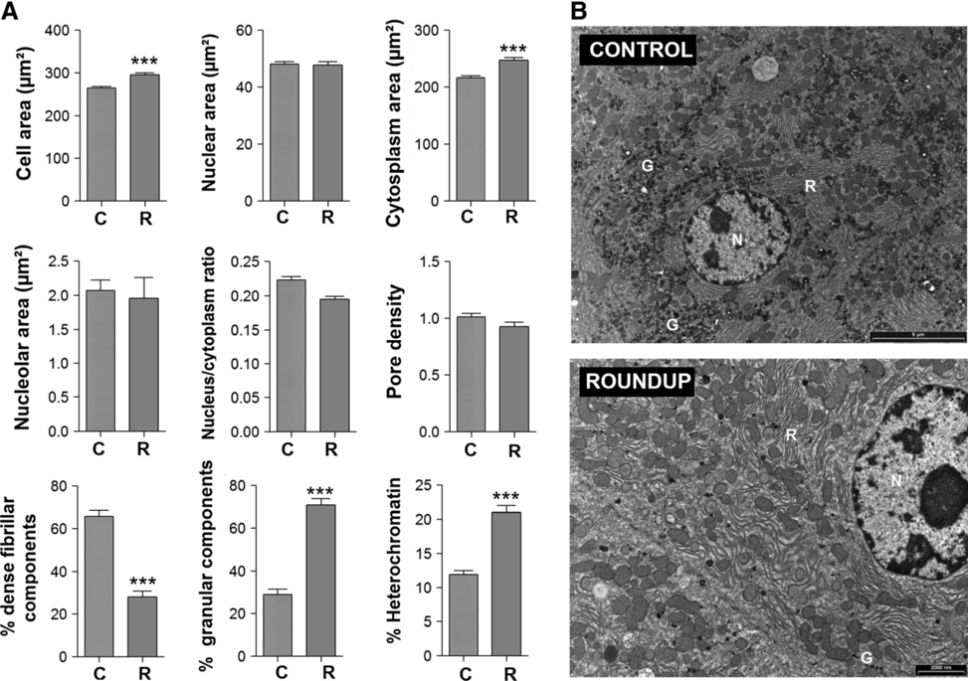
Alterations in hepatocyte nuclear architecture in female Roundup-treated rats suggests transcriptional disturbances. Liver from control (C) and Roundup (R) treated female rats were subjected to an ultrastructural electron microscopic analysis to investigate subcellular architecture. **a** Quantification of morphometric analysis of hepatocytes revealing alterations in subnuclear (heterochromatin, dense fibrillar, granular) compartments indicative of a reduced transcriptional status. Morphometric parameters are represented by their mean and their standard deviation. A two-tailed unpaired *t*-test was used as a standard test for statistical comparisons (***, *p* <0.001). **b** Representative electron micrographs comparing hepatocytes from control (*upper panel*) and Roundup-treated (*lower panel*) rats showing a disruption of glycogen dispersion (G). *N* nucleus, *R* rough endoplasmic reticulum

Electron microscopic analysis of liver sections from these animals showed nucleolar disruption in hepatocytes (Fig. 1). There was a statistically significant increase of cell and cytoplasmic area. No major cell structural damage was observed. However, the nuclear morphometry analysis showed that hepatocytes of Roundup-treated female rats had a statistically significant higher heterochromatin content, a reduced dense fibrillar component and a concomitant increase in granular component in comparison to controls indicating a disruption of nucleolar function and overall decreased level of transcription (Fig. 1a). In addition, a cytoplasmic dispersion of glycogen was also observed in the Roundup treated group (Fig. 1b).

### Transcriptome patterns segregate liver and kidney samples based on Roundup treatment

**Fig. 2:**
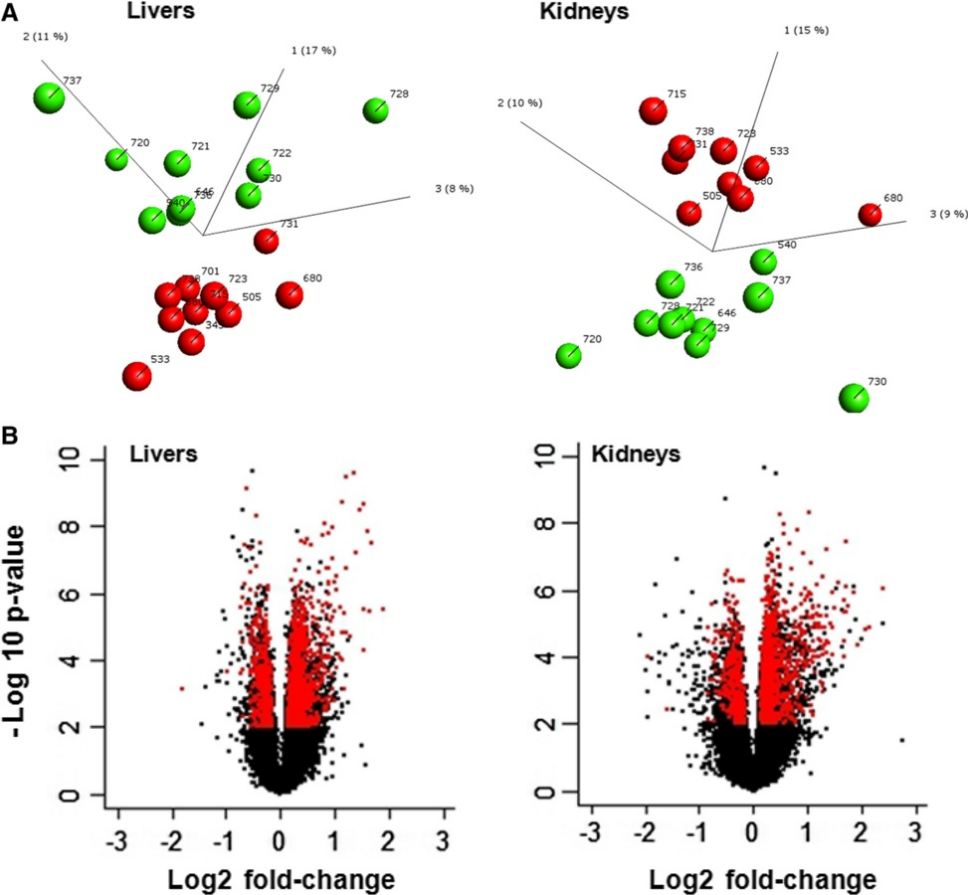
Wide-scale transcriptome profile alteration in liver and kidneys of Roundup-treated female rats. Liver and kidneys from control rats and animals receiving 0.1 ppb Roundup (50 ng/L glyphosate equivalent dilution) in drinking water were subjected to a full transcriptome microarray analysis. **a** PCA analysis of transcript cluster expression profiles shows a distinct separation into groups of treated (*red*) and control (*green*) rats in both kidney and liver samples. Numbers by data points denote age at time of death in days. **b** Volcano plots of the liver and kidney transcriptome profiles showing the log 2 fold changes and the –log10 *p*-values in transcript cluster expression induced by Roundup exposure compared to controls. Data were selected at the cut off values *p* <0.01 and fold change >1.1. *Red dots* represent genes commonly disturbed between liver and kidney samples

In an attempt to confirm the anatomopathological effects in a more quantitative manner and to gain insight into the gene expression profiles that are associated with the signs of pathology observed in the liver and kidneys, we conducted a full transcriptome microarray analysis of these organs. We began by undertaking an unsupervised Principal Component Analysis (PCA) of the dataset, which reduces a high-dimensional expression profile to single variables (components) retaining most of the variation (Fig. 2a). As control and Roundup-treated animals were sacrificed at various ages, we initially conducted a PCA analysis where individual animals were sorted by age (oldest vs youngest), to ascertain if differences in transcriptome profiles correlate with this parameter (Additional file 2). This showed that the statistical control values were weak, with no segregation of the oldest and youngest animals in either control or Roundup-treated groups, indicating that age was not a major source of difference. By contrast, a clear separation was observed between control and Roundup-treated rats based on treatment (Fig. 2a; Additional files 3 and 4). Even if the principal components only account for ~30 % of the observed variation, which seems quite low, this high level view of the data showed a homogeneous segregation and a low intragroup variability, as well as the absence of outliers. Figure 2b shows the statistical significance (by Student’s t-tests) of differential transcript cluster expression by volcano plots along with respective fold changes (FC) (Fig. 2b), where a transcript cluster constitutes a group of exon clusters (each exon cluster composed of different probes) corresponding to a known or putative gene. Overall, gene expression changes varied from −3.5 to 3.7 fold in liver and from −4.3 to 5.3 in kidneys. The expression of 57 and 226 transcript clusters were respectively disturbed in liver and kidney over an FC of 2. *Akr1b1* (*FC of* −*4.3*, *p* = *2.2E*-*5*) *and Ten1* (*FC of* −*3.5*, *p* = *7.3E*-*4*) were the most down-regulated genes respectively in liver and kidneys The most up-regulated transcripts were small nucleolar RNAs (snoRNAs), *ENSRNOT00000053015* in liver (FC = 3.7, *p* = 3.0E-6) and *ENSRNOT00000068958* in kidneys (FC = 5.3, *p* = 8.6E-7). Large statistical significance (*p*-values up to 2.3E-10 in liver) was observed. A large number of transcript clusters were significantly disturbed below stringent *q*-value thresholds (Table 1). In addition, the statistical analysis of simulated random samples confirms that the degree of statistical difference between control and Roundup treatment groups are far greater than what can be expected by chance (Table 1).

**Table 1:** Number of transcript clusters whose expression is disturbed at different cut-off threshold *p*-values

*p*-value	Liver	Kidney	Random
0.05	8606^(0.21)^	8656^(0.21)^	1835^(0.98)^
0.01	4224^(0.08)^	4447^(0.08)^	380^(0.96)^
0.001	1593^(0.02)^	1894^(0.02)^	31^(0.95)^
0.0001	630^(0.006)^	764^(0.005)^	1^(0.95)^
0.00001	230^(0.002)^	219^(0.002)^	0

The number in superscript parenthesis is the maximal *q*-value (calculated using Benjamini-Hochberg method according to corresponding to the number of genes found disturbed at increasing (0.05 to 0.00001) *p*-value stringency. A statistical analysis of simulated random samples was also performed to estimate effects that would be expected to arise by chance

**Fig. 3:**
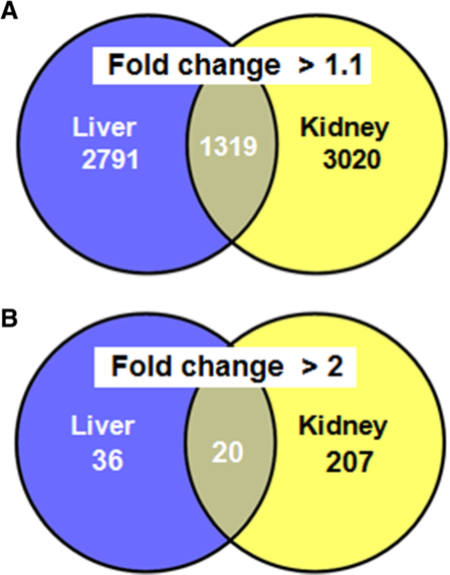
Large spectrum of transcript cluster expression is commonly disturbed in liver and kidney by Roundup. Venn diagrams showing numbers of genes commonly disturbed in liver and kidneys as revealed by transcriptome analysis at cut off threshold values of *p* <0.01 and fold change >1.1 (**a**) and >2 (**b**)

Data used in functional analysis were selected at the cut off values of *p* < 0.01 and *q* < 0.08 with FC >1.1 for use with large gene lists as previously recommended [19]. A Venn diagram comparing liver and kidney transcript cluster expression profiles at an FC >1.1 (Fig. 3a) indicates that even if most of the disturbances were tissue specific, 1319 transcript clusters were commonly disturbed between the two organs. A comparison at a frequently selected cut off threshold of FC > 2 again results with most changes in gene expression being specific to either liver or kidney but with a total of 20 genes being commonly disturbed in both organs (Fig. 3b). The FC, *p*-value and *q*-value for all genes whose expression is commonly disturbed in both liver and kidney are shown in Additional file 5.

Out of these, 18 genes were randomly selected for validation of the microarray results by RT-qPCR (Additional file 6). The overall pattern of the RT-qPCR results confirmed the microarray data with 86 % of the genes found to be similarly up- or down-regulated by both methods.

### Gene function alterations involved in mitochondrial respiration, spliceosome function and chromatin structure modification is associated with Roundup treatment

We next conducted an ontology analysis of the 1319 transcript clusters commonly deregulated in liver and kidney using the DAVID gene functional classification tool to reveal the most affected gene categories. As a result 868 genes were recognised. The 8 clusters of functional disturbances having enrichment scores over 2 are presented in Table 2. All have significant *p*-values and Benjamini corrected *p* value (*q*-values). A total of 3 major affected gene networks were identified.

**Table 2:** Functional clustering of genes derived using the DAVID gene functional classification tool

ES	Term in DAVID	n	*p* value	FE	*q*-value
4.2	GO:0010558 ~ negative regulation of macromolecule biosynthetic process	40	3.7E-05	2.0	7.9E-3
	GO:0031327 ~ negative regulation of cellular biosynthetic process	40	6.0E-05	2.0	1.1E-2
	GO:0009890 ~ negative regulation of biosynthetic process	40	9.3E-05	1.9	1.6E-2
3.8	GO:0016279 ~ protein-lysine N-methyltransferase activity	7	5.0E-05	9.7	5.1E-3
	GO:0016278 ~ lysine N-methyltransferase activity	7	5.0E-05	9.7	5.1E-3
	GO:0018024 ~ histone-lysine N-methyltransferase activity	7	5.0E-05	9.7	5.1E-3
	GO:0042054 ~ histone methyltransferase activity	7	3.0E-04	7.2	2.3E-2
	GO:0008276 ~ protein methyltransferase activity	7	2.5E-03	5.0	7.7E-2
3.3	GO:0000377 ~ RNA splicing, via transesterification reactions with bulged adenosine as nucleophile	15	4.8E-04	3.0	6.0E-2
	GO:0000375 ~ RNA splicing, via transesterification reactions	15	4.8E-04	3.0	6.0E-2
	GO:0000398 ~ nuclear mRNA splicing, via spliceosome	15	4.8E-04	3.0	6.0E-2
3.3	IPR000504:RNA recognition motif, RNP-1	17	4.0E-04	2.8	3.1E-1
	SM00360:RRM	17	5.1E-04	2.7	9.8E-2
	IPR012677:Nucleotide-binding, alpha-beta plait	17	6.1E-04	2.7	2.5E-1
2.3	GO:0006099 ~ tricarboxylic acid cycle	6	1.7E-03	6.6	1.5E-1
	GO:0046356 ~ acetyl-CoA catabolic process	6	2.1E-03	6.3	1.7E-1
	GO:0009109 ~ coenzyme catabolic process	6	3.7E-03	5.6	2.3E-1
	GO:0009060 ~ aerobic respiration	6	4.4E-03	5.4	2.5E-1
	GO:0051187 ~ cofactor catabolic process	6	8.1E-03	4.7	3.4E-1
	rno00020:Citrate cycle (TCA cycle)	6	9.2E-03	4.5	1.7E-1
	GO:0006084 ~ acetyl-CoA metabolic process	6	1.9E-02	3.8	4.7E-1
2.2	GO:0030964 ~ NADH dehydrogenase complex	5	3.7E-03	7.5	7.1E-2
	GO:0045271 ~ respiratory chain complex I	5	3.7E-03	7.5	7.1E-2
	GO:0005747 ~ mitochondrial respiratory chain complex I	5	3.7E-03	7.5	7.1E-2
	GO:0005746 ~ mitochondrial respiratory chain	5	4.3E-02	3.7	3.1E-1
2.1	GO:0016571 ~ histone methylation	6	1.4E-03	6.9	1.3E-1
	GO:0008213 ~ protein amino acid alkylation	6	1.9E-02	3.8	4.7E-1
	GO:0006479 ~ protein amino acid methylation	6	1.9E-02	3.8	4.7E-1
2.1	IPR001440:Tetratricopeptide TPR-1	9	4.5E-03	3.4	5.8E-1
	IPR019734:Tetratricopeptide repeat	9	1.0E-02	2.9	7.2E-1
	SM00028:TPR	9	1.2E-02	2.8	5.7E-1

The rat genome was used as a background list to calculate the *p*-values of each term. A total of 868 genes were recognised. The *p*-values were calculated according to a modified Fisher’s exact test (EASE score). The *q*-values were calculated according to the Benjamini-Hochberg method. Cluster enrichment scores (ES) and fold enrichment (FE) rank overall importance (enrichment) of gene groups or the statistically most overrepresented (enriched) biological annotations. The highest classification stringency was used

First, two clusters were related to spliceosome function. This included genes encoding cleavage and polyadenylation specific factors (*Cpsf2*, *Cpsf3* and *Cpsf7*), heterogeneous nuclear ribonucleoproteins (*Hnrnpl*, *Hnrnpf*) and splicing factors (*Sf3b5*, *Sf3a1*). Expression of all of these genes was upregulated with the exception of *Cpsf3*. Other genes involved in RNA splicing, such as *Luc7l3*, *Pnn*, *Prpf4b*, *Pnisr*, *Prpf39*, *Srek1*, *Ddx39b* and *Ddx39a*, were significantly upregulated. Additionally, expression of at least 160 non-coding snoRNAs were found to be altered with almost all being upregulated with a large FC of up to 5.32 for ENSRNOT00000068958in kidneys. Second, two clusters consisted of members of the chromatin modification family of enzymes, in particular histone-lysine N-methyltransferases. Expression of the 7 genes (*Men1*, *Setdb1*, *Suv420h2*, *Dot1l*, *Ehmt1*, *Ehmt2*, *Nsd1*) belonging to this cluster was upregulated. Other genes with a related ontology (*Mll2*, *Mll4*, *Tet3*, *Baz2a*, *Dnmt3a*, *Brd1*, *Brd4*, *Ino80d* or *Arid4b*), which are not taken into account in the cluster of enriched biological functions, were also upregulated. Most also belong to the family of histone-lysine N-methyltransferase complexes that specifically methylate lysine residues of histone H3 (Lys-4, 9, 20 or 79) or H4 (Lys-20) among others, tagging them for chromatin condensation. In addition, most of these are also included in a larger disturbed cluster of 40 genes involved in negative regulation of macromolecule biosynthetic processes.

Third, functional disturbance of genes involved in mitochondrial metabolism was represented by two clusters, especially related to respiratory chain complex I and the TCA cycle. Expression of most of these genes was repressed. A total of 7 genes encoding NADH dehydrogenase (ubiquinone) complex I of the mitochondrial respiratory chain were found disturbed, with 6 of them being downregulated. Additionally, the genes encoding isocitrate dehydrogenases (*Idh3B* and *Idh3g*), succinate dehydrogenase (*Sdhc*), succinate-CoA ligases (*Sucla2* and *Suclg2*) and mitochondrial F1 complex ATP synthases (*Atp5b* and *Atp5d*) were downregulated. These data suggest that activities of mitochondrial complexes, in particular respiratory activity, are depressed.

**Fig. 4:**
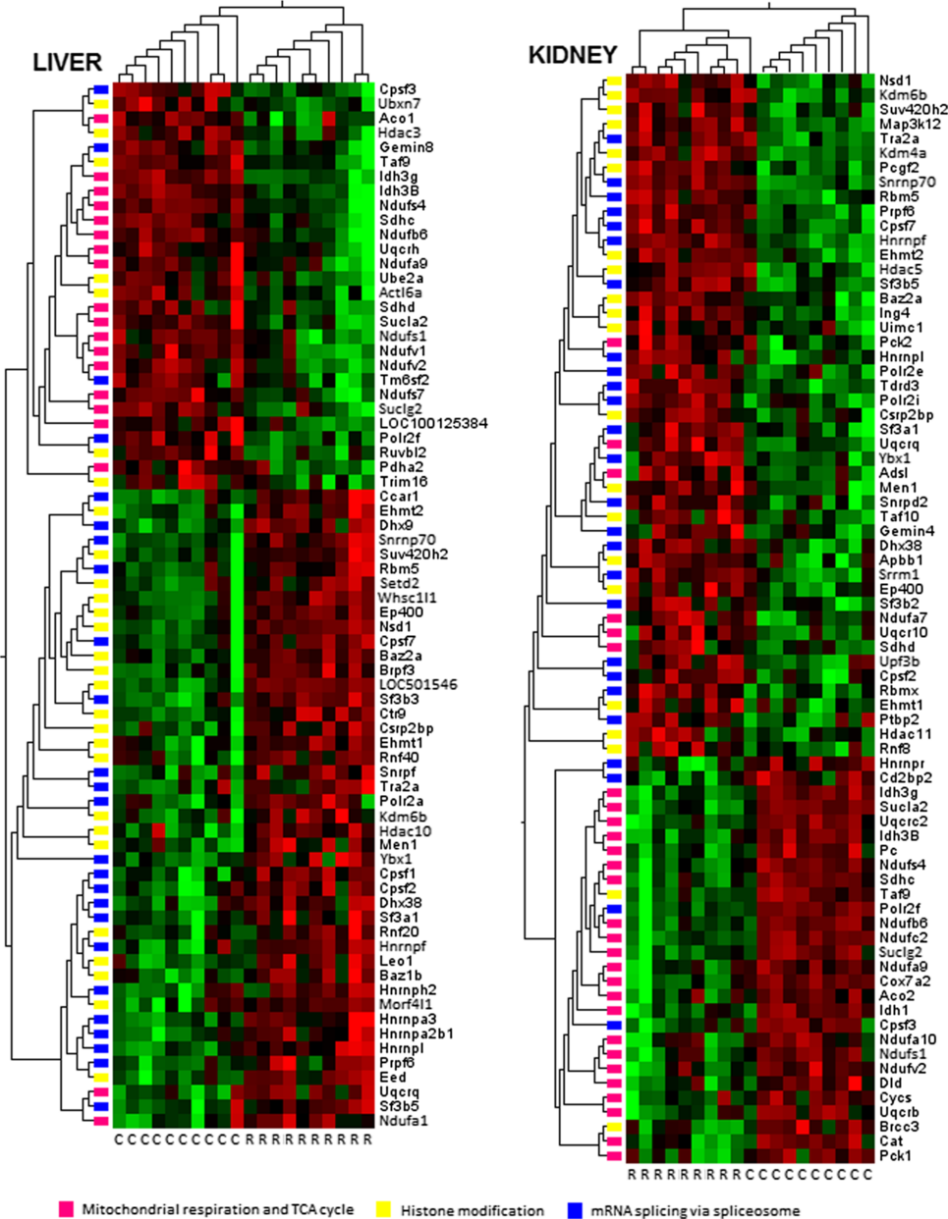
Heatmaps of the three major ontologically enriched biological functions from transcriptome analysis of liver and kidneys. The ontologically enriched biological functions (Table 2) derived from the alteration in gene expression patterns commonly disturbed in liver and kidneys from Roundup treated female rats (Figs. 2 and 3) with respect relative to mRNA splicing via spliceosome (GO:0000398, in *blue*), histone modification (GO:0016570, in *yellow*) and cellular respiration and TCA cycle (GO:0045333 and GO:0006099, in *pink*), were grouped on organ-specific heatmaps using hierarchical clustering of samples (C, control; R, Roundup) and variables (gene symbols). A distinct separation based on direction (up- or down-regulation) of gene expression, biological function and organ between Roundup-treated and control animals is discernible

These 3 major enriched biological functions are presented by organ-specific heat maps using hierarchical clustering of samples (Fig. 4). Our results show genes related to mitochondrial respiration and the TCA cycle are mostly repressed while those involved in mRNA splicing and histone modification are upregulated.

### Roundup-associated changes may occur via sex-hormone signaling pathways

The gene ontology analysis (Table 2) indicates a modulation of cell signalling pathways has taken place. The GO biological processes GO:0007264 “small GTPase mediated signal transduction” (21 genes, *p* = 1.2E-3, *q* = 1.2E-1) and GO:0007242 “intracellular signalling cascade” (57 genes, *p* = 2.2E-3, *q* = 1.7E-1) are enriched among genes commonly disturbed in liver and kidneys. Additionally, the networks highlighted by this analysis that may account for the disturbance in gene expression were centred on the transcription factors Creb1 (280 genes regulated, *p* ~ 0), c-Myc (159 genes regulated, *p* ~ 0), Yy1 (113 genes regulated, *p* <4.8E-234), Oct3/4 (94 genes regulated *p* <6.7E-194) and Esr1 (83 genes regulated, *p* <8.E-171) (Additional file 7). These transcription factors are intimately connected in regulation of gene expression and can be involved in hormone signalling pathways.

In this context, it is noteworthy that the gene encoding the androgen receptor is statistically significantly downregulated in liver (FC = −1.4, *p* = 8.1E-3, *q* = 7.7E-2) and kidneys (FC = −1.32, *p* = 2.9E-3, *q* = 3.8E-2). In addition, the retinoid X receptor beta gene (*Rxrb*) is significantly upregulated in liver (FC = 1.2, *p* = 3.5E-5, *q* = 3.2E-3) and kidneys (FC = 1.36, *q* = 4.5E-6, *q* = 1.2E-3). In addition, the KEGG annotation for the mTOR signalling pathway (14 genes, *p* = 6.4E-3, *q* = 0.21) and the phosphatidylinositol signalling system (16 genes, *p* = 1.3E-2, *q* = 0.29) are enriched in liver. In kidney, the KEGG annotation for the adipocytokine signalling pathway (involving mTOR) (19 genes, *p* = 2.4E-3, *q* = 0.06) and the phosphatidylinositol signalling system (21 genes, *p* = 6E-4, *q* = 0.05) are enriched. However, expression of *Star*, which has been suggested by previous studies to mediate endocrine disrupting effects of Roundup in MA-10 Leydig tumor cells [21], and of *Esr1*, *Esr2* and aromatase (*Cyp19a1*), which were found disturbed in the human HepG2 hepatocyte cell line [22], were not found to be dysregulated in this investigation.

### Alterations in transcriptome profile suggest liver and kidney anatomorphopathogy

**Fig. 5:**
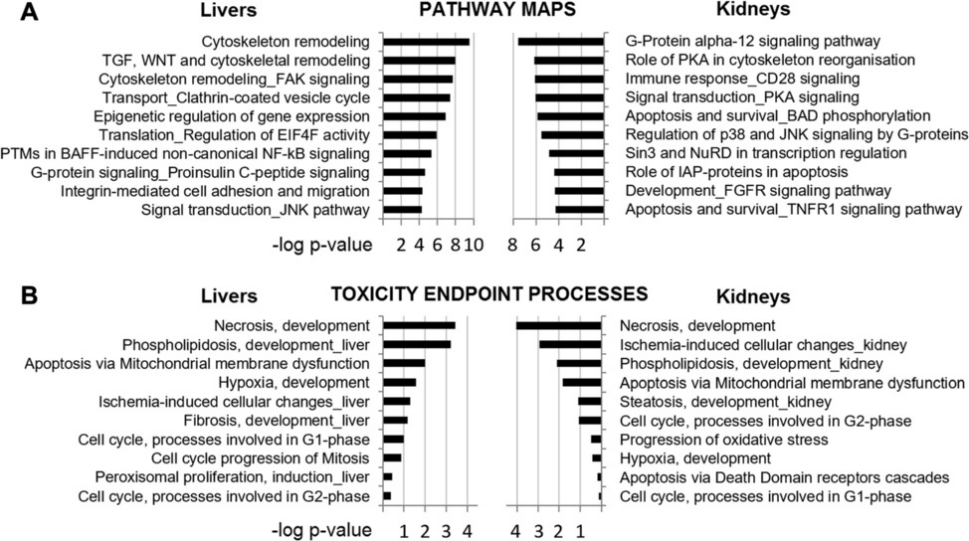
Toxicity ontology analysis of genes disturbed in liver and kidneys of Roundup-treated rats. List of top 10 scoring pathway and toxicity process networks revealed by MetaCore analysis of female liver and kidney transcriptome profiles receiving 0.1 ppb of Roundup in drinking water (*p* <0.01, fold changes >1.1). The *p*-values are determined by hyper-geometric calculation

Scoring maps for pathways and toxicity processes (Fig. 5) indicates that multiple cellular functions could be involved. Out of the 4224 liver and 4447 kidney transcript clusters found to be altered, 2636 and 2933 network objects were respectively recognized by GeneGO Metacore. Mapped pathways related to inflammatory responses, which can be secondary outcomes to organ damage, are enriched (for instance, those involving NF-κB or CD28 signalling). Various pathways associated with the cytoskeleton are also enriched, suggestive of a change in cellular growth in an effort to overcome toxic effects and to regenerate damaged tissues. In this regard the enrichment of the proinsulin C-peptide signalling pathway in liver (14 genes, *p* = 2.4E-5, *q* = 1.2E-3) involving mTOR and the phosphatidylinositol signalling systems is of note since it has an established role in cellular proliferation and lipid metabolism. Other maps confirmed the induction of intracellular signalling pathways and an influence on the balance between proliferation and apoptosis. The regulation of translation by EIF4F activity (16 genes, *p* = 1.2E-6, *q* = 8.1E-5), another mTOR regulated function, is also disturbed.

Furthermore, the GO “metabolic process” and “cellular response to stress” processes have low *p*-values in liver (respectively *p* = 3.7E-58 and 3.9E-16) and kidneys (*p* = 1.5E-49 and 1.0E-14), which strongly suggest a state of metabolic stress. Overall, toxicity process analysis revealed gene expression disturbances associated with apoptosis, necrosis, phospholipidosis, mitochondrial membrane dysfunction and ischemia. Thus the alteration in the transcriptome profile identified in this study correlates with the observed increased signs of anatomical and functional pathology of the liver and kidneys.

## Discussion

We report here the first in vivo transcriptome investigation in a mammalian species following long-term (2 year) exposure to an agricultural GBH (Roundup) at an environmentally relevant dose. Our results confirm the increased incidence of liver and kidney pathologies described at an anatomorphological and blood/urine biochemical level in female rats administered with Roundup in drinking water at a regulatory admissible, ultra-low dose 50 ng/L glyphosate equivalent concentration [17]. The levels of glyphosate consumption were approximately 4 ng/kg bw/day, which are well below global ADI values. We observed a wide-scale, treatment-associated alteration in gene expression patterns at a high statistical significance in both the liver and kidneys (Figs. 2 and 3; Table 1). Gene ontology analysis of these transcriptome alterations is linked with a marked change in mitochondrial respiration, spliceosome activity, chromatin structure and hormone signalling pathways (Table 2). Collectively, the alterations in gene expression (Fig. 4) are associated with a deregulation of tissue homeostasis at the level of proliferation-apoptosis balance (Fig. 5) and thus correlates well with the increased signs of liver and kidney anatomical, histological and blood/urine biochemical pathologies described in these animals [17]. Also of note is that the Roundup-treatment associated alterations in gene expression patterns we observe do not correspond to transcriptome signatures of liver necrosis provoked by acute hepatotoxicants [23].

Following the recommended analytical approach for large sets of genes [19], we observed a large number (>4000) whose expression was altered in both the liver and kidneys within the Roundup treatment group (Fig. 3a). In the majority of cases the change in level of transcription was below 2-fold (Fig. 3a and b) but to a highly statistically significant degree (*p* <0.01). These observations imply that low but consistent changes in expression of a large number of genes can provide sufficient statistical resolution to be informative regarding any organ pathology that may be present.

However, given the large number of gene functions altered in both the liver and kidneys within the Roundup treatment group, this represents a combination of effects resulting from the pathology of these organs as well as a direct impact of the pesticide. Thus it is not possible from our investigation to definitively distinguish the primary effects of Roundup on the liver and kidney transcriptome from the secondary effects on gene expression arising from the pathology present in these organs. Nevertheless, the smaller cohort of genes found to be commonly disturbed in liver and kidney (Fig. 3; Additional file 5) may provide insight into those systems that may be the primary targets of this herbicide (Table 2). Our results highlight the need for future GBH toxicity studies where organ transcriptome is determined prior to appearance of the overt liver and kidney pathologies observed at late-stage termination as in this instance. Thus transcriptome disturbances that may ultimately lead to the later stage organ pathologies may be identified. In addition, the clinical relevance of our observations remains to be ascertained, particularly as there is limited data available on glyphosate levels within humans [4].

Findings from studies where mice were fed diets containing Roundup-tolerant genetically modified soybeans [18, 24] are consistent with our observations. Animals showed disruptions in hepatocyte nuclear architecture, decreased expression of certain respiratory enzymes, a disturbance of splicing activity and marked increased liver ageing. In addition, similar observations were made with rat hepatocytes treated with Roundup in vitro [25], suggesting that alterations in nucleolar and mitochondrial function may be a direct primary effect of this herbicide.

Previous studies, albeit at much higher doses, have shown that glyphosate can uncouple liver mitochondrial oxidative phosphorylation [6] and induce non-specific membrane permeabilization and a depletion of succinate-dependent respiratory indexes in isolated rat mitochondria [26]. The mode of glyphosate inhibition of EPSPS in plants is by competitive inhibition of phosphoenolpyruvate (PEP) substrate binding at the enzyme active site [5]. Enzymes binding PEP are regulators of energy metabolism in particular through the TCA cycle. Glyphosate off-target effects may include the disruption of these enzymes. Indeed, glyphosate can interact at the substrate binding site and potentially inhibit mitochondrial succinate dehydrogenase [27]. Furthermore, as small molecule chelators of zinc can perturb spliceosome assembly and activity of chromatin modifying enzymes [28], glyphosate may also have exerted direct effects on spliceosome function due to its metal chelating properties (Patent No: US 3160632; [29]).

The increased incidence of Roundup-associated liver and kidney pathologies [17] confirmed in this report may be arising from multiple sources as there is increasing evidence to suggest that GBH and glyphosate can bring about toxic effects via different mechanisms depending upon the level of exposure. However, toxic effects have been recorded in most instances at levels of glyphosate and/or GBH exposures [10, 15] far in excess of the ultra-low dose administered to the animals in this investigation. Thus it is difficult to definitively attribute one or more mechanisms of toxicity observed at these higher dose levels to the liver and kidney pathologies seen in our study. Nevertheless, our observation of a major accumulation of snoRNAs in both liver and kidneys of the Roundup-treated group (Fig. 3, Table 2) supports the possibility of damage arising from oxidative stress as these are known to play a critical role in amplifying the effects of reactive oxygen species and downstream oxidative stress-mediated tissue injury [30]. The study by Michel and colleagues demonstrated the induction of snoRNA expression as a functional link between the progress of lipotoxic cell death and the deleterious cellular response to oxidative stress [30]. Lipotoxicity manifests itself as enhanced oxidative stress and as elevated proinflammatory signalling, often associated with abnormal insulin action [31]. It is noteworthy that we found disturbances in the pathways associated with these biological processes in our study (Fig. 5).

In addition, the analysis of pathways revealed disturbances in the modulation of mTOR and phosphatidylinositol signalling systems, both linked to the regulation of lipid synthesis among other major cellular functions [32]. Phosphoinositide 3-kinase transduces signals from various factors into intracellular messages by generating phospholipids, the disruption of which is among the major toxicity endpoint processes observed in the liver and kidneys in this study (Fig. 5). Overall, these results suggest an involvement of lipotoxic stress associated with elevated oxidative stress and proinflammatory signalling, which is a hallmark of chronic liver and kidney disease [33].

Chronic lipotoxic conditions in our study are also corroborated by biochemical analysis showing increased levels of serum triglycerides [17]. In some circumstances, excess free fatty acids induce apoptotic cell death [33]. Our analysis also indicates changes in intracellular pathways possibly underlying a change in cell growth, such as the proinsulin C-peptide signalling pathway as well as the mTOR and the phosphatidylinositol signalling systems. Tissue homeostasis relies on a delicate balance between apoptosis and cellular proliferation. When oxidative stress provokes apoptosis, injured cells are generally replaced through increased proliferation. Interestingly, similar results have recently been reported where transcriptome analysis of *brown trout* exposed to glyphosate or Roundup at a concentration (10 μg/L) typically found in the environment, revealed an over-representation of pathways involved in oxidative stress, apoptosis, and the regulation of cell proliferation [34]. However, although there is evidence to suggest that glyphosate can inhibit multiple cellular functions such as mitochondrial respiration, and enzyme activities other than EPSPS within the shikimate pathway, the existence of such direct mechanisms of interference at the low environmental dose tested here currently remains unknown and thus needs further exploration.

The proliferation/apoptosis imbalance may also have been either caused or promoted by a chronic disruption of the endocrine system and the activation or repression of signalling pathways. Activation of phosphoinositide 3-kinase (PI3K) by hormones such as estrogens [35] results in a global downregulation of genes encoding members of the TCA cycle and oxidative phosphorylation, defective mitochondria, and reduced respiration [36]. Hormones and their nuclear receptors have dynamic interactions with chromatin remodeling complexes and spliceosome function [37]. As we have observed a disturbance in estrogen/testosterone balance and pituitary dysfunction in the animals studied here [17], a link between the disturbed gene expression patterns and pathologies via an endocrine disruptive mechanism is plausible, especially as endocrine disruptive effects can occur at very low doses [38]. Furthermore, the top-scoring transcription factor networks that can account for the observed Roundup-associated alterations in gene expression presented here are centered on Creb1, Esr1, Yy1, c-Myc and Oct3/4 (Fig. 4), which cooperate to regulate gene expression following hormonal stimulation [39]. Hormonal imbalance is generally associated with kidney or liver failure [40]. Furthermore, glyphosate has been found to act as an estrogen agonist in human breast cancer cell assays stimulating growth at comparable concentrations to the native hormone [13, 41]. This estrogenic potential of glyphosate may have contributed to the trend in increased mammary tumor incidence in the Roundup-treated rats analysed in this investigation [17].

An important consideration is that Roundup is not a single compound, but a mixture of an active ingredient (glyphosate) combined with various adjuvants, which are required to stabilise and allow penetration of glyphosate into plants. In short term acute exposures, some adjuvants can be considered as responsible of Roundup toxicity [42]. However, as adjuvant composition is proprietary and not fully disclosed, it is not possible to attribute the toxicity of the whole agricultural herbicide formulation to a given component. Thus the results from our study are not directly comparable to others testing glyphosate alone. The results we present may be specific for the formulation studied, because the toxicity of different GBH adjuvants can vary by a factor 100 at least based on assays involving a 24 h exposure to human tissue culture cells [42]. Future studies involving the administration of glyphosate alone would shed light on this issue.

In summary, the alterations in transcriptome profile were detected well below the glyphosate ADI (0.3 mg/kg bw/day) set within the European Union, and is within the range admitted in drinking water (0.1 ppb) and foodstuffs (for example, 20 ppm in GM soybeans or 2 ppm in bovine kidneys). As a probable endocrine disruptor, GBH/glyphosate may alter the functioning of hormonal systems and gene expression profiles via different mechanisms depending on the dose. Future metabolomic and proteomic studies of these organs could provide further mechanistic insight into the observed Roundup-mediated pathological process.

## Conclusions

It was previously known that glyphosate consumption in water above authorized limits may provoke kidney failure and reproductive difficulties [43]. The results of the study presented here indicate that consumption of far lower levels of a GBH formulation, at admissible glyphosate-equivalent concentrations, are associated with wide-scale alterations of the liver and kidney transcriptome that correlate with the observed signs of hepatic and kidney anatomorphological and biochemical pathological changes in these organs [17]. In addition, as the dose of Roundup we investigated is environmentally relevant in terms of human [4], domesticated animals [12] and wildlife [34, 44] levels of exposure, our results potentially have significant health implications for animal and human populations. Furthermore, data also suggests that new studies incorporating testing principles from endocrinology and developmental epigenetics, in particular to evaluate the endocrine disruptive capability of GBH/glyphosate, should be performed to investigate potential consequences of low dose exposure during early life as well as in adults.

## Additional files

Additional file 1:
**Metadata.** Metadata of the liver and kidney samples used for the microarray analysis including age of rats at the time of death via euthanasia. (XLSX 10 kb) Additional file 2:
**PCA analysis fails to reveal a correlation between alterations in transcriptome profile and age at the time of death.** Groups of 5 rats from the control and Roundup treatment categories were generated so that 5 animals with an earlier time of death (yellow spheres) were compared to 5 that were euthanised at a latter timepoint (purple spheres) in a PCA analysis. No significant differences in transcript cluster expression profiles were observed on this basis with samples from earlier and latter death timepoints being intermixed. For example, for livers, the transcript cluster expression profile of 10 rats with an earlier death timepoint (609 +/− 125 days) taken from controls (5 rats) and Roundup treated groups (5 rats) compared to the 10 animals with latter death (727 +/− 11 days) had a *q*-value of 0.99. (DOCX 42 kb) Additional file 3:
**Video of the PCA analysis shows a distinct separation into groups of treated (red) and control (green) rats in liver samples.** (WMV 1131 kb) Additional file 4:
**Video of the PCA analysis shows a distinct separation into groups of treated (red) and control (green) rats in kidney samples.** (WMV 1246 kb) Additional file 5:
**Common transcript cluster disturbances in liver and kidneys of rats receiving 0.1 ppb Roundup in drinking water.** ID is the Affymetrix transcript cluster identification number, FC indicates the fold change (upregulated transcript clusters highlighted in red; downregulated transcript clusters highlighted in green), P is the *p*-value (two-sided *t*-test) and q the false discovery rate (computed using the Benjamini-Hochberg method). The most significant differences (*p* <0.001) are highlighted in grey and were generated at the cut off values *p* <0.01 and FC >1.1 by using Qlucore Omics Explorer 3.0. Genes with fold change in expression >2 are highlighted in yellow. (DOCX 180 kb) Additional file 6:
**Microarray data is confirmed by RT-qPCR analysis.** A total of 18 genes were randomly selected among the 1319 transcript clusters whose expression was commonly up- or downregulated in liver and kidneys as shown by microarray analysis, were chosen for validation by RT-qPCR. Data from the microarray analysis is depicted in grey bars and that from the RT-qPCR in black bars. The RT-qPCR was performed by TaqMan assay in quadruplicate and standardised against 4 reference genes (*Gapdh*, *Hprt1*, *Actb* and *Pes1*). Two-tailed Student’s *t*-test was performed comparing the Roundup-treated group to their respective controls (* *p* <0.05, ** *p* <0.01, ****p* <0.001). The overall pattern of the RT-qPCR confirmed the microarray analysis results. (DOCX 38 kb) Additional file 7:
**Transcription factor networks associated with transcriptional alteration of genes commonly disturbed in liver and kidneys.** The list of transcript clusters commonly disturbed in liver and kidneys is used for generation of networks using Transcription Regulation algorithm with default settings with MetaCore. (TIFF 4891 kb)

## References

[1] Bohn T, Cuhra M, Traavik T, Sanden M, Fagan J, Primicerio R (2014). Compositional differences in soybeans on the market: glyphosate accumulates in Roundup Ready GM soybeans. Food Chem.

[2] EFSA (2014). The 2011 European Union report on pesticide residues in food. EFSA J.

[3] Majewski MS, Coupe RH, Foreman WT, Capel PD (2014). Pesticides in Mississippi air and rain: a comparison between 1995 and 2007. Environ Toxicol Chem.

[4] Niemann L, Sieke C, Pfeil R, Solecki R (2015). A critical review of glyphosate findings in human urine samples and comparison with the exposure of operators and consumers. J Verbr Lebensm.

[5] Williams AL, Watson RE, Desesso JM (2012). Developmental and reproductive outcomes in humans and animals after glyphosate exposure: a critical analysis. J Toxicol Environ Health B Crit Rev.

[6] Olorunsogo OO, Bababunmi EA, Bassir O (1979). Effect of glyphosate on rat liver mitochondria in vivo. Bull Environ Contam Toxicol.

[7] Olorunsogo OO (1990). Modification of the transport of protons and Ca2+ ions across mitochondrial coupling membrane by N-(phosphonomethyl)glycine. Toxicology.

[8] De Liz Oliveira Cavalli VL, Cattani D, Heinz Rieg CE, Pierozan P, Zanatta L, Benedetti Parisotto E (2013). Roundup disrupted male reproductive functions by triggering calcium-mediated cell death in rat testis and sertoli cells. Free Radic Biol Med.

[9] Larsen K, Najle R, Lifschitz A, Virkel G (2012). Effects of sub-lethal exposure of rats to the herbicide glyphosate in drinking water: glutathione transferase enzyme activities, levels of reduced glutathione and lipid peroxidation in liver, kidneys and small intestine. Environ Toxicol Pharmacol.

[10] Benedetti AL, Vituri Cde L, Trentin AG, Domingues MA, Alvarez-Silva M (2004). The effects of sub-chronic exposure of Wistar rats to the herbicide Glyphosate-Biocarb. Toxicol Lett.

[11] German Federal Agency BfR. The BfR has finalised its draft report for the re-evaluation of glyphosate. 2014. Available at http://www.bfr.bund.de/en/the_bfr_has_finalised_its_draft_report_for_the_re_evaluation_of_glyphosate-188632.html.

[12] Krüger M, Schrödl W, Neuhaus J, Shehata A (2013). Field investigations of glyphosate in urine of Danish dairy cows. J Environ Anal Toxicol.

[13] Thongprakaisang S, Thiantanawat A, Rangkadilok N, Suriyo T, Satayavivad J (2013). Glyphosate induces human breast cancer cells growth via estrogen receptors. Food Chem Toxicol.

[14] Paganelli A, Gnazzo V, Acosta H, Lopez SL, Carrasco AE (2010). Glyphosate-based herbicides produce teratogenic effects on vertebrates by impairing retinoic acid signaling. Chem Res Toxicol.

[15] Romano M, Romano R, Santos L, Wisniewski P, Campos D, de Souza P (2012). Glyphosate impairs male offspring reproductive development by disrupting gonadotropin expression. Arch Toxicol.

[16] Antoniou M, Habib MEM, Howard CV, Jennings RC, Leifert C, Nodari RO (2012). Teratogenic effects of glyphosate-based herbicides: divergence of regulatory decisions from scientific evidence. J Environ Anal Toxicol.

[17] Séralini G-E, Clair E, Mesnage R, Gress S, Defarge N, Malatesta M (2014). Republished study: long-term toxicity of a Roundup herbicide and a Roundup-tolerant genetically modified maize. Environ Sci Eur.

[18] Malatesta M, Caporaloni C, Gavaudan S, Rocchi M, Serafini S, Tiberi C (2002). Ultrastructural morphometrical and immunocytochemical analyses of hepatocyte nuclei from mice fed on genetically modified soybean. Cell Struct Funct.

[19] da Huang W, Sherman BT, Lempicki RA (2009). Systematic and integrative analysis of large gene lists using DAVID bioinformatics resources. Nat Protoc.

[20] Das RK, Banerjee S, Shapiro BH (2013). Extensive sex- and/or hormone-dependent expression of rat housekeeping genes. Endocr Res.

[21] Walsh LP, McCormick C, Martin C, Stocco DM (2000). Roundup inhibits steroidogenesis by disrupting steroidogenic acute regulatory (StAR) protein expression. Environ Health Perspect.

[22] Gasnier C, Dumont C, Benachour N, Clair E, Chagnon MC, Seralini GE (2009). Glyphosate-based herbicides are toxic and endocrine disruptors in human cell lines. Toxicology.

[23] Huang L, Heinloth AN, Zeng ZB, Paules RS, Bushel PR (2008). Genes related to apoptosis predict necrosis of the liver as a phenotype observed in rats exposed to a compendium of hepatotoxicants. BMC Genomics.

[24] Malatesta M, Boraldi F, Annovi G, Baldelli B, Battistelli S, Biggiogera M (2008). A long-term study on female mice fed on a genetically modified soybean: effects on liver ageing. Histochem Cell Biol.

[25] Malatesta M, Perdoni F, Santin G, Battistelli S, Muller S, Biggiogera M (2008). Hepatoma tissue culture (HTC) cells as a model for investigating the effects of low concentrations of herbicide on cell structure and function. Toxicol In Vitro.

[26] Peixoto F (2005). Comparative effects of the Roundup and glyphosate on mitochondrial oxidative phosphorylation. Chemosphere.

[27] Ugarte R (2014). Interaction between glyphosate and mitochondrial succinate dehydrogenase. Comp Theor Chem.

[28] Patil V, Canzoneri JC, Samatov TR, Luhrmann R, Oyelere AK (2012). Molecular architecture of zinc chelating small molecules that inhibit spliceosome assembly at an early stage. RNA.

[29] Lundager Madsen H, Christensen H, Gottlieb-Petersen C (1978). Stability constants of copper (II), zinc, manganese (II), calcium, and magnesium complexes of N-(phosphonomethyl) glycine (glyphosate). Acta Chem Scand A.

[30] Michel CI, Holley CL, Scruggs BS, Sidhu R, Brookheart RT, Listenberger LL (2011). Small nucleolar RNAs U32a, U33, and U35a are critical mediators of metabolic stress. Cell Metab.

[31] Hotamisligil GS (2010). Endoplasmic reticulum stress and the inflammatory basis of metabolic disease. Cell.

[32] Laplante M, Sabatini DM (2009). An emerging role of mTOR in lipid biosynthesis. Current biology Curr Biol.

[33] Schaffer JE (2003). Lipotoxicity: when tissues overeat. Curr Opin Lipidol.

[34] Uren Webster TM, Santos EM (2015). Global transcriptomic profiling demonstrates induction of oxidative stress and of compensatory cellular stress responses in brown trout exposed to glyphosate and Roundup. BMC Genomics.

[35] Moggs JG, Orphanides G (2001). Estrogen receptors: orchestrators of pleiotropic cellular responses. EMBO Rep.

[36] Antico Arciuch VG, Russo MA, Kang KS, Di Cristofano A (2013). Inhibition of AMPK and Krebs cycle gene expression drives metabolic remodeling of Pten-deficient preneoplastic thyroid cells. Cancer Res.

[37] Brown SJ, Stoilov P, Xing Y (2012). Chromatin and epigenetic regulation of pre-mRNA processing. Hum Mol Genet.

[38] Vandenberg L, Colborn T, Hayes T, Heindel J, Jacobs D, Lee D (2012). Hormones and endocrine-disrupting chemicals: low-dose effects and nonmonotonic dose responses. Endocr Rev.

[39] Davis TL, Whitesell JD, Cantlon JD, Clay CM, Nett TM (2011). Does a nonclassical signaling mechanism underlie an increase of estradiol-mediated gonadotropin-releasing hormone receptor binding in ovine pituitary cells?. Biol Reprod.

[40] Polyzos SA, Kountouras J, Deretzi G, Zavos C, Mantzoros CS (2012). The emerging role of endocrine disruptors in pathogenesis of insulin resistance: a concept implicating nonalcoholic fatty liver disease. Curr Mol Med.

[41] Hokanson R, Fudge R, Chowdhary R, Busbee D (2007). Alteration of estrogen-regulated gene expression in human cells induced by the agricultural and horticultural herbicide glyphosate. Hum Exp Toxicol.

[42] Mesnage R, Bernay B, Seralini GE (2013). Ethoxylated adjuvants of glyphosate-based herbicides are active principles of human cell toxicity. Toxicology.

[43] EPA. US: Basic information about glyphosate in drinking water. 2015. http://water.epa.gov/drink/contaminants/basicinformation/glyphosate.cfm (last access April).

[44] Peruzzo PJ, Porta AA, Ronco AE (2008). Levels of glyphosate in surface waters, sediments and soils associated with direct sowing soybean cultivation in north pampasic region of Argentina. Environ Pollut.

