# Erratum to: Transcriptome profile analysis reflects rat liver and kidney damage following chronic ultra-low dose Roundup exposure

**DOI:** 10.1186/s12940-017-0236-2

**Published:** 2017-03-23

**Authors:** Robin Mesnage, Matthew Arno, Manuela Costanzo, Manuela Malatesta, Gilles-Eric Séralini, Michael N. Antoniou

**Affiliations:** 1 Gene Expression and Therapy Group, Faculty of Life Sciences & Medicine, Department of Medical and Molecular Genetics, King’s College London, 8th Floor Tower Wing, Guy’s Hospital, Great Maze Pond, London, SE1 9RT UK; 2 Genomics Centre, King’s College London, Waterloo Campus, 150 Stamford Street, London, SE1 9NH UK; 3 Department of Neurological and Movement Sciences, University of Verona, Verona, 37134 Italy; 4 Institute of Biology, EA 2608 and Risk Pole, MRSH-CNRS, Esplanade de la Paix, University of Caen, Caen, 14032, Cedex France

## Erratum

The version of Fig. 1 that appears in our article [1] is incorrect: the wrong image was included as the upper ‘CONTROL’ panel of 1B. The correct figure is shown at the end of this Erratum. This error does not affect either the transcriptome data presented or the conclusions of the study.

**Fig. 1:**
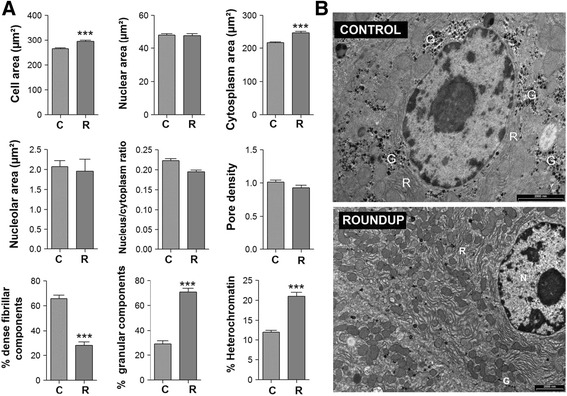
Alterations in hepatocyte nuclear architecture in female Roundup-treated rats suggests transcriptional disturbances. Liver from control (C) and Roundup (R) treated female rats were subjected to an ultrastructural electron microscopic analysis to investigate subcellular architecture. **a** Quantification of morphometric analysis of hepatocytes revealing alterations in subnuclear (heterochromatin, dense fibrillar, granular) compartments indicative of a reduced transcriptional status. Morphometric parameters are represented by their mean and their standard deviation. A two-tailed unpaired *t*-test was used as a standard test for statistical comparisons (***, *p <* 0.001). **b** Representative electron micrographs comparing hepatocytes from control (upper panel) and Roundup-treated (lower panel) rats showing a disruption of glycogen dispersion (G). N, nucleus; R rough endoplasmic reticulum
