# Supplementary material for: Transcriptome profile analysis reflects rat liver and kidney damage following chronic ultra-low dose Roundup exposure
Source: Environ Health. 2015 Aug 25;14:70. doi: 10.1186/s12940-015-0056-1 (PMC4549093; doi:10.1186/s12940-015-0056-1)
Supplement: Additional file 2: — PCA analysis fails to reveal a correlation between alterations in transcriptome profile and age at the time of death. Groups of 5 rats from the control and Roundup treatment categories were generated so that 5 animals with an earlier time of death (yellow spheres) were compared to 5 that were euthanised at a latter timepoint (purple spheres) in a PCA analysis. No significant differences in transcript cluster expression profiles were observed on this basis with samples from earlier and latter death timepoints being intermixed. For example, for livers, the transcript cluster expression profile of 10 rats with an earlier death timepoint (609 +/− 125 days) taken from controls (5 rats) and Roundup treated groups (5 rats) compared to the 10 animals with latter death (727 +/− 11 days) had a q-value of 0.99. (DOCX 42 kb) [file 12940_2015_56_MOESM2_ESM.docx]

**
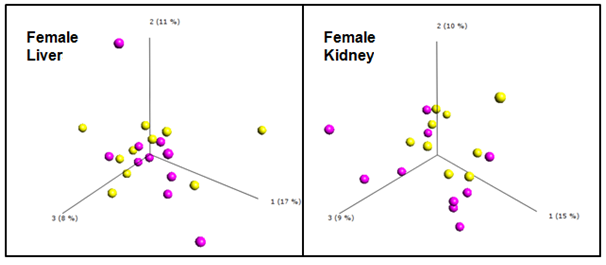
**

**Additional data 2. PCA analysis fails to reveal a correlation between alterations in transcriptome profile and age at the time of death.** Groups of 5 rats from the control and Roundup treatment categories were generated so that 5 animals with an earlier time of death (yellow spheres) were compared to 5 that were euthanised at a latter timepoint (purple spheres) in a PCA analysis**.** No significant differences in gene expression profiles were observed on this basis with samples from earlier and latter death timepoints being intermixed. For example, for livers, the gene expression profile of 10 rats with an earlier death timepoint (609 +/- 125 days) taken from controls (5 rats) and Roundup treated groups (5 rats) compared to the 10 animals with latter death (727 +/- 11 days) had a q-value of 0.99.
