# Supplementary material for: Transcriptome profile analysis reflects rat liver and kidney damage following chronic ultra-low dose Roundup exposure
Source: Environ Health. 2015 Aug 25;14:70. doi: 10.1186/s12940-015-0056-1 (PMC4549093; doi:10.1186/s12940-015-0056-1)
Supplement: Additional file 5: — Common transcript cluster disturbances in liver and kidneys of rats receiving 0.1 ppb Roundup in drinking water. ID is the Affymetrix transcript cluster identification number, FC indicates the fold change (upregulated transcript clusters highlighted in red; downregulated transcript clusters highlighted in green), P is the p-value (two-sided t-test) and q the false discovery rate (computed using the Benjamini-Hochberg method). The most significant differences (p <0.001) are highlighted in grey and were generated at the cut off values p <0.01 and FC >1.1 by using Qlucore Omics Explorer 3.0. Genes with fold change in expression >2 are highlighted in yellow. (DOCX 180 kb) [file 12940_2015_56_MOESM5_ESM.docx]

**Additional data 5. Common genes disturbances in liver and kidneys of rats receiving 0.1 ppb Roundup in drinking water.** Id is the Affymetrix transcript clusters identification number**,** FC is the fold change (upregulated genes in red fill, downregulated in green), P is the p-value (two-sided t-test) and Q the false discovery rate. The most significant differences (p<0.001) are indicated in grey fill. They were generated at the cut off values p < 0,01 and FC > 1.1 by using Qlucore Omics Explorer 3.0.

|  |  | Female Livers | | | Female Kidneys | | |
| --- | --- | --- | --- | --- | --- | --- | --- |
| ID | Gene Symbol | FC | P | Q | FC | P | Q |
| 17653485 | Abcc1 | 1.25 | 5.E-04 | 2.E-02 | 1.15 | 2.E-03 | 3.E-02 |
| 17857482 | Abcc10 | 1.1 | 6.E-03 | 6.E-02 | 1.13 | 8.E-03 | 7.E-02 |
| 17633027 | Abcc6 | 1.39 | 1.E-05 | 2.E-03 | 1.28 | 2.E-03 | 3.E-02 |
| 17729059 | Abce1 | -1.11 | 7.E-03 | 7.E-02 | -1.16 | 8.E-03 | 7.E-02 |
| 17752554 | Abcf1 | 1.13 | 2.E-03 | 3.E-02 | 1.17 | 4.E-04 | 1.E-02 |
| 17778174 | Abhd12 | -1.2 | 8.E-03 | 8.E-02 | -1.36 | 3.E-04 | 9.E-03 |
| 17756409 | Abhd16a | 1.18 | 5.E-04 | 2.E-02 | 1.14 | 6.E-04 | 1.E-02 |
| 17761275 | Abl1 | 1.2 | 1.E-03 | 3.E-02 | 1.15 | 2.E-04 | 7.E-03 |
| 17752115 | Acadm | -1.13 | 1.E-03 | 3.E-02 | -1.16 | 1.E-03 | 2.E-02 |
| 17717540 | Acbd5 /// LOC679565 | -1.34 | 2.E-07 | 2.E-04 | -1.3 | 5.E-05 | 3.E-03 |
| 17681065 | Acbd6 | 1.12 | 1.E-02 | 9.E-02 | 1.16 | 3.E-03 | 4.E-02 |
| 17702696 | Acin1 | 1.22 | 2.E-03 | 3.E-02 | 1.28 | 1.E-07 | 2.E-04 |
| 17631172 | Actn4 | 1.11 | 5.E-03 | 6.E-02 | -1.15 | 2.E-03 | 3.E-02 |
| 17835081 | Actr6 | -1.23 | 7.E-03 | 7.E-02 | -1.3 | 1.E-04 | 6.E-03 |
| 17823005 | Adam4 | 1.4 | 3.E-04 | 1.E-02 | 1.24 | 2.E-04 | 7.E-03 |
| 17823003 | Adam4l1 | 1.21 | 4.E-03 | 5.E-02 | 1.24 | 9.E-04 | 2.E-02 |
| 17833684 | Adat3 | 1.14 | 6.E-03 | 6.E-02 | 1.15 | 1.E-03 | 2.E-02 |
| 17840737 | Adcy6 | 1.42 | 5.E-06 | 1.E-03 | 1.25 | 3.E-04 | 1.E-02 |
| 17793478 | Aer61 | 1.19 | 2.E-03 | 3.E-02 | 1.11 | 2.E-03 | 3.E-02 |
| 17861475 | Agap1 | 1.24 | 4.E-06 | 1.E-03 | 1.21 | 5.E-03 | 5.E-02 |
| 17782108 | Ahcyl2 | -1.25 | 4.E-04 | 1.E-02 | -1.38 | 1.E-06 | 7.E-04 |
| 17879134 | Aifm1 | -1.21 | 1.E-04 | 7.E-03 | -1.17 | 4.E-03 | 4.E-02 |
| 17842142 | AJ240059 | 1.73 | 2.E-06 | 7.E-04 | 2.01 | 2.E-06 | 7.E-04 |
| 17802364 | Ak2 | -1.25 | 3.E-04 | 1.E-02 | -1.22 | 1.E-03 | 2.E-02 |
| 17880671 | Ak2 | -1.29 | 5.E-05 | 4.E-03 | -1.3 | 7.E-05 | 4.E-03 |
| 17834196 | Akap8 | 1.26 | 3.E-05 | 3.E-03 | 1.17 | 1.E-03 | 2.E-02 |
| 17616136 | Akt1s1 | 1.1 | 3.E-03 | 5.E-02 | 1.15 | 6.E-03 | 6.E-02 |
| 17726320 | Aldh7a1 | -1.18 | 6.E-03 | 7.E-02 | -1.28 | 4.E-03 | 5.E-02 |
| 17738555 | Alg5 | -1.64 | 2.E-05 | 2.E-03 | -1.79 | 4.E-05 | 3.E-03 |
| 17722057 | Ammecr1l | 1.16 | 8.E-03 | 8.E-02 | 1.17 | 4.E-04 | 1.E-02 |
| 17759855 | Anapc2 | 1.11 | 5.E-03 | 6.E-02 | 1.15 | 1.E-03 | 2.E-02 |
| 17709367 | Ankrd10 | 1.15 | 2.E-03 | 3.E-02 | 1.35 | 8.E-05 | 4.E-03 |
| 17688874 | Ankrd17 | 1.83 | 3.E-03 | 5.E-02 | 1.57 | 4.E-04 | 1.E-02 |
| 17688887 | Ankrd17 | 1.36 | 2.E-03 | 4.E-02 | 1.3 | 6.E-03 | 6.E-02 |
| 17651304 | Aoc2-ps1 /// Aoc3 /// G6pc /// Psme3 | 1.18 | 2.E-03 | 3.E-02 | 1.28 | 2.E-03 | 3.E-02 |
| 17751301 | Ap1ar | -1.23 | 2.E-03 | 3.E-02 | -1.14 | 9.E-03 | 8.E-02 |
| 17871350 | Ap1s1 | -1.36 | 3.E-06 | 9.E-04 | -1.16 | 5.E-03 | 5.E-02 |
| 17632575 | Ap2a1 | 1.11 | 6.E-03 | 6.E-02 | 1.16 | 6.E-05 | 4.E-03 |
| 17613670 | Ap2s1 | 1.16 | 7.E-03 | 7.E-02 | 1.21 | 3.E-04 | 1.E-02 |
| 17826255 | Ap3d1 | 1.3 | 1.E-06 | 6.E-04 | 1.33 | 5.E-05 | 3.E-03 |
| 17826245 | Ap3d1 | -1.21 | 5.E-03 | 6.E-02 | -1.43 | 1.E-03 | 2.E-02 |
| 17826260 | Ap3d1 | 1.48 | 2.E-06 | 7.E-04 | 1.31 | 1.E-03 | 2.E-02 |
| 17676127 | Ap3d1 | 1.12 | 2.E-03 | 3.E-02 | 1.22 | 1.E-05 | 2.E-03 |
| 17725680 | Apbb3 | 1.26 | 9.E-03 | 8.E-02 | 1.39 | 3.E-05 | 3.E-03 |
| 17764325 | Apip | -1.39 | 2.E-04 | 1.E-02 | -1.62 | 3.E-04 | 8.E-03 |
| 17856844 | Aplp2 | -1.16 | 2.E-03 | 3.E-02 | -1.31 | 5.E-07 | 4.E-04 |
| 17748623 | Apoa1bp | 1.25 | 1.E-02 | 9.E-02 | 1.35 | 3.E-04 | 1.E-02 |
| 17872826 | Apoo | -1.43 | 2.E-05 | 2.E-03 | -1.3 | 1.E-04 | 5.E-03 |
| 17872942 | Ar | -1.4 | 8.E-03 | 8.E-02 | -1.32 | 3.E-03 | 4.E-02 |
| 17618818 | Arap1 | 1.35 | 2.E-04 | 1.E-02 | 1.31 | 4.E-04 | 1.E-02 |
| 17851854 | Arcn1 | -1.14 | 3.E-03 | 4.E-02 | -1.2 | 2.E-04 | 8.E-03 |
| 17656675 | Arf1 | -1.39 | 1.E-03 | 3.E-02 | -1.32 | 1.E-03 | 2.E-02 |
| 17769672 | Arfgap1 | 1.22 | 2.E-04 | 8.E-03 | 1.21 | 3.E-04 | 1.E-02 |
| 17635813 | Arfip2 | 1.22 | 4.E-05 | 3.E-03 | 1.2 | 4.E-07 | 4.E-04 |
| 17709479 | Arglu1 | 1.23 | 2.E-04 | 1.E-02 | 1.15 | 3.E-03 | 4.E-02 |
| 17838812 | Arhgap39 | 1.2 | 3.E-03 | 5.E-02 | 1.21 | 3.E-04 | 1.E-02 |
| 17614323 | Arhgef1 | 1.15 | 3.E-04 | 1.E-02 | 1.22 | 4.E-03 | 4.E-02 |
| 17739600 | Arhgef11 | 1.32 | 4.E-05 | 3.E-03 | 1.18 | 9.E-04 | 2.E-02 |
| 17851581 | Arhgef12 | 1.23 | 4.E-04 | 1.E-02 | 1.12 | 2.E-06 | 8.E-04 |
| 17739869 | Arhgef2 | 1.17 | 1.E-02 | 9.E-02 | 1.23 | 2.E-04 | 8.E-03 |
| 17802663 | Arid1a | 1.35 | 1.E-03 | 3.E-02 | 1.17 | 5.E-05 | 3.E-03 |
| 17719811 | Arid4b | 1.22 | 1.E-03 | 3.E-02 | 1.11 | 7.E-03 | 7.E-02 |
| 17827443 | Arl1 | -1.2 | 3.E-03 | 4.E-02 | -1.36 | 3.E-06 | 1.E-03 |
| 17868850 | Arl13b | 1.21 | 4.E-03 | 5.E-02 | 1.2 | 3.E-03 | 4.E-02 |
| 17636544 | Arl6ip1 | -1.2 | 2.E-03 | 3.E-02 | -1.69 | 3.E-06 | 9.E-04 |
| 17680391 | Arl8a | 1.14 | 6.E-03 | 6.E-02 | 1.12 | 2.E-03 | 3.E-02 |
| 17785859 | Arl8b | -1.17 | 4.E-04 | 1.E-02 | -1.3 | 8.E-05 | 4.E-03 |
| 17746452 | Armc1 | 1.13 | 4.E-03 | 5.E-02 | -1.16 | 2.E-03 | 3.E-02 |
| 17621179 | Armc5 | 1.2 | 3.E-05 | 3.E-03 | 1.21 | 2.E-05 | 2.E-03 |
| 17770119 | Arrdc1 | 1.21 | 3.E-03 | 4.E-02 | 1.25 | 8.E-06 | 2.E-03 |
| 17840150 | Arsa | 1.26 | 5.E-04 | 2.E-02 | 1.22 | 6.E-04 | 1.E-02 |
| 17840672 | Asb8 | -1.3 | 3.E-06 | 9.E-04 | -1.13 | 4.E-03 | 5.E-02 |
| 17756620 | Atf6b | 1.19 | 4.E-04 | 1.E-02 | 1.16 | 8.E-05 | 4.E-03 |
| 17861286 | Atg16l1 | 1.18 | 1.E-04 | 6.E-03 | 1.1 | 1.E-03 | 2.E-02 |
| 17668877 | Atg3 | -1.24 | 4.E-03 | 5.E-02 | -1.22 | 5.E-04 | 1.E-02 |
| 17711114 | Atp13a1 | 1.18 | 1.E-04 | 6.E-03 | 1.2 | 9.E-05 | 5.E-03 |
| 17854761 | Atp1b3 | -1.2 | 5.E-04 | 2.E-02 | -1.2 | 1.E-03 | 2.E-02 |
| 17825486 | Atp5b | -1.1 | 3.E-03 | 5.E-02 | -1.12 | 2.E-03 | 3.E-02 |
| 17833806 | Atp5d | -1.31 | 5.E-04 | 2.E-02 | -1.32 | 5.E-06 | 1.E-03 |
| 17876025 | Atp6ap2 | -1.17 | 3.E-03 | 5.E-02 | -1.14 | 8.E-03 | 7.E-02 |
| 17727304 | Atp9b | 1.12 | 3.E-03 | 5.E-02 | 1.11 | 5.E-04 | 1.E-02 |
| 17831371 | Atxn10 | -1.21 | 6.E-05 | 4.E-03 | -1.19 | 3.E-04 | 8.E-03 |
| 17637131 | Atxn2l | 1.22 | 6.E-03 | 7.E-02 | 1.25 | 8.E-06 | 2.E-03 |
| 17714628 | Auh | -1.24 | 3.E-04 | 1.E-02 | -1.22 | 2.E-04 | 7.E-03 |
| 17629355 | Aurkc | 1.26 | 2.E-03 | 3.E-02 | 1.36 | 1.E-03 | 2.E-02 |
| 17794404 | B4galnt3 | -1.11 | 1.E-03 | 3.E-02 | -1.26 | 2.E-03 | 3.E-02 |
| 17718094 | B4galt7 | 1.14 | 3.E-03 | 5.E-02 | 1.25 | 3.E-04 | 9.E-03 |
| 17825502 | Baz2a | 1.31 | 8.E-04 | 2.E-02 | 1.18 | 1.E-04 | 5.E-03 |
| 17619895 | BC083805 /// LOC361635 /// LOC361635 /// LOC361635 | 1.39 | 9.E-03 | 8.E-02 | 1.47 | 5.E-04 | 1.E-02 |
| 17743353 | Bcl10 | -1.38 | 9.E-05 | 5.E-03 | -1.28 | 3.E-03 | 4.E-02 |
| 17677050 | Bcl7b | 1.13 | 4.E-03 | 5.E-02 | 1.24 | 4.E-04 | 1.E-02 |
| 17661660 | Becn1 | 1.54 | 6.E-04 | 2.E-02 | 1.32 | 9.E-03 | 8.E-02 |
| 17630270 | Bloc1s3 | -1.28 | 3.E-04 | 1.E-02 | -1.14 | 1.E-03 | 2.E-02 |
| 17704358 | Bmp1 | 1.3 | 4.E-06 | 1.E-03 | 1.31 | 7.E-04 | 2.E-02 |
| 17637979 | Bnip3 /// Bnip3-ps1 | -1.23 | 2.E-04 | 9.E-03 | -1.12 | 3.E-03 | 4.E-02 |
| 17839817 | Brd1 | 1.17 | 2.E-04 | 8.E-03 | 1.14 | 4.E-03 | 5.E-02 |
| 17834174 | Brd4 | 1.24 | 3.E-03 | 4.E-02 | 1.26 | 8.E-08 | 2.E-04 |
| 17725465 | Brd8 | 1.14 | 2.E-03 | 4.E-02 | 1.12 | 5.E-03 | 5.E-02 |
| 17745846 | Brix1 | -1.2 | 7.E-03 | 7.E-02 | -1.21 | 5.E-04 | 1.E-02 |
| 17834031 | Bsg | -1.29 | 9.E-05 | 5.E-03 | -1.42 | 4.E-07 | 4.E-04 |
| 17818455 | Bsr // lncRNA Db | -1.45 | 2.E-03 | 3.E-02 | 1.78 | 2.E-03 | 3.E-02 |
| 17757374 | Btbd9 | 1.11 | 1.E-02 | 8.E-02 | 1.18 | 2.E-03 | 3.E-02 |
| 17744599 | Btf3 | 1.16 | 3.E-04 | 1.E-02 | 1.11 | 1.E-03 | 2.E-02 |
| 17859500 | Bzw1 | -1.13 | 2.E-03 | 4.E-02 | -1.27 | 1.E-05 | 2.E-03 |
| 17618702 | C2cd3 | 1.17 | 4.E-03 | 5.E-02 | 1.15 | 9.E-03 | 8.E-02 |
| 17757850 | Cabin1 | 1.26 | 5.E-06 | 1.E-03 | 1.2 | 2.E-05 | 2.E-03 |
| 17685785 | Cacybp | -1.29 | 5.E-03 | 6.E-02 | -1.42 | 1.E-03 | 2.E-02 |
| 17820679 | Cad | 1.18 | 5.E-04 | 2.E-02 | 1.17 | 4.E-04 | 1.E-02 |
| 17704786 | Calm2 | 1.22 | 5.E-03 | 6.E-02 | 1.25 | 2.E-03 | 3.E-02 |
| 17658177 | Camta2 | 1.31 | 4.E-05 | 4.E-03 | 1.27 | 1.E-07 | 2.E-04 |
| 17615355 | Capns1 | -1.31 | 2.E-04 | 8.E-03 | -1.23 | 7.E-03 | 7.E-02 |
| 17781702 | Capza2 | -1.35 | 2.E-05 | 3.E-03 | -1.21 | 2.E-03 | 3.E-02 |
| 17713150 | Carkd | -1.11 | 8.E-03 | 7.E-02 | 1.19 | 6.E-05 | 4.E-03 |
| 17744705 | Cartpt | -1.17 | 9.E-03 | 8.E-02 | -1.19 | 4.E-04 | 1.E-02 |
| 17728806 | Cc2d1a | 1.18 | 3.E-03 | 4.E-02 | 1.26 | 3.E-04 | 9.E-03 |
| 17800765 | Cc2d1b | 1.21 | 9.E-05 | 5.E-03 | 1.15 | 1.E-04 | 6.E-03 |
| 17627915 | Ccdc127 | -1.36 | 2.E-05 | 2.E-03 | -1.36 | 9.E-05 | 4.E-03 |
| 17784746 | Ccdc142 | 1.29 | 2.E-04 | 1.E-02 | 1.33 | 1.E-05 | 2.E-03 |
| 17677674 | Ccdc62 | 1.23 | 6.E-03 | 7.E-02 | 1.3 | 7.E-07 | 5.E-04 |
| 17805008 | Ccnl2 | 1.25 | 7.E-04 | 2.E-02 | 1.32 | 2.E-05 | 2.E-03 |
| 17683808 | Ccnt2 | 1.22 | 8.E-04 | 2.E-02 | 1.19 | 6.E-06 | 1.E-03 |
| 17671822 | Ccz1 | 1.57 | 1.E-04 | 7.E-03 | 1.56 | 3.E-04 | 9.E-03 |
| 17775062 | Cd59 | -1.65 | 3.E-06 | 8.E-04 | -1.35 | 1.E-03 | 2.E-02 |
| 17774721 | Cd82 | 1.21 | 6.E-04 | 2.E-02 | 1.13 | 1.E-03 | 2.E-02 |
| 17725489 | Cdc23 | 1.22 | 9.E-07 | 4.E-04 | 1.13 | 1.E-03 | 2.E-02 |
| 17807928 | Cdc26 | -1.13 | 4.E-03 | 5.E-02 | -1.13 | 3.E-03 | 4.E-02 |
| 17834050 | Cdc34 | 1.21 | 5.E-06 | 1.E-03 | 1.28 | 7.E-06 | 1.E-03 |
| 17811686 | Cdc42 | -1.11 | 7.E-04 | 2.E-02 | -1.21 | 2.E-05 | 2.E-03 |
| 17830429 | Cdc42ep1 | 1.27 | 3.E-03 | 5.E-02 | 1.36 | 9.E-05 | 4.E-03 |
| 17650750 | Cdk12 | 1.33 | 5.E-06 | 1.E-03 | 1.2 | 7.E-07 | 5.E-04 |
| 17875742 | Cdk16 | 1.2 | 1.E-05 | 2.E-03 | 1.16 | 5.E-05 | 3.E-03 |
| 17771313 | Cdk9 | 1.11 | 9.E-03 | 8.E-02 | 1.15 | 7.E-04 | 1.E-02 |
| 17707836 | Cdkn2aip | -1.2 | 6.E-06 | 1.E-03 | -1.2 | 6.E-03 | 6.E-02 |
| 17645668 | Cdkn2aipnl | -1.3 | 4.E-04 | 1.E-02 | -1.35 | 3.E-04 | 1.E-02 |
| 17777458 | Cenpb | 1.19 | 7.E-05 | 5.E-03 | 1.21 | 2.E-03 | 3.E-02 |
| 17821922 | Cfl2 | -1.2 | 1.E-03 | 2.E-02 | -1.24 | 2.E-04 | 8.E-03 |
| 17790457 | Chchd3 | -1.16 | 3.E-03 | 4.E-02 | -1.13 | 3.E-03 | 4.E-02 |
| 17793265 | Chchd4 /// LOC100361898 /// LOC100364342 | -1.2 | 5.E-03 | 6.E-02 | -1.21 | 7.E-03 | 7.E-02 |
| 17778996 | Chd6 | 1.29 | 2.E-03 | 3.E-02 | 1.19 | 9.E-06 | 2.E-03 |
| 17797172 | Chd7 | 1.16 | 6.E-04 | 2.E-02 | 1.14 | 3.E-03 | 4.E-02 |
| 17788554 | Chpf2 /// Mir671 | 1.21 | 1.E-05 | 2.E-03 | 1.26 | 1.E-05 | 2.E-03 |
| 17670307 | Chrd | 1.27 | 2.E-05 | 2.E-03 | 1.29 | 2.E-03 | 3.E-02 |
| 17614356 | Cic | 1.34 | 3.E-06 | 9.E-04 | 1.22 | 1.E-06 | 7.E-04 |
| 17679606 | Clasp1 | 1.21 | 4.E-04 | 1.E-02 | 1.1 | 7.E-03 | 7.E-02 |
| 17741971 | Clcc1 | -1.21 | 4.E-04 | 1.E-02 | -1.22 | 3.E-05 | 2.E-03 |
| 17667724 | Cldn17 | -1.18 | 2.E-03 | 4.E-02 | 1.27 | 4.E-03 | 5.E-02 |
| 17653652 | Clec16a | 1.22 | 3.E-04 | 1.E-02 | 1.17 | 1.E-04 | 5.E-03 |
| 17881032 | Clint1 | 1.23 | 1.E-04 | 6.E-03 | 1.16 | 2.E-04 | 7.E-03 |
| 17740020 | Clk2 /// Mir3541 /// Scamp3 | 1.22 | 4.E-03 | 5.E-02 | 1.32 | 9.E-04 | 2.E-02 |
| 17852996 | Clk3 | 1.12 | 3.E-03 | 4.E-02 | 1.19 | 1.E-03 | 2.E-02 |
| 17854847 | Clstn2 | -1.17 | 7.E-03 | 7.E-02 | -1.36 | 4.E-03 | 4.E-02 |
| 17700825 | Clybl | -1.6 | 3.E-05 | 3.E-03 | -1.35 | 6.E-04 | 1.E-02 |
| 17788072 | Cmas | -1.18 | 1.E-05 | 2.E-03 | -1.21 | 2.E-03 | 3.E-02 |
| 17736958 | Cmbl | -1.19 | 2.E-05 | 2.E-03 | -1.45 | 9.E-04 | 2.E-02 |
| 17628303 | Cnksr3 | 1.31 | 6.E-03 | 7.E-02 | 1.2 | 6.E-03 | 6.E-02 |
| 17858430 | Cnnm3 | 1.23 | 5.E-05 | 4.E-03 | 1.26 | 6.E-04 | 1.E-02 |
| 17856174 | Cnot10 | 1.19 | 2.E-03 | 3.E-02 | 1.18 | 8.E-06 | 2.E-03 |
| 17708151 | Cnot7 | -1.59 | 5.E-07 | 4.E-04 | -1.42 | 5.E-04 | 1.E-02 |
| 17708152 | Cnot7 | -1.47 | 2.E-04 | 1.E-02 | -1.48 | 4.E-04 | 1.E-02 |
| 17708153 | Cnot7 | -1.4 | 6.E-03 | 6.E-02 | -1.42 | 1.E-03 | 2.E-02 |
| 17708148 | Cnot7 /// LOC100361786 | -1.32 | 5.E-04 | 2.E-02 | -1.28 | 3.E-03 | 4.E-02 |
| 17651251 | Cntd1 /// Vps25 /// Wnk4 | -1.2 | 2.E-03 | 4.E-02 | -1.2 | 3.E-04 | 1.E-02 |
| 17863930 | Coa5 | -1.18 | 2.E-03 | 3.E-02 | -1.19 | 7.E-04 | 2.E-02 |
| 17747513 | Commd2 | -1.22 | 4.E-05 | 3.E-03 | -1.17 | 8.E-03 | 7.E-02 |
| 17717352 | Commd3 | -1.3 | 8.E-04 | 2.E-02 | -1.23 | 7.E-03 | 7.E-02 |
| 17790362 | Copg2 | -1.17 | 2.E-03 | 4.E-02 | -1.19 | 1.E-03 | 2.E-02 |
| 17672365 | Cops6 | -1.2 | 2.E-06 | 6.E-04 | -1.19 | 3.E-05 | 3.E-03 |
| 17650204 | Cox11 | -1.33 | 2.E-04 | 9.E-03 | -1.1 | 9.E-03 | 8.E-02 |
| 17823007 | Cox16 /// Synj2bp | -1.52 | 7.E-10 | 7.E-06 | -1.29 | 4.E-03 | 4.E-02 |
| 17817929 | Cpsf2 | 1.19 | 2.E-03 | 3.E-02 | 1.14 | 4.E-03 | 5.E-02 |
| 17815004 | Cpsf3 | -1.18 | 5.E-05 | 4.E-03 | -1.16 | 7.E-03 | 7.E-02 |
| 17836009 | Cpsf6 | 1.13 | 3.E-03 | 5.E-02 | 1.16 | 5.E-04 | 1.E-02 |
| 17624032 | Cpsf7 | 1.23 | 2.E-04 | 1.E-02 | 1.29 | 1.E-06 | 6.E-04 |
| 17654645 | Cramp1l | 1.17 | 4.E-03 | 5.E-02 | 1.18 | 3.E-04 | 9.E-03 |
| 17703320 | Cryl1 | -1.44 | 5.E-03 | 6.E-02 | -1.45 | 3.E-03 | 4.E-02 |
| 17769116 | Cse1l | -1.21 | 5.E-03 | 6.E-02 | -1.2 | 5.E-03 | 6.E-02 |
| 17833659 | Csnk1g2 | 1.18 | 2.E-04 | 8.E-03 | 1.16 | 9.E-04 | 2.E-02 |
| 17805396 | Cspp1 | 1.17 | 3.E-03 | 4.E-02 | 1.25 | 1.E-04 | 5.E-03 |
| 17766820 | Csrp2bp | 1.17 | 7.E-04 | 2.E-02 | 1.23 | 2.E-03 | 3.E-02 |
| 17727129 | Ctif | 1.23 | 1.E-03 | 2.E-02 | 1.19 | 2.E-04 | 8.E-03 |
| 17699524 | Ctsb | -1.17 | 6.E-04 | 2.E-02 | -1.26 | 1.E-03 | 2.E-02 |
| 17846515 | Ctsh | -1.15 | 8.E-03 | 7.E-02 | -1.62 | 1.E-04 | 5.E-03 |
| 17857344 | Cul9 | 1.26 | 7.E-06 | 1.E-03 | 1.28 | 8.E-06 | 2.E-03 |
| 17745037 | Cwc27 | 1.17 | 1.E-03 | 2.E-02 | 1.17 | 3.E-04 | 1.E-02 |
| 17839557 | Cyb5r3 | -1.35 | 2.E-04 | 8.E-03 | -1.25 | 2.E-03 | 3.E-02 |
| 17625842 | Cyp2c23 | -1.1 | 3.E-03 | 5.E-02 | -1.65 | 2.E-04 | 6.E-03 |
| 17834292 | Cyp4f1 | -1.12 | 7.E-03 | 7.E-02 | -1.37 | 4.E-03 | 5.E-02 |
| 17720853 | Dclre1c | 1.25 | 3.E-04 | 1.E-02 | 1.26 | 8.E-05 | 4.E-03 |
| 17806624 | Dctn3 | -1.26 | 3.E-04 | 1.E-02 | -1.23 | 2.E-03 | 3.E-02 |
| 17881128 | Dctn3 /// Dctn3l1 | -1.37 | 5.E-05 | 4.E-03 | -1.24 | 5.E-03 | 5.E-02 |
| 17620359 | Dctn5 | -1.42 | 9.E-06 | 1.E-03 | -1.16 | 9.E-03 | 8.E-02 |
| 17830645 | Ddx17 | 1.31 | 4.E-04 | 1.E-02 | 1.17 | 3.E-05 | 3.E-03 |
| 17756321 | Ddx39b | 1.25 | 7.E-04 | 2.E-02 | 1.4 | 5.E-09 | 4.E-05 |
| 17638350 | Deaf1 | 1.17 | 8.E-04 | 2.E-02 | 1.27 | 9.E-07 | 6.E-04 |
| 17740327 | Dennd4b | 1.19 | 8.E-03 | 8.E-02 | 1.2 | 9.E-04 | 2.E-02 |
| 17808038 | Dfnb31 | 1.14 | 1.E-03 | 3.E-02 | 1.33 | 1.E-04 | 6.E-03 |
| 17774588 | Dgkz | 1.4 | 2.E-05 | 2.E-03 | 1.34 | 6.E-05 | 4.E-03 |
| 17855872 | Dhx30 | 1.19 | 1.E-04 | 7.E-03 | 1.2 | 8.E-05 | 4.E-03 |
| 17733392 | Dhx38 | 1.17 | 9.E-04 | 2.E-02 | 1.15 | 2.E-03 | 3.E-02 |
| 17819723 | Dhx57 | 1.16 | 7.E-04 | 2.E-02 | 1.12 | 1.E-03 | 2.E-02 |
| 17735614 | Dimt1 | -1.19 | 5.E-03 | 6.E-02 | -1.15 | 8.E-03 | 7.E-02 |
| 17754471 | Dip2a | 1.13 | 3.E-03 | 5.E-02 | 1.18 | 2.E-04 | 6.E-03 |
| 17832290 | Dip2b | 1.19 | 8.E-05 | 5.E-03 | 1.16 | 4.E-05 | 3.E-03 |
| 17696898 | Dlg5 | 1.23 | 1.E-03 | 2.E-02 | 1.15 | 3.E-03 | 4.E-02 |
| 17872611 | Dmd | -1.13 | 5.E-04 | 2.E-02 | 1.17 | 7.E-03 | 6.E-02 |
| 17814477 | Dnmt3a | 1.23 | 4.E-05 | 3.E-03 | 1.34 | 9.E-05 | 4.E-03 |
| 17865725 | Dnpep | 1.11 | 3.E-03 | 4.E-02 | 1.13 | 1.E-03 | 2.E-02 |
| 17833617 | Dot1l | 1.37 | 2.E-05 | 2.E-03 | 1.36 | 5.E-05 | 3.E-03 |
| 17867926 | Dpp9 | 1.31 | 3.E-05 | 3.E-03 | 1.2 | 3.E-04 | 9.E-03 |
| 17784717 | Dqx1 | 1.21 | 4.E-04 | 1.E-02 | 1.33 | 2.E-04 | 7.E-03 |
| 17695692 | Drg1 | -1.22 | 7.E-04 | 2.E-02 | -1.11 | 9.E-03 | 8.E-02 |
| 17836451 | Dtx3 | 1.3 | 9.E-03 | 8.E-02 | 1.36 | 1.E-05 | 2.E-03 |
| 17805044 | Dvl1 | 1.25 | 2.E-04 | 8.E-03 | 1.17 | 6.E-03 | 6.E-02 |
| 17670495 | Dvl3 | 1.18 | 3.E-04 | 1.E-02 | 1.13 | 2.E-05 | 2.E-03 |
| 17627881 | Echdc1 | -1.41 | 3.E-03 | 4.E-02 | -1.27 | 4.E-03 | 5.E-02 |
| 17800705 | Echdc2 | -1.28 | 9.E-06 | 1.E-03 | -1.17 | 1.E-02 | 8.E-02 |
| 17638151 | Echs1 | -1.13 | 9.E-03 | 8.E-02 | -1.16 | 7.E-03 | 7.E-02 |
| 17770091 | Ehmt1 /// LOC100909589 | 1.24 | 2.E-03 | 3.E-02 | 1.17 | 3.E-03 | 4.E-02 |
| 17756538 | Ehmt2 | 1.29 | 8.E-05 | 5.E-03 | 1.31 | 9.E-05 | 5.E-03 |
| 17675996 | Eif2ak1 | -1.35 | 8.E-05 | 5.E-03 | -1.35 | 8.E-05 | 4.E-03 |
| 17816934 | Eif2s1 | -1.53 | 2.E-06 | 7.E-04 | -1.51 | 2.E-05 | 2.E-03 |
| 17850167 | Eif3g | -1.27 | 3.E-04 | 1.E-02 | -1.16 | 3.E-03 | 4.E-02 |
| 17690964 | Eif4enif1 | 1.35 | 1.E-03 | 3.E-02 | 1.34 | 6.E-03 | 6.E-02 |
| 17670348 | Eif4g1 /// LOC100910289 | 1.27 | 1.E-07 | 1.E-04 | 1.11 | 1.E-03 | 2.E-02 |
| 17728952 | Elmod2 | -1.23 | 2.E-03 | 4.E-02 | -1.31 | 4.E-04 | 1.E-02 |
| 17850510 | Elof1 | -1.29 | 4.E-06 | 1.E-03 | 1.19 | 7.E-03 | 7.E-02 |
| 17721915 | Elp2 | -1.15 | 1.E-02 | 9.E-02 | -1.23 | 3.E-03 | 4.E-02 |
| 17611024 | Enpp1 | -1.11 | 6.E-03 | 6.E-02 | -1.28 | 2.E-03 | 3.E-02 |
| 17770744 | ENSRNOT00000006466 | -1.35 | 3.E-04 | 1.E-02 | -1.27 | 6.E-03 | 6.E-02 |
| 17791708 | ENSRNOT00000007437 | 1.61 | 2.E-04 | 8.E-03 | 1.41 | 8.E-04 | 2.E-02 |
| 17658722 | ENSRNOT00000010386 | -1.15 | 5.E-04 | 2.E-02 | -1.16 | 1.E-02 | 8.E-02 |
| 17843181 | ENSRNOT00000013370 | 1.3 | 8.E-04 | 2.E-02 | 1.18 | 1.E-03 | 2.E-02 |
| 17848555 | ENSRNOT00000017641 | -1.25 | 5.E-03 | 6.E-02 | -1.23 | 8.E-03 | 7.E-02 |
| 17713890 | ENSRNOT00000017930 //// GENSCAN00000031397 | 1.47 | 6.E-03 | 6.E-02 | 1.41 | 1.E-04 | 5.E-03 |
| 17857200 | ENSRNOT00000019177 | 1.23 | 1.E-04 | 7.E-03 | 1.29 | 1.E-05 | 2.E-03 |
| 17863242 | ENSRNOT00000030665 | 1.34 | 1.E-03 | 3.E-02 | 1.2 | 1.E-04 | 5.E-03 |
| 17862207 | ENSRNOT00000031814 | -1.29 | 3.E-04 | 1.E-02 | -1.19 | 4.E-03 | 5.E-02 |
| 17676156 | ENSRNOT00000032824 | -1.3 | 9.E-03 | 8.E-02 | -1.27 | 7.E-04 | 2.E-02 |
| 17682719 | ENSRNOT00000036078 | -1.12 | 2.E-03 | 4.E-02 | -1.13 | 2.E-03 | 3.E-02 |
| 17841349 | ENSRNOT00000039417 | -1.19 | 5.E-03 | 6.E-02 | -1.3 | 4.E-04 | 1.E-02 |
| 17845765 | ENSRNOT00000039420 / | 1.25 | 6.E-03 | 6.E-02 | 1.22 | 4.E-04 | 1.E-02 |
| 17775034 | ENSRNOT00000039555 | -1.15 | 5.E-03 | 6.E-02 | -1.23 | 8.E-03 | 7.E-02 |
| 17880349 | ENSRNOT00000039699 | -1.2 | 3.E-04 | 1.E-02 | -1.17 | 3.E-03 | 4.E-02 |
| 17632219 | ENSRNOT00000041760 /// ENSRNOT00000043099 | 1.83 | 3.E-03 | 5.E-02 | 1.31 | 7.E-03 | 7.E-02 |
| 17853499 | ENSRNOT00000041915 | -1.3 | 2.E-04 | 1.E-02 | -1.32 | 3.E-03 | 4.E-02 |
| 17854759 | ENSRNOT00000042885 | 1.23 | 4.E-03 | 5.E-02 | 1.32 | 8.E-04 | 2.E-02 |
| 17727541 | ENSRNOT00000043059 | -1.25 | 8.E-03 | 8.E-02 | -1.37 | 3.E-04 | 9.E-03 |
| 17848013 | ENSRNOT00000043345 | 1.41 | 7.E-03 | 7.E-02 | 1.33 | 3.E-06 | 1.E-03 |
| 17874140 | ENSRNOT00000043861 | -1.22 | 7.E-03 | 7.E-02 | -1.2 | 4.E-03 | 4.E-02 |
| 17704983 | ENSRNOT00000044402 | -1.38 | 5.E-03 | 6.E-02 | -1.47 | 3.E-03 | 4.E-02 |
| 17746992 | ENSRNOT00000044552 | 1.23 | 6.E-05 | 4.E-03 | -1.16 | 1.E-02 | 8.E-02 |
| 17813633 | ENSRNOT00000045349 | -1.4 | 4.E-03 | 5.E-02 | -1.31 | 7.E-04 | 2.E-02 |
| 17873514 | ENSRNOT00000049172 // chrX // 100 // 9 // 9 // 0 | -1.34 | 7.E-04 | 2.E-02 | -1.27 | 6.E-03 | 6.E-02 |
| 17711295 | ENSRNOT00000049456 | -1.21 | 7.E-03 | 7.E-02 | -1.37 | 1.E-04 | 5.E-03 |
| 17817227 | ENSRNOT00000051137 | 1.47 | 5.E-03 | 6.E-02 | 1.44 | 4.E-03 | 5.E-02 |
| 17872932 | ENSRNOT00000051856 | -1.24 | 7.E-03 | 7.E-02 | -1.21 | 3.E-03 | 4.E-02 |
| 17711509 | ENSRNOT00000051889 | -1.34 | 1.E-02 | 9.E-02 | -1.21 | 6.E-03 | 6.E-02 |
| 17739433 | ENSRNOT00000052051 | 1.23 | 4.E-03 | 5.E-02 | 1.24 | 6.E-06 | 1.E-03 |
| 17842138 | ENSRNOT00000052435 /// ENSRNOT00000053232 /// ENSRNOT00000052689 | 1.25 | 8.E-04 | 2.E-02 | 1.61 | 5.E-05 | 3.E-03 |
| 17781049 | ENSRNOT00000052459 | 1.32 | 9.E-03 | 8.E-02 | 1.29 | 1.E-03 | 2.E-02 |
| 17724287 | ENSRNOT00000052462 / | 2.46 | 2.E-05 | 2.E-03 | 1.59 | 2.E-03 | 3.E-02 |
| 17640315 | ENSRNOT00000052472 /// ENSRNOT00000054412 | 1.36 | 5.E-03 | 6.E-02 | 1.65 | 2.E-04 | 7.E-03 |
| 17640313 | ENSRNOT00000052472 /// ENSRNOT00000054412 | 1.36 | 5.E-03 | 6.E-02 | 1.65 | 2.E-04 | 7.E-03 |
| 17752109 | ENSRNOT00000052487 / | 1.84 | 2.E-04 | 1.E-02 | 4.24 | 2.E-05 | 2.E-03 |
| 17623685 | ENSRNOT00000052494 | 1.3 | 2.E-03 | 4.E-02 | 1.26 | 3.E-03 | 4.E-02 |
| 17673385 | ENSRNOT00000052527 | 1.18 | 2.E-03 | 4.E-02 | 1.25 | 2.E-03 | 3.E-02 |
| 17749668 | ENSRNOT00000052556 /// ENSRNOT00000053264 | 1.31 | 3.E-04 | 1.E-02 | 3.21 | 2.E-06 | 8.E-04 |
| 17710346 | ENSRNOT00000052571 | 1.4 | 1.E-05 | 2.E-03 | 1.94 | 2.E-06 | 8.E-04 |
| 17809650 | ENSRNOT00000052577 | 3.1 | 1.E-08 | 4.E-05 | 2.96 | 6.E-07 | 5.E-04 |
| 17803755 | ENSRNOT00000052625 | 1.59 | 3.E-03 | 5.E-02 | 2.15 | 1.E-04 | 5.E-03 |
| 17760349 | ENSRNOT00000052628 / | 2.03 | 3.E-07 | 3.E-04 | 1.86 | 1.E-05 | 2.E-03 |
| 17774100 | ENSRNOT00000052632 | 1.23 | 5.E-03 | 6.E-02 | 1.84 | 2.E-05 | 2.E-03 |
| 17677144 | ENSRNOT00000052642 | 1.38 | 2.E-03 | 3.E-02 | 1.76 | 3.E-05 | 3.E-03 |
| 17829858 | ENSRNOT00000052689 | 1.24 | 2.E-03 | 3.E-02 | 1.77 | 1.E-04 | 6.E-03 |
| 17756337 | ENSRNOT00000052790 | 1.81 | 2.E-07 | 2.E-04 | 1.88 | 4.E-05 | 3.E-03 |
| 17752878 | ENSRNOT00000052797 | 1.96 | 9.E-08 | 1.E-04 | 2.48 | 1.E-05 | 2.E-03 |
| 17623687 | ENSRNOT00000052816 / | 1.39 | 1.E-03 | 2.E-02 | 1.37 | 1.E-03 | 2.E-02 |
| 17756335 | ENSRNOT00000052837 / | 2.19 | 2.E-09 | 1.E-05 | 2.18 | 3.E-06 | 9.E-04 |
| 17619462 | ENSRNOT00000052871 | 1.96 | 5.E-06 | 1.E-03 | 3.29 | 4.E-06 | 1.E-03 |
| 17712909 | ENSRNOT00000052913 | 1.67 | 2.E-05 | 2.E-03 | 1.82 | 3.E-03 | 4.E-02 |
| 17866179 | ENSRNOT00000052932 | 1.86 | 1.E-05 | 2.E-03 | 2.07 | 5.E-09 | 4.E-05 |
| 17751221 | ENSRNOT00000052947 | 1.41 | 1.E-03 | 2.E-02 | 1.54 | 3.E-03 | 4.E-02 |
| 17670118 | ENSRNOT00000052956 | 1.73 | 5.E-04 | 1.E-02 | 3.23 | 9.E-05 | 5.E-03 |
| 17632725 | ENSRNOT00000052978 | 2.31 | 4.E-10 | 4.E-06 | 2.54 | 6.E-08 | 1.E-04 |
| 17768666 | ENSRNOT00000052983 | 1.86 | 3.E-05 | 3.E-03 | 2.21 | 3.E-04 | 9.E-03 |
| 17635186 | ENSRNOT00000052995 | 1.43 | 1.E-03 | 3.E-02 | 2.71 | 1.E-05 | 2.E-03 |
| 17842146 | ENSRNOT00000052997 | 1.34 | 3.E-03 | 5.E-02 | 1.19 | 2.E-03 | 3.E-02 |
| 17844984 | ENSRNOT00000053006 | 1.26 | 2.E-03 | 4.E-02 | 1.41 | 6.E-04 | 1.E-02 |
| 17854574 | ENSRNOT00000053015 | 3.73 | 3.E-06 | 9.E-04 | 2.14 | 6.E-03 | 6.E-02 |
| 17678148 | ENSRNOT00000053049 | 1.43 | 2.E-03 | 3.E-02 | 2.18 | 4.E-06 | 1.E-03 |
| 17855313 | ENSRNOT00000053061 | 1.65 | 2.E-06 | 7.E-04 | 2.24 | 6.E-05 | 4.E-03 |
| 17727949 | ENSRNOT00000053071 | 1.34 | 1.E-03 | 3.E-02 | 2.33 | 1.E-05 | 2.E-03 |
| 17879977 | ENSRNOT00000053135 | 1.59 | 6.E-06 | 1.E-03 | 1.66 | 1.E-04 | 6.E-03 |
| 17800100 | ENSRNOT00000053153 | -1.48 | 9.E-03 | 8.E-02 | -1.75 | 5.E-04 | 1.E-02 |
| 17773189 | ENSRNOT00000053156 | 1.24 | 2.E-03 | 3.E-02 | 2.32 | 3.E-05 | 2.E-03 |
| 17700156 | ENSRNOT00000053163 | 1.34 | 4.E-04 | 1.E-02 | 2.51 | 4.E-05 | 3.E-03 |
| 17821089 | ENSRNOT00000053179 | 1.23 | 2.E-03 | 3.E-02 | 1.5 | 5.E-04 | 1.E-02 |
| 17673387 | ENSRNOT00000053230 | 1.81 | 2.E-05 | 2.E-03 | 2.26 | 7.E-07 | 5.E-04 |
| 17646114 | ENSRNOT00000053264 /// ENSRNOT00000052556 | 1.26 | 3.E-03 | 4.E-02 | 3.24 | 5.E-06 | 1.E-03 |
| 17790119 | ENSRNOT00000053273 | 1.61 | 7.E-04 | 2.E-02 | 1.97 | 1.E-04 | 6.E-03 |
| 17669608 | ENSRNOT00000053274 /// ENSRNOT00000052821 /// ENSRNOT00000054087 /// ENSRNOT00000053196 /// ENSRNOT00000052502 | 1.23 | 2.E-04 | 8.E-03 | 1.39 | 7.E-04 | 2.E-02 |
| 17618324 | ENSRNOT00000053279 | 1.4 | 2.E-08 | 5.E-05 | 1.37 | 2.E-06 | 8.E-04 |
| 17696198 | ENSRNOT00000053290 | 1.18 | 3.E-03 | 5.E-02 | 1.72 | 8.E-06 | 2.E-03 |
| 17727794 | ENSRNOT00000053296 | 1.31 | 3.E-05 | 3.E-03 | 1.9 | 6.E-05 | 4.E-03 |
| 17801861 | ENSRNOT00000053301 /// ENSRNOT00000052632 | 1.37 | 3.E-06 | 9.E-04 | 1.62 | 2.E-07 | 3.E-04 |
| 17761412 | ENSRNOT00000053313 /// ENSRNOT00000052519 | 1.43 | 6.E-04 | 2.E-02 | 1.88 | 7.E-06 | 1.E-03 |
| 17761410 | ENSRNOT00000053313 /// ENSRNOT00000052519 | 1.43 | 6.E-04 | 2.E-02 | 1.88 | 7.E-06 | 1.E-03 |
| 17620907 | ENSRNOT00000053325 | 1.34 | 5.E-05 | 4.E-03 | 1.87 | 2.E-05 | 2.E-03 |
| 17632719 | ENSRNOT00000053364 | 1.81 | 4.E-06 | 1.E-03 | 1.77 | 2.E-08 | 9.E-05 |
| 17792333 | ENSRNOT00000053371 | 1.31 | 6.E-03 | 7.E-02 | 1.7 | 1.E-03 | 2.E-02 |
| 17648904 | ENSRNOT00000053376 | 1.75 | 3.E-04 | 1.E-02 | 1.51 | 3.E-03 | 4.E-02 |
| 17833511 | ENSRNOT00000053394 | 1.76 | 3.E-05 | 3.E-03 | 1.6 | 4.E-04 | 1.E-02 |
| 17632723 | ENSRNOT00000053402 | 1.93 | 1.E-08 | 4.E-05 | 2.09 | 2.E-07 | 2.E-04 |
| 17853660 | ENSRNOT00000053408 /// ENSRNOT00000052435 /// ENSRNOT00000052689 /// ENSRNOT00000053232 | 1.22 | 1.E-03 | 3.E-02 | 1.57 | 1.E-04 | 6.E-03 |
| 17681823 | ENSRNOT00000053447 | 1.28 | 7.E-03 | 7.E-02 | 1.47 | 1.E-03 | 2.E-02 |
| 17842140 | ENSRNOT00000053450 | 3.14 | 3.E-06 | 9.E-04 | 2.6 | 4.E-05 | 3.E-03 |
| 17611073 | ENSRNOT00000053476 | 1.64 | 2.E-04 | 9.E-03 | 1.96 | 4.E-05 | 3.E-03 |
| 17778971 | ENSRNOT00000053479 | 1.27 | 2.E-04 | 9.E-03 | 1.42 | 4.E-05 | 3.E-03 |
| 17809654 | ENSRNOT00000053497 | 1.86 | 2.E-07 | 2.E-04 | 1.71 | 4.E-06 | 1.E-03 |
| 17823453 | ENSRNOT00000053532 | 1.32 | 3.E-06 | 9.E-04 | 1.58 | 2.E-04 | 6.E-03 |
| 17801863 | ENSRNOT00000053542 | 1.37 | 1.E-05 | 2.E-03 | 1.38 | 2.E-04 | 6.E-03 |
| 17742139 | ENSRNOT00000053816 /// ENSRNOT00000052616 | 1.47 | 3.E-05 | 3.E-03 | 2.28 | 5.E-06 | 1.E-03 |
| 17842134 | ENSRNOT00000053816 /// ENSRNOT00000052616 | 1.47 | 3.E-05 | 3.E-03 | 2.28 | 5.E-06 | 1.E-03 |
| 17825181 | ENSRNOT00000053840 /// ENSRNOT00000053021 /// ENSRNOT00000052861 | 1.68 | 5.E-04 | 2.E-02 | 2.19 | 1.E-05 | 2.E-03 |
| 17793995 | ENSRNOT00000053840 /// ENSRNOT00000053021 /// ENSRNOT00000052861 | 1.68 | 5.E-04 | 2.E-02 | 2.19 | 1.E-05 | 2.E-03 |
| 17752880 | ENSRNOT00000053874 | 2.8 | 3.E-09 | 1.E-05 | 2.25 | 5.E-06 | 1.E-03 |
| 17696164 | ENSRNOT00000053878 /// GENSCAN00000016610 | 1.66 | 4.E-05 | 4.E-03 | 2.38 | 6.E-06 | 1.E-03 |
| 17842144 | ENSRNOT00000053906 /// ENSRNOT00000052842 | 1.44 | 1.E-03 | 2.E-02 | 1.48 | 2.E-06 | 8.E-04 |
| 17683259 | ENSRNOT00000053909 | 2.06 | 2.E-04 | 8.E-03 | 2.57 | 8.E-05 | 4.E-03 |
| 17670114 | ENSRNOT00000053919 | 1.31 | 2.E-06 | 8.E-04 | 1.46 | 6.E-06 | 1.E-03 |
| 17810979 | ENSRNOT00000053920 /// ENSRNOT00000053389 | 1.42 | 9.E-07 | 5.E-04 | 1.37 | 5.E-06 | 1.E-03 |
| 17810981 | ENSRNOT00000053920 /// ENSRNOT00000053389 / | 1.42 | 9.E-07 | 5.E-04 | 1.37 | 5.E-06 | 1.E-03 |
| 17612139 | ENSRNOT00000053951 | 1.86 | 8.E-05 | 5.E-03 | 2.16 | 6.E-06 | 1.E-03 |
| 17848894 | ENSRNOT00000053968 | 1.3 | 1.E-03 | 2.E-02 | 2 | 5.E-05 | 3.E-03 |
| 17670112 | ENSRNOT00000053987 | 1.76 | 3.E-03 | 4.E-02 | 1.72 | 3.E-04 | 9.E-03 |
| 17722258 | ENSRNOT00000053992 | 1.47 | 5.E-05 | 4.E-03 | 1.66 | 4.E-04 | 1.E-02 |
| 17844990 | ENSRNOT00000054058 | 1.31 | 8.E-03 | 8.E-02 | 1.35 | 8.E-03 | 7.E-02 |
| 17646713 | ENSRNOT00000054060 | 1.89 | 7.E-06 | 1.E-03 | 1.92 | 4.E-03 | 5.E-02 |
| 17635184 | ENSRNOT00000054102 | 1.39 | 9.E-04 | 2.E-02 | 1.87 | 8.E-05 | 4.E-03 |
| 17685824 | ENSRNOT00000054127 | 1.24 | 5.E-04 | 2.E-02 | 1.29 | 3.E-04 | 8.E-03 |
| 17623683 | ENSRNOT00000054147 | 1.76 | 8.E-09 | 3.E-05 | 1.6 | 4.E-08 | 1.E-04 |
| 17646715 | ENSRNOT00000054149 | 1.64 | 5.E-03 | 6.E-02 | 1.66 | 6.E-03 | 6.E-02 |
| 17848896 | ENSRNOT00000054178 | 1.73 | 2.E-03 | 3.E-02 | 2.1 | 3.E-03 | 4.E-02 |
| 17632721 | ENSRNOT00000054185 | 3.2 | 3.E-08 | 6.E-05 | 3.34 | 8.E-07 | 5.E-04 |
| 17772381 | ENSRNOT00000054229 | 1.5 | 6.E-05 | 5.E-03 | 1.74 | 1.E-05 | 2.E-03 |
| 17760347 | ENSRNOT00000054299 | 2.33 | 2.E-05 | 2.E-03 | 2.36 | 6.E-04 | 1.E-02 |
| 17778969 | ENSRNOT00000054339 | 1.31 | 9.E-04 | 2.E-02 | 2.09 | 6.E-05 | 4.E-03 |
| 17622590 | ENSRNOT00000054860 | -1.25 | 2.E-03 | 3.E-02 | -1.16 | 2.E-04 | 7.E-03 |
| 17620865 | ENSRNOT00000054997 | 1.35 | 2.E-03 | 3.E-02 | 1.18 | 6.E-05 | 4.E-03 |
| 17650243 | ENSRNOT00000055650 | -1.38 | 3.E-03 | 5.E-02 | -1.46 | 4.E-04 | 1.E-02 |
| 17703784 | ENSRNOT00000056869 | 1.3 | 2.E-03 | 3.E-02 | 1.16 | 7.E-03 | 6.E-02 |
| 17759330 | ENSRNOT00000057118 | 1.41 | 1.E-06 | 6.E-04 | 1.21 | 3.E-03 | 4.E-02 |
| 17792498 | ENSRNOT00000057854 | -1.48 | 2.E-03 | 3.E-02 | -1.49 | 4.E-05 | 3.E-03 |
| 17763253 | ENSRNOT00000057875 | -1.3 | 6.E-04 | 2.E-02 | -1.33 | 3.E-03 | 4.E-02 |
| 17656364 | ENSRNOT00000058624 | -1.15 | 5.E-03 | 6.E-02 | -1.15 | 9.E-03 | 8.E-02 |
| 17690282 | ENSRNOT00000058847 | 1.35 | 8.E-04 | 2.E-02 | 1.46 | 6.E-04 | 1.E-02 |
| 17707346 | ENSRNOT00000059111 | 1.47 | 7.E-04 | 2.E-02 | 1.51 | 9.E-05 | 5.E-03 |
| 17694441 | ENSRNOT00000060076 | 1.27 | 6.E-03 | 7.E-02 | 1.34 | 1.E-04 | 6.E-03 |
| 17722738 | ENSRNOT00000060466 | 1.3 | 2.E-03 | 4.E-02 | 1.49 | 1.E-05 | 2.E-03 |
| 17849529 | ENSRNOT00000061711 | -1.33 | 2.E-04 | 8.E-03 | -1.38 | 1.E-03 | 2.E-02 |
| 17870607 | ENSRNOT00000062247 //// ENSRNOT00000062351 | 1.3 | 5.E-03 | 6.E-02 | 2.01 | 5.E-06 | 1.E-03 |
| 17644349 | ENSRNOT00000062287 | 1.36 | 4.E-03 | 5.E-02 | 1.52 | 1.E-03 | 2.E-02 |
| 17732408 | ENSRNOT00000062293 / | 1.53 | 1.E-03 | 3.E-02 | 1.94 | 2.E-05 | 2.E-03 |
| 17674844 | ENSRNOT00000062303 | 1.63 | 2.E-07 | 2.E-04 | 2.04 | 7.E-06 | 1.E-03 |
| 17678750 | ENSRNOT00000062315 | 1.47 | 5.E-03 | 6.E-02 | 1.83 | 3.E-03 | 4.E-02 |
| 17692574 | ENSRNOT00000062380 /// ENSRNOT00000062573 /// GENSCAN00000015332 | 1.19 | 1.E-03 | 3.E-02 | 1.79 | 6.E-05 | 4.E-03 |
| 17644254 | ENSRNOT00000062394 | 2.9 | 3.E-06 | 9.E-04 | 2.43 | 7.E-04 | 2.E-02 |
| 17623677 | ENSRNOT00000062413 | 1.74 | 3.E-04 | 1.E-02 | 1.91 | 9.E-05 | 5.E-03 |
| 17646717 | ENSRNOT00000062416 | 1.32 | 2.E-04 | 9.E-03 | 1.28 | 4.E-03 | 5.E-02 |
| 17777845 | ENSRNOT00000062422 | 1.47 | 5.E-03 | 6.E-02 | 3.24 | 4.E-05 | 3.E-03 |
| 17693562 | ENSRNOT00000062423 | -1.22 | 8.E-03 | 8.E-02 | -1.25 | 8.E-03 | 7.E-02 |
| 17770731 | ENSRNOT00000062467 | 1.35 | 9.E-03 | 8.E-02 | 1.64 | 8.E-04 | 2.E-02 |
| 17838859 | ENSRNOT00000062493 | 1.83 | 8.E-05 | 5.E-03 | 1.48 | 3.E-03 | 4.E-02 |
| 17670381 | ENSRNOT00000062511 | 1.47 | 1.E-06 | 6.E-04 | 1.7 | 4.E-06 | 1.E-03 |
| 17839270 | ENSRNOT00000062514 | 2.26 | 7.E-04 | 2.E-02 | 2.19 | 1.E-04 | 5.E-03 |
| 17757127 | ENSRNOT00000062515 | 1.23 | 4.E-03 | 5.E-02 | 1.5 | 4.E-05 | 3.E-03 |
| 17709862 | ENSRNOT00000062538 | 1.55 | 7.E-04 | 2.E-02 | 1.51 | 3.E-04 | 9.E-03 |
| 17844687 | ENSRNOT00000062543 /// ENSRNOT00000062290 | 1.57 | 7.E-05 | 5.E-03 | 1.41 | 6.E-04 | 1.E-02 |
| 17842132 | ENSRNOT00000062543 /// ENSRNOT00000062290 | 1.57 | 7.E-05 | 5.E-03 | 1.41 | 6.E-04 | 1.E-02 |
| 17865227 | ENSRNOT00000062545 // chr9 // 100 // 8 // 8 // 0 | 1.31 | 7.E-04 | 2.E-02 | 1.28 | 3.E-03 | 4.E-02 |
| 17760451 | ENSRNOT00000062555 /// ENSRNOT00000062886 /// ENSRNOT00000062788 /// ENSRNOT00000062641 /// ENSRNOT00000062315 | 1.4 | 1.E-03 | 3.E-02 | 1.7 | 3.E-04 | 9.E-03 |
| 17729996 | ENSRNOT00000062577 | 2.04 | 8.E-07 | 4.E-04 | 2.35 | 6.E-06 | 1.E-03 |
| 17835135 | ENSRNOT00000062604 | 1.89 | 9.E-05 | 5.E-03 | 2.75 | 8.E-05 | 4.E-03 |
| 17709866 | ENSRNOT00000062615 | 1.93 | 3.E-04 | 1.E-02 | 1.54 | 4.E-04 | 1.E-02 |
| 17748426 | ENSRNOT00000062664 | 2.56 | 2.E-10 | 4.E-06 | 3.62 | 1.E-06 | 6.E-04 |
| 17656760 | ENSRNOT00000062706 | 1.29 | 9.E-03 | 8.E-02 | 1.24 | 4.E-03 | 5.E-02 |
| 17842354 | ENSRNOT00000062710 | 1.52 | 5.E-06 | 1.E-03 | 1.69 | 1.E-05 | 2.E-03 |
| 17764101 | ENSRNOT00000062716 | 1.19 | 2.E-03 | 3.E-02 | 1.46 | 6.E-06 | 1.E-03 |
| 17831906 | ENSRNOT00000062780 /// ENSRNOT00000062850 | 1.33 | 5.E-04 | 2.E-02 | 2.61 | 4.E-05 | 3.E-03 |
| 17653408 | ENSRNOT00000062788 | 1.42 | 6.E-03 | 7.E-02 | 1.56 | 5.E-03 | 6.E-02 |
| 17625804 | ENSRNOT00000062845 | 1.24 | 7.E-04 | 2.E-02 | 1.48 | 1.E-04 | 5.E-03 |
| 17619464 | ENSRNOT00000062850 /// ENSRNOT00000062780 | 1.32 | 4.E-04 | 1.E-02 | 2.41 | 9.E-05 | 5.E-03 |
| 17876552 | ENSRNOT00000062861 /// ENSRNOT00000062375 | 1.18 | 6.E-03 | 7.E-02 | 1.61 | 4.E-03 | 4.E-02 |
| 17748424 | ENSRNOT00000062872 | 1.66 | 3.E-04 | 1.E-02 | 2.76 | 1.E-06 | 6.E-04 |
| 17864112 | ENSRNOT00000062900 | 1.63 | 1.E-05 | 2.E-03 | 3.4 | 3.E-06 | 1.E-03 |
| 17683592 | ENSRNOT00000063170 / | 1.84 | 2.E-08 | 4.E-05 | 2.06 | 1.E-06 | 6.E-04 |
| 17611071 | ENSRNOT00000063250 | 1.29 | 6.E-05 | 4.E-03 | 2.34 | 1.E-04 | 5.E-03 |
| 17792222 | ENSRNOT00000063334 | 1.31 | 1.E-03 | 3.E-02 | 1.5 | 2.E-03 | 3.E-02 |
| 17733519 | ENSRNOT00000063400 | 1.35 | 5.E-04 | 2.E-02 | 1.55 | 1.E-03 | 2.E-02 |
| 17710187 | ENSRNOT00000063469 | 1.26 | 5.E-04 | 2.E-02 | 1.63 | 9.E-05 | 5.E-03 |
| 17858083 | ENSRNOT00000063476 | -1.16 | 5.E-03 | 6.E-02 | -1.24 | 4.E-03 | 5.E-02 |
| 17711586 | ENSRNOT00000063600 | 1.49 | 3.E-04 | 1.E-02 | 1.37 | 2.E-03 | 3.E-02 |
| 17654595 | ENSRNOT00000064652 /// ENSRNOT00000066463 /// ENSRNOT00000030458 | 1.11 | 1.E-03 | 3.E-02 | 1.23 | 1.E-04 | 6.E-03 |
| 17676159 | ENSRNOT00000065337 | -1.14 | 5.E-03 | 6.E-02 | 1.23 | 1.E-04 | 6.E-03 |
| 17746063 | ENSRNOT00000066196 | 1.35 | 2.E-03 | 3.E-02 | 1.16 | 4.E-03 | 4.E-02 |
| 17665255 | ENSRNOT00000066257 | 1.31 | 7.E-04 | 2.E-02 | 1.31 | 5.E-03 | 6.E-02 |
| 17787115 | ENSRNOT00000068836 | 1.28 | 6.E-03 | 6.E-02 | 1.53 | 2.E-06 | 8.E-04 |
| 17787116 | ENSRNOT00000068836 | 1.28 | 6.E-03 | 6.E-02 | 1.53 | 2.E-06 | 8.E-04 |
| 17750632 | ENSRNOT00000068860 | 1.32 | 2.E-03 | 3.E-02 | 1.65 | 6.E-05 | 4.E-03 |
| 17794983 | ENSRNOT00000068887 | 2.2 | 1.E-04 | 6.E-03 | 2.12 | 1.E-04 | 6.E-03 |
| 17859651 | ENSRNOT00000068889 | 2.29 | 4.E-06 | 1.E-03 | 2.02 | 2.E-04 | 7.E-03 |
| 17806577 | ENSRNOT00000068892 | 1.58 | 5.E-03 | 6.E-02 | 1.23 | 9.E-03 | 7.E-02 |
| 17657789 | ENSRNOT00000068893 | 1.4 | 1.E-04 | 7.E-03 | 1.36 | 7.E-04 | 2.E-02 |
| 17636301 | ENSRNOT00000068939 | 1.54 | 2.E-03 | 3.E-02 | 1.62 | 2.E-05 | 2.E-03 |
| 17636302 | ENSRNOT00000068939 | 1.54 | 2.E-03 | 3.E-02 | 1.62 | 2.E-05 | 2.E-03 |
| 17718539 | ENSRNOT00000068958 | 1.76 | 3.E-03 | 5.E-02 | 5.32 | 9.E-07 | 5.E-04 |
| 17805604 | ENSRNOT00000068959 /// GENSCAN00000007421 | 1.72 | 7.E-05 | 5.E-03 | 2.08 | 1.E-06 | 7.E-04 |
| 17842356 | ENSRNOT00000069057 | 1.64 | 2.E-04 | 9.E-03 | 1.97 | 2.E-05 | 2.E-03 |
| 17735096 | ENSRNOT00000069073 ENSRNOT00000069043 | 1.48 | 1.E-03 | 3.E-02 | 2.45 | 1.E-03 | 2.E-02 |
| 17830698 | ENSRNOT00000069073/// ENSRNOT00000069043 | 1.48 | 1.E-03 | 3.E-02 | 2.45 | 1.E-03 | 2.E-02 |
| 17815867 | ENSRNOT00000069075 /// ENSRNOT00000070428 /// ENSRNOT00000054151 /// ENSRNOT00000069935 /// ENSRNOT00000063720 /// ENSRNOT00000070649 | 1.2 | 5.E-03 | 6.E-02 | 1.44 | 2.E-04 | 7.E-03 |
| 17670116 | ENSRNOT00000069086 | 1.38 | 2.E-03 | 3.E-02 | 2.31 | 1.E-05 | 2.E-03 |
| 17799105 | ENSRNOT00000069155 | 1.12 | 8.E-03 | 8.E-02 | 1.27 | 1.E-02 | 8.E-02 |
| 17768890 | ENSRNOT00000069239 | 1.3 | 7.E-04 | 2.E-02 | 1.23 | 4.E-03 | 5.E-02 |
| 17866888 | ENSRNOT00000069314 | 1.39 | 9.E-04 | 2.E-02 | 1.37 | 3.E-06 | 9.E-04 |
| 17872257 | ENSRNOT00000069381 | 1.56 | 8.E-05 | 5.E-03 | 1.69 | 3.E-05 | 2.E-03 |
| 17856249 | ENSRNOT00000069418 | 1.95 | 1.E-05 | 2.E-03 | 3.33 | 4.E-08 | 1.E-04 |
| 17627239 | ENSRNOT00000069426 | -1.23 | 1.E-03 | 3.E-02 | -1.33 | 2.E-03 | 3.E-02 |
| 17735293 | ENSRNOT00000069461 | 1.25 | 6.E-04 | 2.E-02 | 1.64 | 9.E-04 | 2.E-02 |
| 17821249 | ENSRNOT00000069563 | 1.69 | 1.E-04 | 7.E-03 | 1.99 | 2.E-03 | 3.E-02 |
| 17739910 | ENSRNOT00000069575 | 1.26 | 4.E-03 | 5.E-02 | 2.46 | 5.E-05 | 3.E-03 |
| 17684875 | ENSRNOT00000069668 | 1.51 | 1.E-03 | 2.E-02 | 1.53 | 5.E-04 | 1.E-02 |
| 17861310 | ENSRNOT00000069802 | 1.39 | 2.E-03 | 4.E-02 | 2.81 | 3.E-06 | 1.E-03 |
| 17696594 | ENSRNOT00000069825 | -1.16 | 3.E-03 | 5.E-02 | -1.24 | 2.E-04 | 8.E-03 |
| 17718898 | ENSRNOT00000069966 / | -1.21 | 6.E-03 | 6.E-02 | -1.19 | 8.E-03 | 7.E-02 |
| 17839737 | ENSRNOT00000070006 | 1.32 | 7.E-03 | 7.E-02 | 1.75 | 2.E-04 | 7.E-03 |
| 17734839 | ENSRNOT00000070038 | 1.74 | 2.E-05 | 2.E-03 | 1.36 | 2.E-03 | 3.E-02 |
| 17746435 | ENSRNOT00000070111 | -1.2 | 4.E-03 | 5.E-02 | 1.69 | 4.E-04 | 1.E-02 |
| 17760351 | ENSRNOT00000070202 | 2.13 | 7.E-05 | 5.E-03 | 2.27 | 1.E-05 | 2.E-03 |
| 17859645 | ENSRNOT00000070213 | 1.51 | 8.E-06 | 1.E-03 | 1.84 | 1.E-06 | 7.E-04 |
| 17861308 | ENSRNOT00000070293 | 1.46 | 1.E-03 | 3.E-02 | 1.86 | 3.E-06 | 9.E-04 |
| 17789258 | ENSRNOT00000070340 | 1.59 | 8.E-03 | 8.E-02 | 1.44 | 1.E-04 | 5.E-03 |
| 17711829 | ENSRNOT00000070404 | 1.8 | 1.E-03 | 3.E-02 | 1.72 | 3.E-04 | 9.E-03 |
| 17677810 | ENSRNOT00000070416 | 1.13 | 9.E-03 | 8.E-02 | 1.47 | 4.E-03 | 4.E-02 |
| 17849826 | ENSRNOT00000070428 /// ENSRNOT00000069075 // /// ENSRNOT00000054151 /// ENSRNOT00000069935 /// ENSRNOT00000063720/// ENSRNOT00000070649/// ENSRNOT00000052762 | 1.2 | 3.E-03 | 5.E-02 | 1.39 | 5.E-04 | 1.E-02 |
| 17657791 | ENSRNOT00000070525 | 1.34 | 1.E-05 | 2.E-03 | 1.57 | 3.E-05 | 2.E-03 |
| 17748743 | ENSRNOT00000070597 | -1.21 | 2.E-03 | 3.E-02 | -1.27 | 6.E-05 | 4.E-03 |
| 17837642 | ENSRNOT00000070609 | 1.47 | 1.E-03 | 2.E-02 | 1.33 | 6.E-03 | 6.E-02 |
| 17631208 | ENSRNOT00000070629 | 1.35 | 2.E-03 | 4.E-02 | 1.45 | 6.E-05 | 4.E-03 |
| 17674785 | Ep400 | 1.25 | 2.E-04 | 9.E-03 | 1.11 | 2.E-03 | 3.E-02 |
| 17791050 | Epha1 | 1.28 | 7.E-04 | 2.E-02 | 1.26 | 2.E-03 | 3.E-02 |
| 17629512 | Epn1 | 1.19 | 5.E-05 | 4.E-03 | 1.13 | 7.E-03 | 7.E-02 |
| 17614084 | Ercc2 /// Mir343 | 1.17 | 1.E-03 | 3.E-02 | 1.2 | 1.E-03 | 2.E-02 |
| 17796512 | Ergic2 | -1.17 | 6.E-03 | 7.E-02 | -1.12 | 9.E-03 | 8.E-02 |
| 17717437 | Etl4 | 1.24 | 7.E-03 | 7.E-02 | 1.26 | 2.E-04 | 6.E-03 |
| 17807015 | Exosc3 | -1.48 | 2.E-06 | 7.E-04 | -1.24 | 4.E-03 | 5.E-02 |
| 17826308 | Fam108a1 | 1.22 | 1.E-04 | 7.E-03 | 1.14 | 7.E-03 | 7.E-02 |
| 17777260 | Fam113a | 1.23 | 1.E-03 | 3.E-02 | 1.31 | 7.E-06 | 1.E-03 |
| 17791078 | Fam115e | -1.15 | 7.E-03 | 7.E-02 | -1.24 | 4.E-03 | 5.E-02 |
| 17704391 | Fam160b2 | 1.26 | 4.E-04 | 1.E-02 | 1.27 | 4.E-05 | 3.E-03 |
| 17695426 | Fam193a | 1.25 | 2.E-03 | 3.E-02 | 1.21 | 5.E-05 | 3.E-03 |
| 17808471 | Fam29a | 1.3 | 8.E-04 | 2.E-02 | 1.54 | 3.E-04 | 9.E-03 |
| 17626858 | Fam45a | -1.19 | 5.E-05 | 4.E-03 | -1.14 | 1.E-02 | 8.E-02 |
| 17760991 | Fam73b | 1.18 | 5.E-05 | 4.E-03 | 1.21 | 1.E-07 | 2.E-04 |
| 17775721 | Fam82a2 | -1.2 | 2.E-03 | 3.E-02 | -1.23 | 2.E-05 | 2.E-03 |
| 17845309 | Fam96a | -1.47 | 2.E-05 | 2.E-03 | -1.31 | 1.E-03 | 2.E-02 |
| 17780567 | Fastk | 1.2 | 1.E-04 | 7.E-03 | 1.16 | 2.E-04 | 7.E-03 |
| 17663412 | Fbf1 | 1.17 | 1.E-03 | 3.E-02 | 1.21 | 6.E-05 | 4.E-03 |
| 17674863 | Fbrsl1 | 1.18 | 3.E-05 | 3.E-03 | 1.21 | 3.E-04 | 1.E-02 |
| 17838648 | Fbxl6 | 1.14 | 2.E-03 | 3.E-02 | 1.2 | 2.E-03 | 3.E-02 |
| 17716808 | Fbxo18 | 1.22 | 9.E-05 | 5.E-03 | 1.15 | 3.E-04 | 1.E-02 |
| 17642421 | Fbxw4 | 1.16 | 6.E-05 | 5.E-03 | 1.13 | 1.E-03 | 2.E-02 |
| 17817439 | Fcf1 | 1.24 | 3.E-04 | 1.E-02 | 1.16 | 2.E-03 | 3.E-02 |
| 17817440 | Fcf1 | 1.29 | 2.E-03 | 4.E-02 | 1.26 | 1.E-04 | 5.E-03 |
| 17850243 | Fdx1l | -1.3 | 6.E-05 | 4.E-03 | -1.19 | 8.E-03 | 7.E-02 |
| 17862009 | Fert2 | 1.16 | 3.E-03 | 5.E-02 | 1.12 | 2.E-03 | 3.E-02 |
| 17666512 | Fgf12 | -1.16 | 4.E-03 | 5.E-02 | -1.28 | 8.E-04 | 2.E-02 |
| 17692413 | Fgfrl1 | -1.22 | 3.E-04 | 1.E-02 | -1.31 | 1.E-04 | 5.E-03 |
| 17686924 | Fh | -1.18 | 1.E-03 | 3.E-02 | -1.18 | 7.E-04 | 1.E-02 |
| 17733052 | Fhod1 | 1.18 | 4.E-04 | 1.E-02 | 1.28 | 2.E-04 | 7.E-03 |
| 17710929 | Fkbp8 | 1.16 | 4.E-03 | 5.E-02 | 1.26 | 6.E-05 | 4.E-03 |
| 17654204 | Flywch1 | 1.22 | 8.E-07 | 4.E-04 | 1.14 | 4.E-03 | 5.E-02 |
| 17686035 | Fmo1 | -1.63 | 5.E-03 | 6.E-02 | -1.24 | 5.E-03 | 5.E-02 |
| 17763815 | Fnbp4 | 1.18 | 3.E-03 | 5.E-02 | 1.24 | 4.E-05 | 3.E-03 |
| 17786921 | Foxj2 | 1.2 | 2.E-03 | 3.E-02 | 1.22 | 2.E-07 | 3.E-04 |
| 17857172 | Foxp4 | 1.17 | 5.E-04 | 2.E-02 | 1.18 | 3.E-05 | 3.E-03 |
| 17851805 | Foxr1 | -1.16 | 8.E-03 | 8.E-02 | -1.19 | 3.E-04 | 9.E-03 |
| 17715852 | FQ212013 | 1.28 | 4.E-03 | 5.E-02 | 1.43 | 3.E-03 | 4.E-02 |
| 17645602 | FQ223275 | 1.31 | 4.E-03 | 5.E-02 | 1.31 | 3.E-03 | 4.E-02 |
| 17820580 | FQ225254 | -1.27 | 9.E-04 | 2.E-02 | -1.27 | 7.E-05 | 4.E-03 |
| 17801430 | FQ225254 | 1.52 | 6.E-03 | 6.E-02 | 1.99 | 1.E-04 | 6.E-03 |
| 17647364 | FQ225891 | 2.91 | 5.E-05 | 4.E-03 | 4.45 | 1.E-05 | 2.E-03 |
| 17787247 | FQ228444 | 1.27 | 3.E-03 | 4.E-02 | 1.79 | 4.E-04 | 1.E-02 |
| 17792147 | FQ231069 | -1.13 | 2.E-03 | 4.E-02 | -1.15 | 3.E-03 | 4.E-02 |
| 17732657 | FQ231763 | 1.16 | 3.E-04 | 1.E-02 | 1.15 | 1.E-02 | 8.E-02 |
| 17755996 | Gabbr1 | 1.36 | 7.E-06 | 1.E-03 | 1.5 | 1.E-08 | 7.E-05 |
| 17813587 | Galm | -1.11 | 3.E-03 | 4.E-02 | -1.29 | 4.E-04 | 1.E-02 |
| 17771520 | Gapvd1 | 1.3 | 7.E-06 | 1.E-03 | 1.21 | 2.E-06 | 8.E-04 |
| 17681307 | Gas5 | 1.91 | 2.E-06 | 8.E-04 | 2.15 | 2.E-04 | 6.E-03 |
| 17681319 | Gas5 | 1.49 | 4.E-08 | 6.E-05 | 1.42 | 2.E-07 | 3.E-04 |
| 17681321 | Gas5 | 1.36 | 7.E-03 | 7.E-02 | 1.42 | 2.E-03 | 3.E-02 |
| 17681327 | Gas5 | 2.01 | 4.E-06 | 9.E-04 | 1.83 | 1.E-04 | 5.E-03 |
| 17681329 | Gas5 | 1.86 | 5.E-07 | 4.E-04 | 2.17 | 7.E-06 | 1.E-03 |
| 17681331 | Gas5 | 1.98 | 5.E-07 | 4.E-04 | 1.76 | 2.E-03 | 3.E-02 |
| 17707282 | Gatad2a | 1.26 | 1.E-04 | 6.E-03 | 1.14 | 3.E-03 | 4.E-02 |
| 17678570 | Gcn1l1 /// LOC679955 | 1.24 | 1.E-05 | 2.E-03 | 1.12 | 8.E-04 | 2.E-02 |
| 17733845 | Gcsh /// RGD1563099 | -1.3 | 3.E-04 | 1.E-02 | -1.27 | 9.E-03 | 8.E-02 |
| 17780393 | GENSCAN00000001213 | 1.23 | 7.E-04 | 2.E-02 | 1.11 | 3.E-03 | 4.E-02 |
| 17830198 | GENSCAN00000003123 | 1.16 | 7.E-05 | 5.E-03 | 1.23 | 4.E-04 | 1.E-02 |
| 17871338 | GENSCAN00000011876 | 1.2 | 9.E-03 | 8.E-02 | 1.56 | 2.E-04 | 8.E-03 |
| 17778973 | GENSCAN00000019086 | 1.94 | 2.E-05 | 2.E-03 | 2.38 | 4.E-05 | 3.E-03 |
| 17737927 | GENSCAN00000023786 | 1.13 | 2.E-03 | 3.E-02 | -1.21 | 5.E-03 | 5.E-02 |
| 17668580 | GENSCAN00000025600 | 1.17 | 5.E-04 | 2.E-02 | 1.17 | 1.E-03 | 2.E-02 |
| 17668822 | GENSCAN00000027063 /// ENSRNOT00000030033 | 1.15 | 3.E-03 | 4.E-02 | 1.19 | 8.E-05 | 4.E-03 |
| 17775226 | GENSCAN00000029337 | -1.28 | 2.E-03 | 3.E-02 | -1.2 | 2.E-03 | 3.E-02 |
| 17735632 | GENSCAN00000040261 | -1.2 | 1.E-03 | 3.E-02 | -1.2 | 2.E-03 | 3.E-02 |
| 17718537 | GENSCAN00000047636 | 1.61 | 7.E-04 | 2.E-02 | 1.41 | 6.E-03 | 6.E-02 |
| 17710503 | Ghitm | -1.17 | 6.E-03 | 6.E-02 | -1.29 | 2.E-04 | 6.E-03 |
| 17650476 | Gip | -1.23 | 5.E-03 | 6.E-02 | -1.39 | 1.E-03 | 2.E-02 |
| 17659090 | Git1 | 1.24 | 9.E-04 | 2.E-02 | 1.15 | 5.E-03 | 5.E-02 |
| 17674531 | Git2 | 1.16 | 2.E-03 | 3.E-02 | 1.16 | 3.E-04 | 1.E-02 |
| 17647798 | Gltpd2 | -1.26 | 3.E-04 | 1.E-02 | -1.33 | 2.E-03 | 3.E-02 |
| 17738875 | Gmps | -1.29 | 4.E-06 | 9.E-04 | -1.13 | 5.E-03 | 5.E-02 |
| 17662724 | Gna13 | -1.22 | 7.E-04 | 2.E-02 | -1.17 | 4.E-03 | 5.E-02 |
| 17876808 | Gnl3l | -1.19 | 9.E-04 | 2.E-02 | -1.19 | 7.E-03 | 6.E-02 |
| 17654700 | Gnptg | -1.19 | 4.E-03 | 5.E-02 | -1.17 | 7.E-04 | 2.E-02 |
| 17771877 | Golga1 | 1.15 | 5.E-03 | 6.E-02 | 1.13 | 5.E-04 | 1.E-02 |
| 17708822 | Golga7 | -1.22 | 8.E-05 | 5.E-03 | -1.14 | 4.E-03 | 5.E-02 |
| 17736596 | Golph3 | -1.22 | 3.E-03 | 4.E-02 | -1.16 | 4.E-04 | 1.E-02 |
| 17873547 | Gpr174 | -1.14 | 4.E-04 | 1.E-02 | -1.17 | 6.E-03 | 6.E-02 |
| 17795863 | Gpr19 | -1.17 | 4.E-03 | 5.E-02 | -1.16 | 1.E-03 | 2.E-02 |
| 17631853 | Gramd1a | 1.16 | 3.E-03 | 4.E-02 | 1.35 | 1.E-05 | 2.E-03 |
| 17718114 | Grk6 | 1.12 | 3.E-03 | 5.E-02 | 1.12 | 1.E-02 | 8.E-02 |
| 17730641 | Gse1 | 1.13 | 1.E-04 | 6.E-03 | 1.3 | 5.E-07 | 4.E-04 |
| 17750404 | Gstm7 | -1.38 | 6.E-04 | 2.E-02 | -1.34 | 4.E-03 | 4.E-02 |
| 17636995 | Gtf3c1 | 1.23 | 2.E-06 | 7.E-04 | 1.18 | 4.E-05 | 3.E-03 |
| 17714337 | H2afy | -1.17 | 6.E-04 | 2.E-02 | -1.13 | 8.E-05 | 4.E-03 |
| 17840560 | Hdac7 | 1.14 | 5.E-03 | 6.E-02 | 1.32 | 3.E-05 | 2.E-03 |
| 17627423 | Heca | 1.11 | 5.E-03 | 6.E-02 | 1.13 | 8.E-05 | 4.E-03 |
| 17688395 | Helq | 1.15 | 5.E-03 | 6.E-02 | 1.19 | 3.E-03 | 4.E-02 |
| 17616998 | Herc2 | 1.22 | 3.E-06 | 9.E-04 | 1.12 | 1.E-03 | 2.E-02 |
| 17744528 | Hexb | -1.27 | 1.E-02 | 9.E-02 | -1.53 | 5.E-04 | 1.E-02 |
| 17653390 | Hexdc | 1.25 | 5.E-04 | 2.E-02 | 1.28 | 3.E-05 | 3.E-03 |
| 17612880 | Hiat1 | -1.32 | 2.E-04 | 1.E-02 | -1.32 | 7.E-04 | 2.E-02 |
| 17791415 | Hibadh | -1.31 | 9.E-06 | 1.E-03 | -1.32 | 3.E-04 | 1.E-02 |
| 17816622 | Hif1a | -1.13 | 3.E-03 | 4.E-02 | -1.17 | 1.E-03 | 2.E-02 |
| 17677640 | Hip1r | 1.33 | 6.E-04 | 2.E-02 | 1.25 | 5.E-04 | 1.E-02 |
| 17668049 | Hlcs | 1.32 | 3.E-06 | 9.E-04 | 1.17 | 2.E-04 | 7.E-03 |
| 17655949 | Hnrnpab | -1.1 | 1.E-02 | 9.E-02 | -1.16 | 1.E-04 | 5.E-03 |
| 17786480 | Hnrnpf | 1.22 | 8.E-04 | 2.E-02 | 1.22 | 4.E-07 | 4.E-04 |
| 17614978 | Hnrnpl | 1.25 | 1.E-04 | 7.E-03 | 1.16 | 2.E-03 | 3.E-02 |
| 17631828 | Hpn | 1.15 | 6.E-03 | 7.E-02 | 1.29 | 2.E-03 | 3.E-02 |
| 17638287 | Hras | 1.14 | 3.E-03 | 5.E-02 | 1.25 | 2.E-03 | 3.E-02 |
| 17670136 | Hrg /// LOC681544 | -1.13 | 2.E-03 | 3.E-02 | -3.01 | 4.E-03 | 5.E-02 |
| 17864662 | Hspd1 | -1.22 | 3.E-05 | 3.E-03 | -1.29 | 2.E-04 | 6.E-03 |
| 17880291 | Hspd1 | -1.34 | 5.E-09 | 2.E-05 | -1.31 | 9.E-05 | 5.E-03 |
| 17874668 | Htatsf1 | 1.18 | 5.E-03 | 6.E-02 | 1.19 | 8.E-03 | 7.E-02 |
| 17695298 | Htt | 1.21 | 2.E-05 | 2.E-03 | 1.13 | 7.E-04 | 2.E-02 |
| 17781881 | Hyal4 | -1.18 | 1.E-03 | 3.E-02 | -1.17 | 4.E-03 | 4.E-02 |
| 17777229 | Idh3B | -1.26 | 1.E-05 | 2.E-03 | -1.24 | 2.E-05 | 2.E-03 |
| 17879816 | Idh3g | -1.28 | 3.E-08 | 6.E-05 | -1.36 | 4.E-06 | 1.E-03 |
| 17787266 | Iffo1 | 1.21 | 2.E-04 | 1.E-02 | 1.14 | 7.E-03 | 7.E-02 |
| 17786329 | Ift122 | 1.16 | 4.E-03 | 5.E-02 | 1.2 | 4.E-04 | 1.E-02 |
| 17669062 | Igsf11 | -1.21 | 6.E-03 | 7.E-02 | -1.55 | 1.E-03 | 2.E-02 |
| 17637725 | Ikzf5 | -1.18 | 2.E-04 | 8.E-03 | -1.2 | 6.E-04 | 1.E-02 |
| 17859094 | Inpp1 | 1.24 | 6.E-04 | 2.E-02 | 1.15 | 2.E-03 | 3.E-02 |
| 17635371 | Inppl1 | 1.26 | 5.E-03 | 6.E-02 | 1.18 | 2.E-03 | 3.E-02 |
| 17702940 | Ipo4 | 1.19 | 7.E-04 | 2.E-02 | 1.2 | 1.E-03 | 2.E-02 |
| 17871901 | Iqsec2 | 1.2 | 1.E-03 | 2.E-02 | 1.16 | 8.E-04 | 2.E-02 |
| 17613889 | Irf2bp1 | 1.25 | 5.E-04 | 2.E-02 | 1.24 | 4.E-03 | 4.E-02 |
| 17823403 | Irf2bpl | 1.35 | 9.E-04 | 2.E-02 | 1.36 | 6.E-04 | 1.E-02 |
| 17613026 | Isoc2b | 1.31 | 2.E-04 | 1.E-02 | 1.48 | 2.E-08 | 1.E-04 |
| 17704535 | Itm2b | -1.15 | 2.E-05 | 2.E-03 | -1.16 | 1.E-04 | 6.E-03 |
| 17765139 | Itpka | -1.26 | 5.E-04 | 2.E-02 | -1.19 | 1.E-02 | 8.E-02 |
| 17682563 | Itpkb | 1.14 | 7.E-04 | 2.E-02 | 1.22 | 7.E-03 | 7.E-02 |
| 17796396 | Itpr2 | 1.17 | 7.E-04 | 2.E-02 | 1.24 | 5.E-04 | 1.E-02 |
| 17869726 | Itsn2 | 1.25 | 3.E-05 | 3.E-03 | 1.17 | 5.E-06 | 1.E-03 |
| 17764953 | Ivd | -1.38 | 3.E-05 | 3.E-03 | -1.18 | 6.E-03 | 6.E-02 |
| 17806794 | J05014 | 1.55 | 1.E-04 | 7.E-03 | 2.03 | 7.E-05 | 4.E-03 |
| 17706947 | Jak3 | 1.32 | 3.E-03 | 5.E-02 | 1.23 | 6.E-04 | 1.E-02 |
| 17765239 | Jmjd7 /// Pla2g4b | 1.23 | 7.E-04 | 2.E-02 | 1.26 | 4.E-04 | 1.E-02 |
| 17616061 | Josd2 | 1.27 | 1.E-04 | 6.E-03 | 1.15 | 3.E-03 | 4.E-02 |
| 17863778 | Kansl3 | 1.19 | 4.E-04 | 1.E-02 | 1.14 | 2.E-03 | 3.E-02 |
| 17661451 | Kat2a | 1.27 | 1.E-04 | 6.E-03 | 1.19 | 7.E-04 | 2.E-02 |
| 17639238 | Kdm2a | 1.24 | 6.E-04 | 2.E-02 | 1.1 | 6.E-04 | 1.E-02 |
| 17657603 | Kdm6b | 1.25 | 3.E-03 | 5.E-02 | 1.15 | 1.E-03 | 2.E-02 |
| 17867737 | Khsrp | 1.19 | 8.E-05 | 5.E-03 | 1.11 | 1.E-03 | 2.E-02 |
| 17699564 | Kif13b | 1.24 | 4.E-05 | 4.E-03 | 1.15 | 5.E-03 | 5.E-02 |
| 17716122 | Kif5b | -1.15 | 2.E-04 | 1.E-02 | -1.23 | 5.E-06 | 1.E-03 |
| 17819003 | Klc1 | 1.25 | 9.E-05 | 5.E-03 | 1.23 | 6.E-04 | 1.E-02 |
| 17660954 | Krt26 | -1.17 | 6.E-03 | 6.E-02 | -1.25 | 7.E-03 | 6.E-02 |
| 17841255 | Krt80 | -1.31 | 5.E-04 | 2.E-02 | 1.22 | 3.E-03 | 4.E-02 |
| 17740082 | Krtcap2 | 1.26 | 1.E-04 | 6.E-03 | 1.24 | 5.E-05 | 3.E-03 |
| 17822365 | L2hgdh | -1.29 | 3.E-06 | 9.E-04 | -1.19 | 9.E-03 | 8.E-02 |
| 17830940 | L3mbtl2 | 1.15 | 3.E-03 | 4.E-02 | 1.12 | 1.E-03 | 2.E-02 |
| 17634873 | l7Rn6 | -1.24 | 6.E-04 | 2.E-02 | -1.22 | 7.E-03 | 7.E-02 |
| 17828842 | Laptm4b | -1.31 | 9.E-03 | 8.E-02 | -1.31 | 3.E-03 | 4.E-02 |
| 17738156 | Larp1b | 1.14 | 6.E-03 | 7.E-02 | 1.16 | 5.E-03 | 6.E-02 |
| 17770299 | Lcn12 | 1.24 | 1.E-03 | 2.E-02 | 1.23 | 9.E-03 | 7.E-02 |
| 17642510 | Ldb1 | 1.11 | 5.E-03 | 6.E-02 | 1.16 | 4.E-04 | 1.E-02 |
| 17811433 | Ldlrap1 | 1.25 | 2.E-04 | 9.E-03 | 1.14 | 8.E-04 | 2.E-02 |
| 17629709 | Leng8 | 1.24 | 7.E-03 | 7.E-02 | 1.41 | 2.E-07 | 3.E-04 |
| 17801592 | Lepre1 | 1.11 | 9.E-03 | 8.E-02 | 1.24 | 1.E-04 | 6.E-03 |
| 17708295 | Leprotl1 | 1.26 | 8.E-04 | 2.E-02 | 1.15 | 2.E-03 | 3.E-02 |
| 17694459 | Lias | -1.45 | 5.E-08 | 7.E-05 | -1.36 | 6.E-04 | 1.E-02 |
| 17769779 | Lime1 /// Zgpat | 1.22 | 6.E-04 | 2.E-02 | 1.27 | 1.E-06 | 7.E-04 |
| 17769790 | Lime1 /// Zgpat | 1.19 | 4.E-03 | 5.E-02 | 1.29 | 1.E-04 | 5.E-03 |
| 17758967 | Lims1 | -1.11 | 7.E-03 | 7.E-02 | -1.32 | 3.E-04 | 9.E-03 |
| 17652728 | Llgl2 | 1.29 | 6.E-03 | 6.E-02 | 1.15 | 3.E-03 | 4.E-02 |
| 17780296 | Lmbr1 | -1.34 | 1.E-06 | 5.E-04 | -1.48 | 1.E-05 | 2.E-03 |
| 17830165 | LOC100125385 | 1.17 | 5.E-05 | 4.E-03 | 1.17 | 7.E-04 | 2.E-02 |
| 17726352 | LOC100174910 | 1.2 | 7.E-03 | 7.E-02 | 1.28 | 9.E-04 | 2.E-02 |
| 17701156 | LOC100359425 /// LOC100363159 /// LOC100363894 /// LOC100364581 /// LOC679527 /// LOC679839 /// LOC679895 /// LOC680400 /// LOC680597 /// LOC680653 /// LOC680694 /// RGD1559628 /// RGD1560592 /// RGD1561956 /// RGD1562612 /// RGD1564372 | -1.35 | 1.E-03 | 3.E-02 | -1.48 | 1.E-03 | 2.E-02 |
| 17880495 | LOC100359468 | -1.45 | 2.E-04 | 9.E-03 | -1.62 | 8.E-05 | 4.E-03 |
| 17693886 | LOC100359719 /// LOC100361661 /// LOC100361880 /// LOC100362239 /// LOC100363220 /// LOC100363244 /// LOC100363391 /// LOC100363901 /// LOC100364361 /// LOC100364645 /// Rpl39 | 1.16 | 6.E-04 | 2.E-02 | 1.16 | 5.E-03 | 5.E-02 |
| 17701751 | LOC100359719 /// LOC100361661 /// LOC100361880 /// LOC100362239 /// LOC100363220 /// LOC100363244 /// LOC100363391 /// LOC100363901 /// LOC100364361 /// LOC100364645 /// Rpl39 | 1.19 | 2.E-04 | 9.E-03 | 1.24 | 3.E-04 | 9.E-03 |
| 17724936 | LOC100359719 /// LOC100361661 /// LOC100361880 /// LOC100362239 /// LOC100363220 /// LOC100363244 /// LOC100363391 /// LOC100363901 /// LOC100364361 /// LOC100364645 /// Rpl39 | 1.16 | 6.E-04 | 2.E-02 | 1.16 | 4.E-03 | 5.E-02 |
| 17827731 | LOC100359719 /// LOC100361661 /// LOC100361880 /// LOC100362239 /// LOC100363220 /// LOC100363244 /// LOC100363391 /// LOC100363901 /// LOC100364361 /// LOC100364645 /// Rpl39 | 1.19 | 2.E-04 | 9.E-03 | 1.22 | 9.E-04 | 2.E-02 |
| 17870602 | LOC100359719 /// LOC100361661 /// LOC100361880 /// LOC100362239 /// LOC100363220 /// LOC100363244 /// LOC100363391 /// LOC100363901 /// LOC100364361 /// LOC100364645 /// Rpl39 | 1.17 | 3.E-04 | 1.E-02 | 1.17 | 4.E-03 | 4.E-02 |
| 17877129 | LOC100359719 /// LOC100361661 /// LOC100361880 /// LOC100362239 /// LOC100363220 /// LOC100363244 /// LOC100363391 /// LOC100363901 /// LOC100364361 /// LOC100364645 /// Rpl39 | 1.19 | 2.E-04 | 9.E-03 | 1.24 | 3.E-04 | 9.E-03 |
| 17792908 | LOC100360057 | 1.81 | 2.E-06 | 7.E-04 | 1.66 | 3.E-05 | 3.E-03 |
| 17804598 | LOC100360057 /// LOC100363800 /// Rpl22 | 1.72 | 4.E-06 | 9.E-04 | 1.45 | 3.E-05 | 3.E-03 |
| 17795789 | LOC100360076 | -1.21 | 3.E-03 | 5.E-02 | -1.19 | 4.E-03 | 5.E-02 |
| 17676845 | LOC100360077 /// Znhit1 | 1.21 | 2.E-03 | 4.E-02 | 1.26 | 1.E-03 | 2.E-02 |
| 17700011 | LOC100360157 /// LOC100913039 | -1.28 | 1.E-05 | 2.E-03 | -1.26 | 3.E-04 | 1.E-02 |
| 17858638 | LOC100360252 /// LOC100364129 /// LOC690384 /// Rpl31 | 1.32 | 8.E-06 | 1.E-03 | 1.26 | 7.E-05 | 4.E-03 |
| 17770641 | LOC100360302 | 1.22 | 5.E-04 | 2.E-02 | 1.13 | 2.E-04 | 7.E-03 |
| 17729695 | LOC100360573 | 1.18 | 9.E-03 | 8.E-02 | 1.54 | 7.E-05 | 4.E-03 |
| 17675690 | LOC100360604 /// LOC100361103 /// LOC100361811 /// LOC100362550 /// LOC100362779 /// LOC100363471 /// LOC100363732 /// LOC100364171 /// LOC100364176 /// LOC100365729 /// LOC100365974 /// LOC681195 /// LOC682478 /// LOC683497 /// RGD1561815 /// RGD1562601 /// RGD1562839 /// RGD1562923 /// RGD1562929 /// RGD1562971 /// RGD1565370 /// Rpl21 | -1.16 | 4.E-03 | 5.E-02 | -1.12 | 9.E-03 | 8.E-02 |
| 17766258 | LOC100360642 | 1.29 | 9.E-03 | 8.E-02 | 1.5 | 5.E-04 | 1.E-02 |
| 17648697 | LOC100360654 /// LOC100360781 /// LOC100360841 /// LOC100364396 /// LOC100365214 /// LOC690840 /// RGD1561310 /// Rpl37 | 1.16 | 2.E-03 | 4.E-02 | 1.34 | 1.E-06 | 6.E-04 |
| 17663323 | LOC100360679 /// LOC100362298 /// Rps18 | 1.34 | 4.E-05 | 3.E-03 | 1.19 | 1.E-03 | 2.E-02 |
| 17753238 | LOC100360679 /// LOC100362298 /// Rps18 | 1.26 | 1.E-04 | 6.E-03 | 1.18 | 1.E-04 | 5.E-03 |
| 17684237 | LOC100360682 | 1.18 | 8.E-04 | 2.E-02 | 1.13 | 9.E-03 | 8.E-02 |
| 17769798 | LOC100360704 | 1.17 | 3.E-03 | 5.E-02 | 1.26 | 6.E-06 | 1.E-03 |
| 17758316 | LOC100360936 | -1.23 | 2.E-04 | 9.E-03 | -1.19 | 8.E-03 | 7.E-02 |
| 17627043 | LOC100360980 | -1.23 | 7.E-03 | 7.E-02 | -1.35 | 1.E-04 | 5.E-03 |
| 17687869 | LOC100361138 /// LOC100363819 | 1.3 | 2.E-04 | 8.E-03 | 1.21 | 3.E-04 | 9.E-03 |
| 17749165 | LOC100361349 /// LOC100361455 | -1.21 | 2.E-03 | 4.E-02 | -1.16 | 3.E-03 | 4.E-02 |
| 17880297 | LOC100361891 /// Rab5a | -1.21 | 3.E-03 | 4.E-02 | -1.25 | 3.E-06 | 9.E-04 |
| 17644435 | LOC100362124 /// LOC100363962 | 1.17 | 5.E-03 | 6.E-02 | 1.15 | 2.E-03 | 3.E-02 |
| 17681821 | LOC100362384 | -1.18 | 5.E-03 | 6.E-02 | 1.15 | 2.E-03 | 3.E-02 |
| 17804980 | LOC100362419 /// LOC100364191 | 1.33 | 6.E-03 | 6.E-02 | 1.56 | 5.E-06 | 1.E-03 |
| 17840836 | LOC100362634 /// Mll2 | 1.49 | 1.E-04 | 6.E-03 | 1.28 | 3.E-07 | 3.E-04 |
| 17758890 | LOC100362741 /// LOC100362791 /// RGD1565576 | -1.24 | 3.E-03 | 4.E-02 | -1.18 | 1.E-02 | 8.E-02 |
| 17623850 | LOC100362920 | 1.21 | 7.E-05 | 5.E-03 | 1.35 | 4.E-07 | 4.E-04 |
| 17775186 | LOC100362957 /// LOC100364247 | -1.18 | 7.E-03 | 7.E-02 | -1.25 | 2.E-04 | 8.E-03 |
| 17880994 | LOC100362987 | 1.4 | 3.E-05 | 3.E-03 | 1.34 | 8.E-05 | 4.E-03 |
| 17749347 | LOC100363361 | 1.3 | 2.E-04 | 8.E-03 | 1.25 | 6.E-05 | 4.E-03 |
| 17639434 | LOC100363782 /// Rab1b | -1.3 | 5.E-05 | 4.E-03 | -1.19 | 8.E-05 | 4.E-03 |
| 17706778 | LOC100364284 /// Myo9b | 1.38 | 4.E-06 | 9.E-04 | 1.37 | 2.E-06 | 8.E-04 |
| 17653796 | LOC100909417 | -1.3 | 3.E-03 | 4.E-02 | -1.34 | 1.E-04 | 6.E-03 |
| 17643426 | LOC100910712 /// Ntan1 | -1.2 | 4.E-03 | 5.E-02 | -1.31 | 3.E-04 | 9.E-03 |
| 17649424 | LOC100911973 | -1.11 | 8.E-03 | 8.E-02 | -1.23 | 8.E-05 | 4.E-03 |
| 17690285 | LOC100912366 | 1.28 | 1.E-03 | 2.E-02 | 1.25 | 2.E-03 | 3.E-02 |
| 17680277 | LOC289035 | -1.16 | 9.E-03 | 8.E-02 | -1.26 | 2.E-03 | 3.E-02 |
| 17867856 | LOC301126 | 1.17 | 5.E-03 | 6.E-02 | 1.17 | 2.E-03 | 3.E-02 |
| 17869255 | LOC301128 | 1.23 | 8.E-06 | 1.E-03 | 1.16 | 5.E-04 | 1.E-02 |
| 17867345 | LOC310926 /// Rn28s /// Rtn4 | 1.22 | 2.E-07 | 2.E-04 | 1.15 | 4.E-04 | 1.E-02 |
| 17792762 | LOC312502 | 1.13 | 2.E-05 | 3.E-03 | 1.17 | 4.E-03 | 5.E-02 |
| 17826927 | LOC314655 | 1.3 | 3.E-04 | 1.E-02 | 1.43 | 6.E-06 | 1.E-03 |
| 17828910 | LOC363846 /// Vps13b | 1.21 | 2.E-04 | 1.E-02 | 1.1 | 6.E-03 | 6.E-02 |
| 17813247 | LOC474147 | 1.17 | 5.E-03 | 6.E-02 | 1.18 | 9.E-03 | 8.E-02 |
| 17619131 | LOC499219 | -1.19 | 4.E-03 | 5.E-02 | -1.19 | 6.E-03 | 6.E-02 |
| 17806140 | LOC500420 | -1.26 | 9.E-03 | 8.E-02 | -1.19 | 5.E-03 | 5.E-02 |
| 17809694 | LOC500532 | 1.14 | 6.E-04 | 2.E-02 | 1.1 | 5.E-03 | 5.E-02 |
| 17819164 | LOC500726 | 1.24 | 3.E-03 | 5.E-02 | 1.17 | 5.E-04 | 1.E-02 |
| 17881016 | LOC501335 /// LOC501422 /// LOC685331 /// LOC685449 /// LOC689141 /// RGD1564364 | -1.22 | 8.E-03 | 8.E-02 | -1.14 | 6.E-03 | 6.E-02 |
| 17881012 | LOC501441 | -1.13 | 8.E-04 | 2.E-02 | -1.28 | 4.E-05 | 3.E-03 |
| 17613636 | LOC678741 | 1.18 | 1.E-03 | 3.E-02 | 1.24 | 7.E-06 | 1.E-03 |
| 17685833 | LOC684623 | 1.39 | 2.E-04 | 8.E-03 | 1.17 | 3.E-03 | 4.E-02 |
| 17627428 | LOC685045 | 1.32 | 3.E-04 | 1.E-02 | 1.2 | 9.E-07 | 5.E-04 |
| 17808428 | LOC685069 /// LOC685909 /// LOC689408 /// RGD1560813 | 1.52 | 1.E-03 | 3.E-02 | 1.86 | 6.E-04 | 1.E-02 |
| 17825710 | LOC685179 | 1.19 | 8.E-03 | 8.E-02 | 1.16 | 5.E-05 | 3.E-03 |
| 17763704 | LOC686641 /// Olr606 | -1.23 | 3.E-03 | 5.E-02 | -1.25 | 3.E-03 | 4.E-02 |
| 17742664 | LOC687064 | -1.15 | 3.E-03 | 4.E-02 | -1.21 | 6.E-03 | 6.E-02 |
| 17677689 | LOC687565 | 1.26 | 2.E-03 | 3.E-02 | 1.14 | 1.E-03 | 2.E-02 |
| 17881369 | LOC688318 /// Cyp27b1 /// Chchd2 /// LOC684871 /// Fabp5 /// Lce1l /// Lsm2 /// LOC100362916 /// LOC682812 /// LOC684372 /// LOC100366231 /// LOC682352 ///LOC100910788 /// LOC501317 /// LOC684892 /// LOC100909462 /// LOC689635 /// ENSRNOT00000042150 // LOC100909409 /// LOC100366231 /// // LOC100911002 /// LOC684871 /// LOC100910163 /// LOC684892 /// LOC100911516 // rno-mir-3584 // rno-mir-3584 | 1.18 | 3.E-03 | 4.E-02 | 1.12 | 2.E-03 | 3.E-02 |
| 17881419 | LOC688318 /// Cyp27b1 /// Chchd2 /// LOC684871 /// Fabp5 /// Lce1l /// Lsm2 /// LOC100362916 /// LOC682812 /// LOC684372 /// LOC100366231 /// LOC682352 ///LOC100910788 /// LOC501317 /// LOC684892 /// LOC100909462 /// LOC689635 /// ENSRNOT00000042150 // LOC100909409 /// LOC100366231 /// // LOC100911002 /// LOC684871 /// LOC100910163 /// LOC684892 /// LOC100911516 // rno-mir-3584 // rno-mir-3584 | -1.31 | 8.E-05 | 5.E-03 | -1.21 | 3.E-05 | 3.E-03 |
| 17881475 | LOC688318 /// Cyp27b1 /// Chchd2 /// LOC684871 /// Fabp5 /// Lce1l /// Lsm2 /// LOC100362916 /// LOC682812 /// LOC684372 /// LOC100366231 /// LOC682352 ///LOC100910788 /// LOC501317 /// LOC684892 /// LOC100909462 /// LOC689635 /// ENSRNOT00000042150 // LOC100909409 /// LOC100366231 /// // LOC100911002 /// LOC684871 /// LOC100910163 /// LOC684892 /// LOC100911516 // rno-mir-3584 // rno-mir-3584 | 1.27 | 8.E-04 | 2.E-02 | 1.3 | 2.E-04 | 6.E-03 |
| 17881589 | LOC688318 /// Cyp27b1 /// Chchd2 /// LOC684871 /// Fabp5 /// Lce1l /// Lsm2 /// LOC100362916 /// LOC682812 /// LOC684372 /// LOC100366231 /// LOC682352 ///LOC100910788 /// LOC501317 /// LOC684892 /// LOC100909462 /// LOC689635 /// ENSRNOT00000042150 // LOC100909409 /// LOC100366231 /// // LOC100911002 /// LOC684871 /// LOC100910163 /// LOC684892 /// LOC100911516 // rno-mir-3584 // rno-mir-3584 | 1.22 | 3.E-04 | 1.E-02 | 1.13 | 3.E-03 | 4.E-02 |
| 17881635 | LOC688318 /// Cyp27b1 /// Chchd2 /// LOC684871 /// Fabp5 /// Lce1l /// Lsm2 /// LOC100362916 /// LOC682812 /// LOC684372 /// LOC100366231 /// LOC682352 ///LOC100910788 /// LOC501317 /// LOC684892 /// LOC100909462 /// LOC689635 /// ENSRNOT00000042150 // LOC100909409 /// LOC100366231 /// // LOC100911002 /// LOC684871 /// LOC100910163 /// LOC684892 /// LOC100911516 // rno-mir-3584 // rno-mir-3584 | 1.21 | 2.E-03 | 4.E-02 | 1.21 | 8.E-04 | 2.E-02 |
| 17881671 | LOC688318 /// Cyp27b1 /// Chchd2 /// LOC684871 /// Fabp5 /// Lce1l /// Lsm2 /// LOC100362916 /// LOC682812 /// LOC684372 /// LOC100366231 /// LOC682352 ///LOC100910788 /// LOC501317 /// LOC684892 /// LOC100909462 /// LOC689635 /// ENSRNOT00000042150 // LOC100909409 /// LOC100366231 /// // LOC100911002 /// LOC684871 /// LOC100910163 /// LOC684892 /// LOC100911516 // rno-mir-3584 // rno-mir-3584 | 1.29 | 4.E-04 | 1.E-02 | 1.21 | 2.E-06 | 8.E-04 |
| 17881767 | LOC688318 /// Cyp27b1 /// Chchd2 /// LOC684871 /// Fabp5 /// Lce1l /// Lsm2 /// LOC100362916 /// LOC682812 /// LOC684372 /// LOC100366231 /// LOC682352 ///LOC100910788 /// LOC501317 /// LOC684892 /// LOC100909462 /// LOC689635 /// ENSRNOT00000042150 // LOC100909409 /// LOC100366231 /// // LOC100911002 /// LOC684871 /// LOC100910163 /// LOC684892 /// LOC100911516 // rno-mir-3584 // rno-mir-3584 | 1.19 | 1.E-03 | 3.E-02 | 1.19 | 5.E-04 | 1.E-02 |
| 17881823 | LOC688318 /// Cyp27b1 /// Chchd2 /// LOC684871 /// Fabp5 /// Lce1l /// Lsm2 /// LOC100362916 /// LOC682812 /// LOC684372 /// LOC100366231 /// LOC682352 ///LOC100910788 /// LOC501317 /// LOC684892 /// LOC100909462 /// LOC689635 /// ENSRNOT00000042150 // LOC100909409 /// LOC100366231 /// // LOC100911002 /// LOC684871 /// LOC100910163 /// LOC684892 /// LOC100911516 // rno-mir-3584 // rno-mir-3584 | 1.51 | 2.E-05 | 2.E-03 | 1.25 | 2.E-03 | 3.E-02 |
| 17881825 | LOC688318 /// Cyp27b1 /// Chchd2 /// LOC684871 /// Fabp5 /// Lce1l /// Lsm2 /// LOC100362916 /// LOC682812 /// LOC684372 /// LOC100366231 /// LOC682352 ///LOC100910788 /// LOC501317 /// LOC684892 /// LOC100909462 /// LOC689635 /// ENSRNOT00000042150 // LOC100909409 /// LOC100366231 /// // LOC100911002 /// LOC684871 /// LOC100910163 /// LOC684892 /// LOC100911516 // rno-mir-3584 // rno-mir-3584 | -1.17 | 1.E-05 | 2.E-03 | -1.77 | 1.E-03 | 2.E-02 |
| 17881957 | LOC688318 /// Cyp27b1 /// Chchd2 /// LOC684871 /// Fabp5 /// Lce1l /// Lsm2 /// LOC100362916 /// LOC682812 /// LOC684372 /// LOC100366231 /// LOC682352 ///LOC100910788 /// LOC501317 /// LOC684892 /// LOC100909462 /// LOC689635 /// ENSRNOT00000042150 // LOC100909409 /// LOC100366231 /// // LOC100911002 /// LOC684871 /// LOC100910163 /// LOC684892 /// LOC100911516 // rno-mir-3584 // rno-mir-3584 | 1.21 | 3.E-04 | 1.E-02 | 1.26 | 1.E-05 | 2.E-03 |
| 17881963 | LOC688318 /// Cyp27b1 /// Chchd2 /// LOC684871 /// Fabp5 /// Lce1l /// Lsm2 /// LOC100362916 /// LOC682812 /// LOC684372 /// LOC100366231 /// LOC682352 ///LOC100910788 /// LOC501317 /// LOC684892 /// LOC100909462 /// LOC689635 /// ENSRNOT00000042150 // LOC100909409 /// LOC100366231 /// // LOC100911002 /// LOC684871 /// LOC100910163 /// LOC684892 /// LOC100911516 // rno-mir-3584 // rno-mir-3584 | -1.18 | 3.E-03 | 5.E-02 | -1.25 | 4.E-05 | 3.E-03 |
| 17882133 | LOC688318 /// Cyp27b1 /// Chchd2 /// LOC684871 /// Fabp5 /// Lce1l /// Lsm2 /// LOC100362916 /// LOC682812 /// LOC684372 /// LOC100366231 /// LOC682352 ///LOC100910788 /// LOC501317 /// LOC684892 /// LOC100909462 /// LOC689635 /// ENSRNOT00000042150 // LOC100909409 /// LOC100366231 /// // LOC100911002 /// LOC684871 /// LOC100910163 /// LOC684892 /// LOC100911516 // rno-mir-3584 // rno-mir-3584 | -1.2 | 4.E-06 | 1.E-03 | -1.27 | 3.E-04 | 1.E-02 |
| 17882275 | LOC688318 /// Cyp27b1 /// Chchd2 /// LOC684871 /// Fabp5 /// Lce1l /// Lsm2 /// LOC100362916 /// LOC682812 /// LOC684372 /// LOC100366231 /// LOC682352 ///LOC100910788 /// LOC501317 /// LOC684892 /// LOC100909462 /// LOC689635 /// ENSRNOT00000042150 // LOC100909409 /// LOC100366231 /// // LOC100911002 /// LOC684871 /// LOC100910163 /// LOC684892 /// LOC100911516 // rno-mir-3584 // rno-mir-3584 | 1.25 | 3.E-04 | 1.E-02 | 1.2 | 2.E-05 | 2.E-03 |
| 17882309 | LOC688318 /// Cyp27b1 /// Chchd2 /// LOC684871 /// Fabp5 /// Lce1l /// Lsm2 /// LOC100362916 /// LOC682812 /// LOC684372 /// LOC100366231 /// LOC682352 ///LOC100910788 /// LOC501317 /// LOC684892 /// LOC100909462 /// LOC689635 /// ENSRNOT00000042150 // LOC100909409 /// LOC100366231 /// // LOC100911002 /// LOC684871 /// LOC100910163 /// LOC684892 /// LOC100911516 // rno-mir-3584 // rno-mir-3584 | 1.41 | 7.E-05 | 5.E-03 | 1.26 | 3.E-04 | 9.E-03 |
| 17882395 | LOC688318 /// Cyp27b1 /// Chchd2 /// LOC684871 /// Fabp5 /// Lce1l /// Lsm2 /// LOC100362916 /// LOC682812 /// LOC684372 /// LOC100366231 /// LOC682352 ///LOC100910788 /// LOC501317 /// LOC684892 /// LOC100909462 /// LOC689635 /// ENSRNOT00000042150 // LOC100909409 /// LOC100366231 /// // LOC100911002 /// LOC684871 /// LOC100910163 /// LOC684892 /// LOC100911516 // rno-mir-3584 // rno-mir-3584 | 1.18 | 5.E-03 | 6.E-02 | 1.18 | 4.E-04 | 1.E-02 |
| 17882533 | LOC688318 /// Cyp27b1 /// Chchd2 /// LOC684871 /// Fabp5 /// Lce1l /// Lsm2 /// LOC100362916 /// LOC682812 /// LOC684372 /// LOC100366231 /// LOC682352 ///LOC100910788 /// LOC501317 /// LOC684892 /// LOC100909462 /// LOC689635 /// ENSRNOT00000042150 // LOC100909409 /// LOC100366231 /// // LOC100911002 /// LOC684871 /// LOC100910163 /// LOC684892 /// LOC100911516 // rno-mir-3584 // rno-mir-3584 | -1.25 | 1.E-03 | 3.E-02 | -1.1 | 8.E-03 | 7.E-02 |
| 17882539 | LOC688318 /// Cyp27b1 /// Chchd2 /// LOC684871 /// Fabp5 /// Lce1l /// Lsm2 /// LOC100362916 /// LOC682812 /// LOC684372 /// LOC100366231 /// LOC682352 ///LOC100910788 /// LOC501317 /// LOC684892 /// LOC100909462 /// LOC689635 /// ENSRNOT00000042150 // LOC100909409 /// LOC100366231 /// // LOC100911002 /// LOC684871 /// LOC100910163 /// LOC684892 /// LOC100911516 // rno-mir-3584 // rno-mir-3584 | 1.53 | 8.E-07 | 4.E-04 | 1.25 | 3.E-05 | 2.E-03 |
| 17882579 | LOC688318 /// Cyp27b1 /// Chchd2 /// LOC684871 /// Fabp5 /// Lce1l /// Lsm2 /// LOC100362916 /// LOC682812 /// LOC684372 /// LOC100366231 /// LOC682352 ///LOC100910788 /// LOC501317 /// LOC684892 /// LOC100909462 /// LOC689635 /// ENSRNOT00000042150 // LOC100909409 /// LOC100366231 /// // LOC100911002 /// LOC684871 /// LOC100910163 /// LOC684892 /// LOC100911516 // rno-mir-3584 // rno-mir-3584 | 1.22 | 6.E-03 | 6.E-02 | 1.26 | 1.E-05 | 2.E-03 |
| 17882623 | LOC688318 /// Cyp27b1 /// Chchd2 /// LOC684871 /// Fabp5 /// Lce1l /// Lsm2 /// LOC100362916 /// LOC682812 /// LOC684372 /// LOC100366231 /// LOC682352 ///LOC100910788 /// LOC501317 /// LOC684892 /// LOC100909462 /// LOC689635 /// ENSRNOT00000042150 // LOC100909409 /// LOC100366231 /// // LOC100911002 /// LOC684871 /// LOC100910163 /// LOC684892 /// LOC100911516 // rno-mir-3584 // rno-mir-3584 | 1.18 | 8.E-04 | 2.E-02 | 1.17 | 4.E-04 | 1.E-02 |
| 17882655 | LOC688318 /// Cyp27b1 /// Chchd2 /// LOC684871 /// Fabp5 /// Lce1l /// Lsm2 /// LOC100362916 /// LOC682812 /// LOC684372 /// LOC100366231 /// LOC682352 ///LOC100910788 /// LOC501317 /// LOC684892 /// LOC100909462 /// LOC689635 /// ENSRNOT00000042150 // LOC100909409 /// LOC100366231 /// // LOC100911002 /// LOC684871 /// LOC100910163 /// LOC684892 /// LOC100911516 // rno-mir-3584 // rno-mir-3584 | -1.35 | 5.E-04 | 2.E-02 | -1.22 | 6.E-03 | 6.E-02 |
| 17883179 | LOC688318 /// Cyp27b1 /// Chchd2 /// LOC684871 /// Fabp5 /// Lce1l /// Lsm2 /// LOC100362916 /// LOC682812 /// LOC684372 /// LOC100366231 /// LOC682352 ///LOC100910788 /// LOC501317 /// LOC684892 /// LOC100909462 /// LOC689635 /// ENSRNOT00000042150 // LOC100909409 /// LOC100366231 /// // LOC100911002 /// LOC684871 /// LOC100910163 /// LOC684892 /// LOC100911516 // rno-mir-3584 // rno-mir-3584 | 1.75 | 2.E-04 | 9.E-03 | 2.45 | 6.E-05 | 4.E-03 |
| 17883185 | LOC688318 /// Cyp27b1 /// Chchd2 /// LOC684871 /// Fabp5 /// Lce1l /// Lsm2 /// LOC100362916 /// LOC682812 /// LOC684372 /// LOC100366231 /// LOC682352 ///LOC100910788 /// LOC501317 /// LOC684892 /// LOC100909462 /// LOC689635 /// ENSRNOT00000042150 // LOC100909409 /// LOC100366231 /// // LOC100911002 /// LOC684871 /// LOC100910163 /// LOC684892 /// LOC100911516 // rno-mir-3584 // rno-mir-3584 | 1.39 | 1.E-03 | 3.E-02 | 2.31 | 3.E-06 | 9.E-04 |
| 17883191 | LOC688318 /// Cyp27b1 /// Chchd2 /// LOC684871 /// Fabp5 /// Lce1l /// Lsm2 /// LOC100362916 /// LOC682812 /// LOC684372 /// LOC100366231 /// LOC682352 ///LOC100910788 /// LOC501317 /// LOC684892 /// LOC100909462 /// LOC689635 /// ENSRNOT00000042150 // LOC100909409 /// LOC100366231 /// // LOC100911002 /// LOC684871 /// LOC100910163 /// LOC684892 /// LOC100911516 // rno-mir-3584 // rno-mir-3584 | 1.55 | 1.E-04 | 8.E-03 | 1.67 | 9.E-04 | 2.E-02 |
| 17883195 | LOC688318 /// Cyp27b1 /// Chchd2 /// LOC684871 /// Fabp5 /// Lce1l /// Lsm2 /// LOC100362916 /// LOC682812 /// LOC684372 /// LOC100366231 /// LOC682352 ///LOC100910788 /// LOC501317 /// LOC684892 /// LOC100909462 /// LOC689635 /// ENSRNOT00000042150 // LOC100909409 /// LOC100366231 /// // LOC100911002 /// LOC684871 /// LOC100910163 /// LOC684892 /// LOC100911516 // rno-mir-3584 // rno-mir-3584 | 1.37 | 3.E-05 | 3.E-03 | 1.5 | 4.E-06 | 1.E-03 |
| 17883217 | LOC688318 /// Cyp27b1 /// Chchd2 /// LOC684871 /// Fabp5 /// Lce1l /// Lsm2 /// LOC100362916 /// LOC682812 /// LOC684372 /// LOC100366231 /// LOC682352 ///LOC100910788 /// LOC501317 /// LOC684892 /// LOC100909462 /// LOC689635 /// ENSRNOT00000042150 // LOC100909409 /// LOC100366231 /// // LOC100911002 /// LOC684871 /// LOC100910163 /// LOC684892 /// LOC100911516 // rno-mir-3584 // rno-mir-3584 | 1.67 | 5.E-04 | 2.E-02 | 1.92 | 2.E-04 | 8.E-03 |
| 17883219 | LOC688318 /// Cyp27b1 /// Chchd2 /// LOC684871 /// Fabp5 /// Lce1l /// Lsm2 /// LOC100362916 /// LOC682812 /// LOC684372 /// LOC100366231 /// LOC682352 ///LOC100910788 /// LOC501317 /// LOC684892 /// LOC100909462 /// LOC689635 /// ENSRNOT00000042150 // LOC100909409 /// LOC100366231 /// // LOC100911002 /// LOC684871 /// LOC100910163 /// LOC684892 /// LOC100911516 // rno-mir-3584 // rno-mir-3584 | 1.15 | 4.E-07 | 3.E-04 | 1.11 | 5.E-05 | 3.E-03 |
| 17880517 | LOC688411 | -1.19 | 5.E-03 | 6.E-02 | -1.12 | 4.E-03 | 4.E-02 |
| 17880242 | LOC688812 | -1.23 | 4.E-04 | 1.E-02 | -1.14 | 5.E-03 | 5.E-02 |
| 17790395 | LOC688916 | 1.18 | 7.E-03 | 7.E-02 | 1.18 | 7.E-03 | 6.E-02 |
| 17615656 | LOC690136 /// Zfp939 | 1.3 | 3.E-03 | 4.E-02 | 1.31 | 7.E-03 | 7.E-02 |
| 17796460 | LOC690728 | 1.23 | 2.E-05 | 2.E-03 | 1.14 | 1.E-04 | 5.E-03 |
| 17653410 | LOC691137 | -1.36 | 1.E-04 | 6.E-03 | -1.41 | 1.E-04 | 6.E-03 |
| 17880931 | LOC691877 | -1.23 | 8.E-04 | 2.E-02 | -1.18 | 2.E-04 | 7.E-03 |
| 17880338 | LOC692017 | -1.23 | 8.E-04 | 2.E-02 | -1.28 | 4.E-05 | 3.E-03 |
| 17867832 | Lonp1 | -1.2 | 2.E-04 | 8.E-03 | -1.13 | 4.E-03 | 4.E-02 |
| 17768557 | Lpin3 | 1.2 | 6.E-06 | 1.E-03 | 1.35 | 2.E-04 | 6.E-03 |
| 17842618 | Lppr2 | 1.16 | 2.E-03 | 4.E-02 | 1.12 | 8.E-03 | 7.E-02 |
| 17672609 | Lrch4 | 1.2 | 4.E-04 | 1.E-02 | 1.12 | 3.E-03 | 4.E-02 |
| 17630272 | Lrrc68 | 1.27 | 8.E-05 | 5.E-03 | 1.18 | 5.E-03 | 5.E-02 |
| 17660076 | Luc7l3 | 1.28 | 2.E-03 | 3.E-02 | 1.27 | 3.E-07 | 3.E-04 |
| 17803163 | Luzp1 | 1.48 | 7.E-03 | 7.E-02 | 1.22 | 8.E-03 | 7.E-02 |
| 17838137 | Lypd2 | -1.19 | 5.E-03 | 6.E-02 | -3.84 | 9.E-05 | 5.E-03 |
| 17687448 | Lyplal1 | -1.37 | 1.E-06 | 5.E-04 | -1.24 | 4.E-03 | 5.E-02 |
| 17774458 | Madd | 1.36 | 1.E-03 | 3.E-02 | 1.22 | 2.E-04 | 8.E-03 |
| 17793404 | Magi1 | 1.24 | 3.E-03 | 4.E-02 | 1.27 | 1.E-05 | 2.E-03 |
| 17759882 | Man1b1 | 1.24 | 1.E-05 | 2.E-03 | 1.16 | 5.E-04 | 1.E-02 |
| 17806222 | Manea | -1.31 | 3.E-03 | 4.E-02 | -1.33 | 4.E-04 | 1.E-02 |
| 17675053 | Map2k7 | 1.18 | 1.E-04 | 7.E-03 | 1.17 | 2.E-04 | 7.E-03 |
| 17623421 | Map4k2 | 1.13 | 3.E-04 | 1.E-02 | 1.26 | 2.E-06 | 8.E-04 |
| 17858656 | Map4k4 | 1.49 | 2.E-03 | 3.E-02 | 1.26 | 2.E-03 | 3.E-02 |
| 17654606 | Mapk8ip3 | 1.34 | 5.E-05 | 4.E-03 | 1.26 | 3.E-05 | 3.E-03 |
| 17765197 | Mapkbp1 | 1.11 | 5.E-03 | 6.E-02 | 1.19 | 5.E-04 | 1.E-02 |
| 17640135 | Mark2 | 1.39 | 8.E-05 | 5.E-03 | 1.13 | 9.E-03 | 8.E-02 |
| 17722387 | Matr3 | 1.2 | 2.E-04 | 1.E-02 | 1.19 | 7.E-05 | 4.E-03 |
| 17707260 | Mau2 | 1.17 | 2.E-05 | 2.E-03 | 1.18 | 3.E-06 | 1.E-03 |
| 17836505 | Mbd6 | 1.25 | 1.E-03 | 3.E-02 | 1.27 | 1.E-05 | 2.E-03 |
| 17706667 | Mbl1 | -1.21 | 2.E-03 | 4.E-02 | -1.64 | 5.E-04 | 1.E-02 |
| 17872419 | Mbtps2 | -1.3 | 4.E-06 | 1.E-03 | -1.19 | 4.E-03 | 5.E-02 |
| 17757721 | Mcm3ap | 1.19 | 6.E-04 | 2.E-02 | 1.12 | 1.E-03 | 2.E-02 |
| 17875407 | Mcts1 /// Mcts2 | 1.29 | 3.E-04 | 1.E-02 | 1.17 | 5.E-03 | 6.E-02 |
| 17880629 | Mcts2 | 1.35 | 3.E-03 | 4.E-02 | 1.23 | 6.E-04 | 1.E-02 |
| 17857449 | Mea1 | -1.2 | 7.E-05 | 5.E-03 | -1.22 | 1.E-04 | 6.E-03 |
| 17678341 | Med13l | 1.25 | 2.E-03 | 3.E-02 | 1.12 | 5.E-03 | 5.E-02 |
| 17694890 | Med28 | -1.34 | 6.E-06 | 1.E-03 | -1.17 | 9.E-03 | 8.E-02 |
| 17614403 | Megf8 | 1.3 | 7.E-06 | 1.E-03 | 1.23 | 6.E-04 | 1.E-02 |
| 17623408 | Men1 | 1.11 | 4.E-03 | 5.E-02 | 1.15 | 1.E-03 | 2.E-02 |
| 17773118 | Mettl5 | 1.31 | 1.E-03 | 2.E-02 | 1.4 | 2.E-03 | 3.E-02 |
| 17794469 | Mical3 | 1.36 | 5.E-04 | 2.E-02 | 1.28 | 1.E-05 | 2.E-03 |
| 17647841 | Mink1 | 1.37 | 5.E-04 | 2.E-02 | 1.26 | 6.E-05 | 4.E-03 |
| 17790272 | Mir183 | -1.23 | 1.E-03 | 3.E-02 | -1.18 | 4.E-04 | 1.E-02 |
| 17743669 | Mir186 | 1.87 | 2.E-03 | 3.E-02 | 1.59 | 3.E-03 | 3.E-02 |
| 17649888 | Mir301a | -1.2 | 4.E-03 | 5.E-02 | -1.27 | 8.E-06 | 2.E-03 |
| 17822008 | Mir3593 /// Ralgapa1 | 1.16 | 1.E-03 | 2.E-02 | 1.14 | 1.E-04 | 6.E-03 |
| 17845815 | Mir628 | 1.18 | 6.E-03 | 7.E-02 | 1.38 | 2.E-06 | 8.E-04 |
| 17786300 | Mkrn2 | -1.17 | 1.E-03 | 3.E-02 | -1.17 | 2.E-05 | 2.E-03 |
| 17631597 | Mll4 | 1.26 | 1.E-05 | 2.E-03 | 1.28 | 5.E-06 | 1.E-03 |
| 17717325 | Mllt10 | 1.12 | 9.E-03 | 8.E-02 | 1.14 | 1.E-04 | 5.E-03 |
| 17654422 | Mlst8 | 1.2 | 7.E-03 | 7.E-02 | 1.32 | 6.E-06 | 1.E-03 |
| 17872173 | Mospd2 | -1.19 | 8.E-04 | 2.E-02 | -1.28 | 1.E-04 | 5.E-03 |
| 17750156 | Mov10 | 1.17 | 7.E-03 | 7.E-02 | 1.19 | 8.E-04 | 2.E-02 |
| 17646347 | Mprip | 1.24 | 2.E-05 | 2.E-03 | 1.14 | 7.E-04 | 1.E-02 |
| 17830400 | Mpst | -1.12 | 6.E-04 | 2.E-02 | -1.22 | 2.E-03 | 3.E-02 |
| 17699338 | Mrp63 | -1.18 | 1.E-04 | 6.E-03 | -1.27 | 3.E-05 | 3.E-03 |
| 17623006 | mrpl11 | -1.27 | 3.E-04 | 1.E-02 | -1.15 | 4.E-04 | 1.E-02 |
| 17623011 | mrpl11 | -1.24 | 3.E-03 | 4.E-02 | -1.17 | 1.E-04 | 5.E-03 |
| 17623012 | mrpl11 | -1.28 | 2.E-03 | 3.E-02 | -1.14 | 4.E-03 | 5.E-02 |
| 17792500 | Mrpl19 | -1.23 | 9.E-04 | 2.E-02 | -1.14 | 6.E-03 | 6.E-02 |
| 17622665 | Mrpl21 | -1.22 | 6.E-05 | 4.E-03 | -1.17 | 4.E-03 | 5.E-02 |
| 17622670 | Mrpl21 | -1.22 | 1.E-03 | 3.E-02 | -1.25 | 7.E-04 | 2.E-02 |
| 17622671 | Mrpl21 | -1.29 | 2.E-04 | 1.E-02 | -1.23 | 6.E-03 | 6.E-02 |
| 17622672 | Mrpl21 | -1.23 | 6.E-04 | 2.E-02 | -1.23 | 5.E-03 | 5.E-02 |
| 17842358 | Mrpl4 | -1.3 | 6.E-07 | 4.E-04 | -1.15 | 1.E-03 | 2.E-02 |
| 17642377 | Mrpl43 | -1.15 | 2.E-03 | 4.E-02 | 1.21 | 1.E-04 | 5.E-03 |
| 17642378 | Mrpl43 | -1.17 | 6.E-03 | 7.E-02 | 1.17 | 2.E-03 | 3.E-02 |
| 17634072 | Mrpl46 | -1.61 | 1.E-06 | 6.E-04 | -1.39 | 2.E-04 | 7.E-03 |
| 17784758 | Mrpl53 | -1.12 | 2.E-03 | 3.E-02 | -1.15 | 4.E-04 | 1.E-02 |
| 17784759 | Mrpl53 | -1.16 | 5.E-03 | 6.E-02 | -1.21 | 1.E-05 | 2.E-03 |
| 17697181 | Mrps16 | 1.23 | 2.E-04 | 1.E-02 | 1.24 | 3.E-05 | 2.E-03 |
| 17673348 | Mrps17 | -1.17 | 4.E-03 | 5.E-02 | -1.2 | 9.E-03 | 8.E-02 |
| 17650901 | Msl1 | 1.21 | 1.E-03 | 3.E-02 | 1.21 | 3.E-03 | 4.E-02 |
| 17819244 | Mta1 | 1.2 | 2.E-03 | 4.E-02 | 1.14 | 1.E-03 | 2.E-02 |
| 17740926 | Mtmr11 | 1.27 | 1.E-03 | 3.E-02 | 1.21 | 6.E-06 | 1.E-03 |
| 17842016 | Mtmr2 | -1.11 | 5.E-03 | 6.E-02 | -1.11 | 2.E-03 | 3.E-02 |
| 17626420 | Mxi1 | -1.24 | 8.E-04 | 2.E-02 | -1.18 | 3.E-03 | 4.E-02 |
| 17642889 | Mxi1 | -1.24 | 8.E-04 | 2.E-02 | -1.18 | 3.E-03 | 4.E-02 |
| 17776616 | Myef2 | 1.42 | 4.E-05 | 3.E-03 | 1.17 | 3.E-03 | 4.E-02 |
| 17694422 | N4bp2 | 1.22 | 9.E-04 | 2.E-02 | 1.15 | 6.E-04 | 1.E-02 |
| 17873738 | Nap1l3 | -1.19 | 8.E-03 | 8.E-02 | -1.15 | 7.E-03 | 6.E-02 |
| 17855957 | Nbeal2 | 1.3 | 7.E-06 | 1.E-03 | 1.31 | 4.E-06 | 1.E-03 |
| 17867282 | NC_001665 | 1.41 | 1.E-05 | 2.E-03 | 1.51 | 6.E-06 | 1.E-03 |
| 17867328 | NC_001665 | 1.33 | 4.E-03 | 5.E-02 | 2.18 | 3.E-05 | 3.E-03 |
| 17796805 | Ncoa2 | 1.24 | 4.E-05 | 3.E-03 | 1.13 | 2.E-04 | 6.E-03 |
| 17779331 | Ncoa5 | 1.12 | 1.E-03 | 3.E-02 | 1.16 | 6.E-04 | 1.E-02 |
| 17673537 | Ncor2 | 1.36 | 1.E-04 | 7.E-03 | 1.33 | 4.E-05 | 3.E-03 |
| 17700582 | Ndfip2 | -1.21 | 4.E-04 | 1.E-02 | -1.31 | 9.E-05 | 5.E-03 |
| 17770205 | Ndor1 | 1.15 | 5.E-03 | 6.E-02 | 1.2 | 1.E-05 | 2.E-03 |
| 17697044 | Ndst2 | 1.22 | 2.E-05 | 2.E-03 | 1.18 | 4.E-04 | 1.E-02 |
| 17795067 | Ndufa9 | -1.15 | 5.E-03 | 6.E-02 | -1.17 | 1.E-03 | 2.E-02 |
| 17859523 | Ndufb3 | 1.15 | 5.E-03 | 6.E-02 | 1.32 | 9.E-06 | 2.E-03 |
| 17806434 | Ndufb6 | -1.24 | 4.E-04 | 1.E-02 | -1.3 | 3.E-04 | 1.E-02 |
| 17837542 | Ndufb9 | -1.14 | 4.E-03 | 5.E-02 | -1.21 | 7.E-04 | 2.E-02 |
| 17865002 | Ndufs1 | -1.14 | 9.E-04 | 2.E-02 | -1.2 | 2.E-03 | 3.E-02 |
| 17745318 | Ndufs4 | -1.33 | 4.E-05 | 3.E-03 | -1.29 | 1.E-05 | 2.E-03 |
| 17866987 | Ndufv2 | -1.19 | 6.E-04 | 2.E-02 | -1.11 | 3.E-03 | 4.E-02 |
| 17715072 | Nedd9 | 1.29 | 3.E-04 | 1.E-02 | 1.16 | 8.E-03 | 7.E-02 |
| 17760286 | Nelf | 1.12 | 1.E-02 | 9.E-02 | 1.24 | 1.E-03 | 2.E-02 |
| 17756505 | Neu1 | -1.28 | 3.E-04 | 1.E-02 | -1.35 | 6.E-03 | 6.E-02 |
| 17647558 | Neurl4 | 1.22 | 4.E-04 | 1.E-02 | 1.22 | 1.E-04 | 6.E-03 |
| 17826037 | Nfic | 1.32 | 5.E-04 | 2.E-02 | 1.22 | 5.E-04 | 1.E-02 |
| 17631163 | Nfkbib | -1.21 | 3.E-03 | 4.E-02 | -1.17 | 2.E-03 | 3.E-02 |
| 17741409 | Ngf | 1.19 | 4.E-03 | 5.E-02 | -1.27 | 4.E-03 | 4.E-02 |
| 17701265 | Ngly1 | 1.18 | 1.E-04 | 7.E-03 | 1.15 | 3.E-04 | 1.E-02 |
| 17645647 | Nhp2 | 1.26 | 1.E-03 | 2.E-02 | 1.19 | 4.E-04 | 1.E-02 |
| 17859513 | Nif3l1 | 1.17 | 4.E-03 | 5.E-02 | 1.25 | 1.E-03 | 2.E-02 |
| 17709944 | Nisch | 1.2 | 2.E-04 | 9.E-03 | 1.12 | 3.E-04 | 9.E-03 |
| 17665236 | Nit2 | -1.16 | 8.E-07 | 4.E-04 | -1.14 | 9.E-03 | 8.E-02 |
| 17611354 | Nkd2 | -1.16 | 6.E-04 | 2.E-02 | 1.16 | 7.E-03 | 6.E-02 |
| 17644425 | Nme3 | -1.4 | 6.E-06 | 1.E-03 | -1.25 | 1.E-03 | 2.E-02 |
| 17778926 | Nnat | 1.46 | 5.E-05 | 4.E-03 | 1.45 | 1.E-04 | 5.E-03 |
| 17805181 | Noc2l | 1.2 | 6.E-04 | 2.E-02 | 1.15 | 3.E-03 | 4.E-02 |
| 17759923 | Npdc1 | 1.16 | 9.E-03 | 8.E-02 | 1.26 | 1.E-05 | 2.E-03 |
| 17717829 | Npepo | 1.21 | 2.E-03 | 4.E-02 | 1.33 | 8.E-06 | 1.E-03 |
| 17644805 | Nprl3 | 1.11 | 4.E-03 | 5.E-02 | 1.21 | 7.E-05 | 4.E-03 |
| 17719104 | Nqo2 | -1.22 | 4.E-03 | 5.E-02 | -1.39 | 4.E-03 | 5.E-02 |
| 17718176 | Nsd1 | 1.3 | 2.E-05 | 2.E-03 | 1.15 | 3.E-05 | 2.E-03 |
| 17645043 | Nudcd2 | -1.23 | 4.E-04 | 1.E-02 | -1.28 | 8.E-04 | 2.E-02 |
| 17757096 | Nudt3 | 1.18 | 2.E-04 | 9.E-03 | 1.13 | 5.E-03 | 5.E-02 |
| 17618936 | Numa1 | 1.16 | 2.E-03 | 3.E-02 | 1.19 | 2.E-03 | 3.E-02 |
| 17760946 | Nup188 | 1.21 | 2.E-05 | 2.E-03 | 1.13 | 7.E-04 | 2.E-02 |
| 17767058 | Nxt1 | -1.18 | 4.E-03 | 5.E-02 | -1.22 | 2.E-06 | 8.E-04 |
| 17867332 | Oaz2 | -1.99 | 2.E-04 | 9.E-03 | 2.09 | 5.E-03 | 5.E-02 |
| 17694192 | Ociad1 | -1.26 | 1.E-05 | 2.E-03 | -1.28 | 4.E-04 | 1.E-02 |
| 17718561 | Ogn | -1.35 | 7.E-03 | 7.E-02 | -1.6 | 1.E-03 | 2.E-02 |
| 17873284 | Ogt | 1.3 | 1.E-04 | 6.E-03 | 1.21 | 3.E-05 | 2.E-03 |
| 17864771 | Orc2 | 1.27 | 4.E-04 | 1.E-02 | 1.12 | 7.E-03 | 6.E-02 |
| 17769532 | Osbpl2 | -1.24 | 6.E-05 | 5.E-03 | -1.21 | 9.E-03 | 8.E-02 |
| 17769535 | Osbpl2 | 1.23 | 8.E-03 | 7.E-02 | 1.45 | 4.E-05 | 3.E-03 |
| 17650607 | Osbpl7 | 1.13 | 7.E-03 | 7.E-02 | 1.27 | 3.E-05 | 3.E-03 |
| 17755696 | Ostm1 | -1.45 | 2.E-07 | 2.E-04 | -1.21 | 4.E-03 | 5.E-02 |
| 17674882 | P2rx2 | -1.19 | 7.E-03 | 7.E-02 | -1.21 | 4.E-03 | 5.E-02 |
| 17664001 | P4hb | 1.12 | 8.E-05 | 5.E-03 | 1.14 | 1.E-04 | 6.E-03 |
| 17859802 | Pard3b | 1.43 | 1.E-03 | 3.E-02 | 1.21 | 2.E-05 | 2.E-03 |
| 17812905 | Park7 | -1.19 | 1.E-04 | 7.E-03 | -1.19 | 6.E-03 | 6.E-02 |
| 17754847 | Pbld | -1.23 | 1.E-03 | 3.E-02 | -1.24 | 3.E-05 | 3.E-03 |
| 17754983 | Pcbd1 | -1.1 | 4.E-04 | 1.E-02 | -1.25 | 5.E-03 | 5.E-02 |
| 17642625 | Pcgf6 | -1.17 | 4.E-03 | 5.E-02 | -1.22 | 2.E-03 | 3.E-02 |
| 17639653 | Pcnxl3 | 1.25 | 2.E-06 | 7.E-04 | 1.13 | 1.E-03 | 2.E-02 |
| 17792839 | Pcyox1 | -1.22 | 2.E-03 | 3.E-02 | -1.43 | 2.E-04 | 7.E-03 |
| 17669787 | Pcyt1a | 1.15 | 5.E-03 | 6.E-02 | 1.14 | 5.E-03 | 5.E-02 |
| 17664031 | Pcyt2 | 1.2 | 8.E-03 | 8.E-02 | 1.28 | 5.E-03 | 5.E-02 |
| 17626494 | Pdcd4 | -1.59 | 4.E-08 | 6.E-05 | -1.21 | 4.E-03 | 5.E-02 |
| 17751690 | Pdlim5 | 1.22 | 2.E-06 | 7.E-04 | 1.19 | 1.E-03 | 2.E-02 |
| 17615501 | Pepd | -1.44 | 1.E-04 | 6.E-03 | -1.39 | 2.E-04 | 7.E-03 |
| 17627354 | Pex3 | -1.41 | 1.E-05 | 2.E-03 | -1.33 | 1.E-05 | 2.E-03 |
| 17828810 | Pgcp | -1.24 | 4.E-03 | 5.E-02 | -1.76 | 1.E-05 | 2.E-03 |
| 17873492 | Pgk1 | -1.3 | 2.E-04 | 9.E-03 | -1.31 | 1.E-04 | 5.E-03 |
| 17875511 | Pgrmc1 | -1.14 | 4.E-04 | 1.E-02 | -1.42 | 3.E-04 | 1.E-02 |
| 17648787 | Phf12 | 1.21 | 1.E-03 | 3.E-02 | 1.15 | 2.E-04 | 8.E-03 |
| 17714849 | Phf2 | 1.23 | 7.E-04 | 2.E-02 | 1.11 | 3.E-03 | 4.E-02 |
| 17764156 | Phf21a | 1.2 | 9.E-03 | 8.E-02 | 1.33 | 9.E-05 | 4.E-03 |
| 17851828 | Phldb1 | 1.27 | 4.E-03 | 5.E-02 | 1.3 | 2.E-04 | 7.E-03 |
| 17830586 | Pick1 | 1.14 | 8.E-04 | 2.E-02 | 1.14 | 6.E-03 | 6.E-02 |
| 17707071 | Pik3r2 | 1.19 | 2.E-04 | 8.E-03 | 1.27 | 3.E-05 | 3.E-03 |
| 17842307 | Pin1 | 1.24 | 1.E-03 | 3.E-02 | 1.26 | 1.E-05 | 2.E-03 |
| 17833416 | Pip5k1c | 1.1 | 8.E-03 | 8.E-02 | 1.18 | 2.E-03 | 3.E-02 |
| 17648607 | Pitpna | -1.21 | 1.E-05 | 2.E-03 | -1.23 | 2.E-05 | 2.E-03 |
| 17622836 | Pitpnm1 | 1.21 | 8.E-03 | 8.E-02 | 1.21 | 1.E-03 | 2.E-02 |
| 17673693 | Pitpnm2 | 1.27 | 8.E-05 | 5.E-03 | 1.27 | 4.E-04 | 1.E-02 |
| 17644256 | Pkd1 | 1.36 | 3.E-08 | 6.E-05 | 1.23 | 3.E-06 | 9.E-04 |
| 17728903 | Pkn1 | 1.21 | 9.E-06 | 2.E-03 | 1.15 | 7.E-03 | 7.E-02 |
| 17768522 | Plcg1 | 1.16 | 1.E-03 | 3.E-02 | 1.15 | 6.E-05 | 4.E-03 |
| 17647812 | Pld2 | 1.18 | 5.E-03 | 6.E-02 | 1.26 | 1.E-02 | 8.E-02 |
| 17812094 | Plekhm2 | 1.11 | 2.E-03 | 4.E-02 | 1.14 | 6.E-03 | 6.E-02 |
| 17812522 | Plod1 | 1.12 | 5.E-03 | 6.E-02 | 1.14 | 7.E-03 | 6.E-02 |
| 17675978 | Pms2 | 1.14 | 1.E-03 | 2.E-02 | 1.21 | 8.E-04 | 2.E-02 |
| 17816023 | Pnn | 1.33 | 3.E-05 | 3.E-03 | 1.26 | 5.E-05 | 3.E-03 |
| 17811536 | Pnrc2 | -1.24 | 1.E-05 | 2.E-03 | -1.37 | 2.E-06 | 8.E-04 |
| 17792507 | Pole4 | -1.13 | 8.E-04 | 2.E-02 | -1.23 | 5.E-06 | 1.E-03 |
| 17830578 | Polr2f | -1.21 | 8.E-05 | 5.E-03 | -1.34 | 1.E-05 | 2.E-03 |
| 17672977 | Pom121 | 1.22 | 1.E-03 | 3.E-02 | 1.17 | 6.E-03 | 6.E-02 |
| 17823417 | Pomt2 | 1.22 | 2.E-03 | 4.E-02 | 1.14 | 1.E-03 | 2.E-02 |
| 17789560 | Pon2 | -1.25 | 4.E-03 | 5.E-02 | -1.35 | 3.E-04 | 9.E-03 |
| 17841182 | Pou6f1 | 1.23 | 3.E-04 | 1.E-02 | 1.24 | 9.E-04 | 2.E-02 |
| 17742738 | Ppa2 | -1.14 | 6.E-03 | 6.E-02 | -1.15 | 2.E-03 | 3.E-02 |
| 17735796 | Ppap2a | -1.25 | 3.E-03 | 4.E-02 | -1.32 | 5.E-04 | 1.E-02 |
| 17845302 | Ppib | -1.18 | 4.E-04 | 1.E-02 | -1.29 | 1.E-04 | 5.E-03 |
| 17810291 | Ppie | 1.24 | 2.E-03 | 4.E-02 | 1.25 | 8.E-04 | 2.E-02 |
| 17828512 | Ppm1h | 1.34 | 5.E-04 | 2.E-02 | 1.16 | 1.E-03 | 2.E-02 |
| 17635241 | Ppme1 | -1.22 | 2.E-03 | 3.E-02 | -1.16 | 7.E-03 | 7.E-02 |
| 17821966 | Ppp2r3c | -1.51 | 1.E-05 | 2.E-03 | -1.28 | 3.E-03 | 4.E-02 |
| 17771923 | Ppp6c | -1.2 | 3.E-04 | 1.E-02 | -1.39 | 3.E-07 | 3.E-04 |
| 17801781 | Ppt1 | -1.32 | 2.E-04 | 8.E-03 | -1.63 | 3.E-05 | 3.E-03 |
| 17774212 | Pramel7 | -1.2 | 9.E-03 | 8.E-02 | -1.33 | 1.E-03 | 2.E-02 |
| 17748573 | Prcc | 1.14 | 5.E-04 | 2.E-02 | 1.23 | 6.E-06 | 1.E-03 |
| 17668296 | Prdm15 | 1.29 | 6.E-04 | 2.E-02 | 1.24 | 3.E-04 | 1.E-02 |
| 17643232 | Prdx3 | -1.18 | 1.E-03 | 2.E-02 | -1.22 | 1.E-05 | 2.E-03 |
| 17808996 | Prkaa2 | -1.26 | 7.E-05 | 5.E-03 | -1.23 | 2.E-03 | 3.E-02 |
| 17618548 | Prkrir | 1.25 | 7.E-03 | 7.E-02 | 1.22 | 2.E-03 | 3.E-02 |
| 17712261 | Prosc | -1.27 | 2.E-03 | 3.E-02 | -1.28 | 4.E-04 | 1.E-02 |
| 17712996 | Proz | -1.17 | 2.E-03 | 4.E-02 | -1.48 | 4.E-03 | 4.E-02 |
| 17816140 | Prpf39 | 1.28 | 4.E-05 | 3.E-03 | 1.21 | 1.E-04 | 6.E-03 |
| 17719048 | Prpf4b | 1.35 | 2.E-06 | 7.E-04 | 1.13 | 6.E-04 | 1.E-02 |
| 17769828 | Prpf6 | 1.1 | 3.E-03 | 4.E-02 | 1.16 | 1.E-04 | 6.E-03 |
| 17648522 | Prpf8 | -1.48 | 3.E-05 | 3.E-03 | -1.52 | 2.E-05 | 2.E-03 |
| 17632658 | Prr12 | 1.15 | 9.E-05 | 5.E-03 | 1.12 | 4.E-03 | 5.E-02 |
| 17752769 | Prrc2a | 1.39 | 1.E-03 | 3.E-02 | 1.2 | 9.E-05 | 4.E-03 |
| 17685980 | Prrc2c | 1.24 | 1.E-03 | 2.E-02 | 1.18 | 1.E-03 | 2.E-02 |
| 17812263 | Prrc2c | 1.23 | 1.E-03 | 3.E-02 | 1.11 | 5.E-05 | 3.E-03 |
| 17707399 | Psd3 | 1.18 | 1.E-03 | 3.E-02 | 1.15 | 6.E-03 | 6.E-02 |
| 17631591 | Psenen | -1.13 | 1.E-03 | 3.E-02 | -1.24 | 3.E-04 | 9.E-03 |
| 17652110 | Psmc5 | 1.27 | 1.E-03 | 3.E-02 | 1.17 | 9.E-04 | 2.E-02 |
| 17749357 | Psmd4 | 1.15 | 7.E-04 | 2.E-02 | 1.13 | 3.E-03 | 4.E-02 |
| 17749936 | Ptgfrn | 1.28 | 7.E-03 | 7.E-02 | 1.2 | 7.E-03 | 6.E-02 |
| 17669453 | Ptplb | -1.2 | 7.E-04 | 2.E-02 | -1.42 | 3.E-06 | 8.E-04 |
| 17769213 | Ptpn1 | 1.26 | 1.E-03 | 3.E-02 | 1.25 | 3.E-03 | 4.E-02 |
| 17852250 | Pts | -1.21 | 8.E-05 | 5.E-03 | -1.23 | 8.E-04 | 2.E-02 |
| 17828652 | R3hdm2 | 1.12 | 8.E-03 | 8.E-02 | 1.12 | 1.E-03 | 2.E-02 |
| 17740281 | Rab13 | 1.21 | 2.E-03 | 4.E-02 | 1.28 | 5.E-05 | 3.E-03 |
| 17866419 | Rab17 | -1.31 | 2.E-04 | 9.E-03 | -1.3 | 1.E-04 | 6.E-03 |
| 17797157 | Rab2a | -1.24 | 9.E-05 | 5.E-03 | -1.2 | 2.E-03 | 3.E-02 |
| 17833050 | Rab5b | 1.23 | 8.E-05 | 5.E-03 | 1.14 | 6.E-03 | 6.E-02 |
| 17793032 | Rab7a | -1.16 | 6.E-07 | 4.E-04 | -1.18 | 1.E-04 | 6.E-03 |
| 17752098 | Rabggtb | -1.26 | 6.E-06 | 1.E-03 | -1.23 | 4.E-04 | 1.E-02 |
| 17646391 | Rai1 | 1.26 | 2.E-05 | 2.E-03 | 1.37 | 6.E-08 | 1.E-04 |
| 17758933 | Ranbp2 | 1.11 | 2.E-04 | 9.E-03 | -1.11 | 9.E-04 | 2.E-02 |
| 17836072 | Rap1b | -1.13 | 9.E-03 | 8.E-02 | -1.19 | 4.E-03 | 5.E-02 |
| 17880246 | Rap1b | -1.25 | 2.E-04 | 8.E-03 | -1.26 | 5.E-04 | 1.E-02 |
| 17880261 | Rap1b | -1.25 | 2.E-04 | 8.E-03 | -1.26 | 5.E-04 | 1.E-02 |
| 17788411 | Rbm33 | 1.28 | 2.E-05 | 2.E-03 | 1.21 | 3.E-06 | 9.E-04 |
| 17855637 | Rbm5 | 1.21 | 2.E-03 | 3.E-02 | 1.14 | 2.E-05 | 2.E-03 |
| 17838842 | Rbm9 | 1.17 | 7.E-03 | 7.E-02 | 1.15 | 2.E-03 | 3.E-02 |
| 17663301 | Recql5 | 1.13 | 2.E-03 | 4.E-02 | 1.16 | 2.E-03 | 3.E-02 |
| 17804431 | Rere | 1.27 | 4.E-05 | 4.E-03 | 1.21 | 2.E-05 | 2.E-03 |
| 17826318 | Rexo1 | 1.23 | 3.E-05 | 3.E-03 | 1.19 | 5.E-04 | 1.E-02 |
| 17705992 | Rft1 | -1.32 | 3.E-04 | 1.E-02 | -1.31 | 6.E-04 | 1.E-02 |
| 17732616 | Rfx1 | 1.18 | 3.E-03 | 4.E-02 | 1.14 | 4.E-03 | 4.E-02 |
| 17754146 | RGD1303003 | 1.21 | 2.E-03 | 3.E-02 | 1.18 | 3.E-03 | 4.E-02 |
| 17764714 | RGD1305045 | -1.26 | 3.E-03 | 4.E-02 | -1.59 | 2.E-05 | 2.E-03 |
| 17815921 | RGD1305089 | -1.14 | 3.E-03 | 5.E-02 | -1.17 | 2.E-03 | 3.E-02 |
| 17790708 | RGD1306271 | 1.18 | 6.E-03 | 7.E-02 | 1.25 | 8.E-04 | 2.E-02 |
| 17835987 | RGD1306474 | -1.32 | 9.E-06 | 1.E-03 | -1.19 | 9.E-03 | 8.E-02 |
| 17663957 | RGD1306926 | 1.18 | 5.E-03 | 6.E-02 | 1.26 | 7.E-04 | 2.E-02 |
| 17783495 | RGD1306936 | -1.28 | 1.E-03 | 2.E-02 | -1.21 | 2.E-04 | 7.E-03 |
| 17709313 | RGD1307041 | -1.2 | 2.E-05 | 2.E-03 | -1.16 | 1.E-03 | 2.E-02 |
| 17777521 | RGD1307399 | -1.23 | 3.E-05 | 3.E-03 | -1.24 | 9.E-05 | 5.E-03 |
| 17845637 | RGD1307526 | 1.23 | 5.E-05 | 4.E-03 | 1.15 | 4.E-03 | 5.E-02 |
| 17770370 | RGD1307615 | 1.11 | 7.E-04 | 2.E-02 | 1.17 | 2.E-04 | 8.E-03 |
| 17705117 | RGD1308297 | 1.24 | 1.E-04 | 6.E-03 | 1.22 | 4.E-05 | 3.E-03 |
| 17698672 | RGD1308430 | -1.37 | 2.E-04 | 9.E-03 | -1.44 | 2.E-05 | 2.E-03 |
| 17769584 | RGD1308612 | 1.19 | 3.E-03 | 5.E-02 | 1.29 | 2.E-06 | 8.E-04 |
| 17700278 | RGD1308772 | 1.33 | 1.E-04 | 7.E-03 | 1.27 | 8.E-04 | 2.E-02 |
| 17672152 | RGD1308908 | 1.29 | 2.E-05 | 2.E-03 | 1.14 | 2.E-03 | 3.E-02 |
| 17612277 | RGD1309374 | 1.18 | 1.E-03 | 3.E-02 | 1.24 | 2.E-05 | 2.E-03 |
| 17700997 | RGD1309414 | 1.23 | 7.E-06 | 1.E-03 | 1.21 | 7.E-06 | 1.E-03 |
| 17710569 | RGD1309676 | -1.26 | 6.E-05 | 4.E-03 | -1.45 | 5.E-04 | 1.E-02 |
| 17678067 | RGD1309762 | 1.29 | 1.E-05 | 2.E-03 | 1.16 | 6.E-05 | 4.E-03 |
| 17800567 | RGD1310313 | -1.18 | 2.E-04 | 8.E-03 | -1.17 | 9.E-04 | 2.E-02 |
| 17689469 | RGD1311309 | 1.2 | 2.E-03 | 3.E-02 | 1.11 | 2.E-03 | 3.E-02 |
| 17714183 | RGD1311345 | -1.33 | 5.E-05 | 4.E-03 | -1.36 | 1.E-04 | 6.E-03 |
| 17620972 | RGD1311624 | 1.27 | 2.E-03 | 3.E-02 | 1.13 | 1.E-02 | 8.E-02 |
| 17880487 | RGD1560402 | -1.19 | 3.E-03 | 4.E-02 | -1.22 | 1.E-03 | 2.E-02 |
| 17880385 | RGD1560648 | -1.38 | 1.E-03 | 3.E-02 | -1.32 | 4.E-04 | 1.E-02 |
| 17724291 | RGD1562987 | -1.39 | 7.E-05 | 5.E-03 | -1.24 | 2.E-04 | 8.E-03 |
| 17747631 | RGD1564666 | -1.17 | 4.E-04 | 1.E-02 | -1.15 | 8.E-03 | 7.E-02 |
| 17644529 | RGD1565247 | 1.21 | 5.E-03 | 6.E-02 | 1.11 | 2.E-04 | 8.E-03 |
| 17820103 | RGD1565310 | -1.44 | 7.E-04 | 2.E-02 | -1.42 | 2.E-05 | 2.E-03 |
| 17816193 | RGD1565317 | 1.1 | 2.E-04 | 9.E-03 | 1.15 | 3.E-05 | 2.E-03 |
| 17641633 | RGD1565682 | -1.65 | 3.E-04 | 1.E-02 | -1.26 | 4.E-03 | 5.E-02 |
| 17870945 | RGD1565685 | -1.31 | 5.E-06 | 1.E-03 | -1.23 | 1.E-04 | 5.E-03 |
| 17644824 | Rhbdf1 | 1.13 | 7.E-03 | 7.E-02 | 1.2 | 6.E-04 | 1.E-02 |
| 17639262 | Rhod | 1.22 | 3.E-03 | 4.E-02 | 1.14 | 5.E-03 | 6.E-02 |
| 17612355 | Riok2 | -1.23 | 1.E-02 | 8.E-02 | -1.29 | 2.E-04 | 6.E-03 |
| 17717968 | Rmi1 | 1.18 | 5.E-03 | 6.E-02 | 1.13 | 5.E-03 | 5.E-02 |
| 17701935 | Rmrp | 1.59 | 3.E-05 | 3.E-03 | 1.39 | 4.E-04 | 1.E-02 |
| 17867715 | Rn5-8s | 1.43 | 9.E-03 | 8.E-02 | 2.01 | 1.E-04 | 6.E-03 |
| 17853806 | Rnf111 | 1.18 | 3.E-03 | 4.E-02 | 1.32 | 3.E-04 | 8.E-03 |
| 17853816 | Rnf111 | 1.17 | 5.E-04 | 2.E-02 | 1.17 | 5.E-03 | 5.E-02 |
| 17855693 | Rnf123 | 1.26 | 6.E-07 | 4.E-04 | 1.19 | 5.E-07 | 4.E-04 |
| 17826580 | Rnf126 | 1.13 | 2.E-04 | 1.E-02 | 1.18 | 1.E-05 | 2.E-03 |
| 17874161 | Rnf128 | -1.19 | 4.E-04 | 1.E-02 | -1.36 | 2.E-05 | 2.E-03 |
| 17738653 | Rnf13 | -1.14 | 5.E-03 | 6.E-02 | -1.29 | 6.E-05 | 4.E-03 |
| 17636230 | rnf141 | -1.24 | 2.E-03 | 4.E-02 | -1.18 | 5.E-04 | 1.E-02 |
| 17798824 | Rnf20 | 1.18 | 9.E-03 | 8.E-02 | 1.1 | 7.E-03 | 6.E-02 |
| 17756041 | Rnf39 | -1.31 | 5.E-03 | 6.E-02 | -1.37 | 2.E-03 | 3.E-02 |
| 17695453 | Rnf4 | -1.12 | 1.E-04 | 7.E-03 | -1.12 | 5.E-03 | 5.E-02 |
| 17620925 | Rnf40 | 1.13 | 5.E-03 | 6.E-02 | 1.12 | 4.E-03 | 4.E-02 |
| 17840500 | Rpap3 | 1.24 | 5.E-04 | 2.E-02 | 1.37 | 5.E-04 | 1.E-02 |
| 17880084 | Rpl10 | 1.19 | 3.E-03 | 5.E-02 | 1.51 | 7.E-05 | 4.E-03 |
| 17811575 | Rpl11 | -1.13 | 1.E-04 | 6.E-03 | -1.16 | 4.E-04 | 1.E-02 |
| 17730854 | Rpl13 | 1.27 | 2.E-04 | 1.E-02 | 1.29 | 6.E-05 | 4.E-03 |
| 17849472 | Rpl13 | 1.2 | 2.E-03 | 4.E-02 | 1.2 | 1.E-04 | 6.E-03 |
| 17649952 | Rpl18 | 2.19 | 2.E-05 | 2.E-03 | 1.81 | 1.E-03 | 2.E-02 |
| 17649960 | Rpl18 | 2.19 | 2.E-05 | 2.E-03 | 1.81 | 1.E-03 | 2.E-02 |
| 17649962 | Rpl18 | 2.19 | 2.E-05 | 2.E-03 | 1.81 | 1.E-03 | 2.E-02 |
| 17668574 | Rpl24 | 1.38 | 1.E-03 | 2.E-02 | 1.17 | 8.E-03 | 7.E-02 |
| 17836741 | Rpl30 | 1.42 | 6.E-04 | 2.E-02 | 3.81 | 5.E-05 | 3.E-03 |
| 17783185 | Rpl30 | 1.6 | 4.E-05 | 3.E-03 | 1.9 | 8.E-08 | 2.E-04 |
| 17645038 | Rpl30 | 1.42 | 6.E-04 | 2.E-02 | 3.81 | 5.E-05 | 3.E-03 |
| 17771871 | Rpl35 | -1.11 | 9.E-03 | 8.E-02 | -1.12 | 6.E-03 | 6.E-02 |
| 17869228 | Rpl36 | -1.17 | 2.E-03 | 3.E-02 | -1.16 | 4.E-03 | 5.E-02 |
| 17632701 | Rps11 | 1.23 | 8.E-03 | 8.E-02 | 1.26 | 5.E-05 | 3.E-03 |
| 17723558 | Rps14 | 1.21 | 2.E-03 | 3.E-02 | 1.14 | 8.E-03 | 7.E-02 |
| 17802105 | Rps14 | 1.28 | 1.E-04 | 7.E-03 | 1.29 | 1.E-05 | 2.E-03 |
| 17614865 | Rps16 | 1.7 | 9.E-05 | 5.E-03 | 2.74 | 1.E-05 | 2.E-03 |
| 17856990 | Rps20 | -1.15 | 2.E-04 | 9.E-03 | -1.19 | 5.E-07 | 4.E-04 |
| 17810126 | Rps27a | 1.16 | 5.E-03 | 6.E-02 | 1.19 | 5.E-04 | 1.E-02 |
| 17653183 | Rptor | 1.15 | 6.E-03 | 7.E-02 | 1.13 | 2.E-03 | 3.E-02 |
| 17748582 | Rrnad1 | 1.19 | 9.E-04 | 2.E-02 | 1.17 | 4.E-03 | 5.E-02 |
| 17754038 | Rrp1b | 1.15 | 3.E-03 | 5.E-02 | 1.2 | 2.E-03 | 3.E-02 |
| 17643505 | Rsl1d1 | -1.17 | 7.E-03 | 7.E-02 | -1.21 | 5.E-03 | 5.E-02 |
| 17756903 | Rxrb | 1.21 | 4.E-05 | 3.E-03 | 1.36 | 4.E-06 | 1.E-03 |
| 17793629 | Rybp | 1.23 | 7.E-04 | 2.E-02 | 1.15 | 4.E-03 | 4.E-02 |
| 17869232 | Safb | 1.23 | 8.E-05 | 5.E-03 | 1.13 | 7.E-03 | 7.E-02 |
| 17631115 | Samd4b | 1.16 | 2.E-05 | 2.E-03 | 1.18 | 9.E-04 | 2.E-02 |
| 17645655 | Sar1b | -1.2 | 2.E-03 | 3.E-02 | -1.33 | 3.E-04 | 1.E-02 |
| 17840024 | Sbf1 | 1.3 | 6.E-06 | 1.E-03 | 1.17 | 6.E-05 | 4.E-03 |
| 17826472 | Sbno2 | 1.35 | 1.E-04 | 6.E-03 | 1.23 | 6.E-03 | 6.E-02 |
| 17747093 | Sclt1 | 1.2 | 8.E-03 | 7.E-02 | 1.27 | 7.E-04 | 1.E-02 |
| 17801708 | Scmh1 | 1.16 | 2.E-05 | 2.E-03 | 1.23 | 1.E-06 | 7.E-04 |
| 17838363 | Scrib | 1.32 | 5.E-07 | 4.E-04 | 1.21 | 1.E-03 | 2.E-02 |
| 17686570 | Sdhc | -1.38 | 5.E-04 | 2.E-02 | -1.25 | 2.E-04 | 8.E-03 |
| 17852281 | Sdhd | -1.17 | 5.E-03 | 6.E-02 | 1.13 | 9.E-03 | 8.E-02 |
| 17718422 | Secisbp2 | 1.12 | 2.E-03 | 3.E-02 | 1.12 | 1.E-04 | 6.E-03 |
| 17840653 | Senp1 | 1.17 | 3.E-03 | 4.E-02 | 1.16 | 1.E-04 | 5.E-03 |
| 17758876 | Serinc1 | -1.15 | 8.E-03 | 7.E-02 | -1.26 | 7.E-04 | 1.E-02 |
| 17743244 | Sep15 | -1.12 | 6.E-03 | 7.E-02 | -1.27 | 2.E-03 | 3.E-02 |
| 17785936 | Setd5 | 1.21 | 2.E-03 | 3.E-02 | 1.11 | 8.E-03 | 7.E-02 |
| 17749457 | Setdb1 | 1.21 | 4.E-03 | 5.E-02 | 1.13 | 1.E-03 | 2.E-02 |
| 17691119 | Sf3a1 | 1.15 | 3.E-03 | 5.E-02 | 1.15 | 8.E-04 | 2.E-02 |
| 17610523 | Sf3b5 | 1.19 | 8.E-03 | 7.E-02 | 1.29 | 2.E-04 | 7.E-03 |
| 17695657 | Sfi1 | 1.22 | 4.E-05 | 4.E-03 | 1.28 | 2.E-04 | 7.E-03 |
| 17802212 | Sfpq | 1.17 | 6.E-03 | 6.E-02 | 1.13 | 1.E-03 | 2.E-02 |
| 17712432 | Sfrp1 | -1.21 | 5.E-03 | 6.E-02 | -1.77 | 7.E-03 | 7.E-02 |
| 17628626 | Sft2d1 | -1.12 | 2.E-03 | 4.E-02 | -1.3 | 2.E-06 | 7.E-04 |
| 17831678 | Shank3 | 1.33 | 2.E-05 | 2.E-03 | 1.18 | 1.E-02 | 8.E-02 |
| 17789613 | Shfm1 | 1.28 | 6.E-03 | 6.E-02 | 1.38 | 6.E-04 | 1.E-02 |
| 17789617 | Shfm1 | 1.37 | 7.E-03 | 7.E-02 | 1.55 | 2.E-03 | 3.E-02 |
| 17626514 | Shoc2 | -1.2 | 8.E-03 | 8.E-02 | -1.18 | 5.E-03 | 5.E-02 |
| 17749616 | similar to H2A histone family, member O /// Hist2h2aa3 | 1.33 | 3.E-03 | 4.E-02 | 1.34 | 2.E-05 | 2.E-03 |
| 17631408 | Sipa1l3 | 1.18 | 3.E-04 | 1.E-02 | 1.19 | 3.E-04 | 9.E-03 |
| 17764675 | Slc12a6 | 1.21 | 3.E-03 | 5.E-02 | -1.14 | 7.E-03 | 6.E-02 |
| 17715316 | Slc22a23 | 1.26 | 1.E-03 | 3.E-02 | -1.53 | 1.E-03 | 2.E-02 |
| 17658152 | Slc25a11 | -1.17 | 2.E-04 | 1.E-02 | -1.2 | 4.E-05 | 3.E-03 |
| 17801572 | Slc2a1 | -1.32 | 1.E-03 | 3.E-02 | -1.26 | 2.E-03 | 3.E-02 |
| 17776606 | Slc30a4 | -1.22 | 7.E-03 | 7.E-02 | -1.34 | 1.E-03 | 2.E-02 |
| 17820353 | Slc30a6 | -1.17 | 4.E-03 | 5.E-02 | -1.23 | 2.E-04 | 7.E-03 |
| 17799179 | Slc31a1 | -1.14 | 3.E-03 | 4.E-02 | -1.44 | 2.E-03 | 3.E-02 |
| 17799173 | Slc31a2 | -1.39 | 2.E-04 | 8.E-03 | -1.62 | 2.E-05 | 2.E-03 |
| 17626306 | Slk | 1.17 | 5.E-03 | 6.E-02 | 1.13 | 3.E-03 | 4.E-02 |
| 17842538 | Smarca4 | 1.25 | 2.E-06 | 8.E-04 | 1.15 | 2.E-05 | 2.E-03 |
| 17754571 | Smarcb1 | 1.13 | 7.E-03 | 7.E-02 | 1.14 | 8.E-04 | 2.E-02 |
| 17806462 | Smu1 | -1.15 | 3.E-05 | 3.E-03 | -1.17 | 7.E-04 | 2.E-02 |
| 17799556 | Snapc3 | 1.22 | 7.E-03 | 7.E-02 | 1.25 | 2.E-03 | 3.E-02 |
| 17781930 | Snd1 | 1.17 | 8.E-05 | 5.E-03 | 1.19 | 2.E-04 | 8.E-03 |
| 17796931 | SNHG6 | 1.7 | 7.E-05 | 5.E-03 | 1.82 | 3.E-05 | 2.E-03 |
| 17802025 | Snip1 | 1.18 | 2.E-04 | 9.E-03 | 1.2 | 4.E-04 | 1.E-02 |
| 17718914 | Snrnp48 | 1.28 | 4.E-03 | 5.E-02 | 1.23 | 3.E-04 | 9.E-03 |
| 17632856 | Snrnp70 | 1.37 | 5.E-05 | 4.E-03 | 1.49 | 2.E-06 | 8.E-04 |
| 17820653 | Snx17 | -1.13 | 2.E-04 | 1.E-02 | -1.12 | 5.E-03 | 5.E-02 |
| 17821903 | Snx6 | -1.14 | 4.E-03 | 5.E-02 | -1.29 | 1.E-04 | 6.E-03 |
| 17664659 | Son | 1.18 | 1.E-05 | 2.E-03 | 1.13 | 6.E-05 | 4.E-03 |
| 17806913 | Spag8 | 1.22 | 3.E-03 | 4.E-02 | 1.37 | 1.E-04 | 6.E-03 |
| 17699403 | Spata13 | 1.34 | 2.E-03 | 3.E-02 | 1.26 | 3.E-04 | 9.E-03 |
| 17738024 | Spata5 | 1.24 | 3.E-03 | 4.E-02 | 1.14 | 3.E-05 | 2.E-03 |
| 17635213 | Spcs2 | -1.19 | 7.E-04 | 2.E-02 | -1.35 | 3.E-05 | 3.E-03 |
| 17707729 | Spcs3 | -1.29 | 1.E-03 | 2.E-02 | -1.34 | 5.E-03 | 5.E-02 |
| 17722920 | Spink5 | -1.19 | 6.E-03 | 7.E-02 | -1.23 | 5.E-03 | 5.E-02 |
| 17703625 | Spryd7 | -1.21 | 5.E-04 | 2.E-02 | -1.17 | 5.E-03 | 5.E-02 |
| 17692187 | Sptbn1 | 1.18 | 2.E-06 | 8.E-04 | -1.16 | 7.E-04 | 2.E-02 |
| 17735510 | Srek1 | 1.23 | 2.E-04 | 8.E-03 | 1.28 | 2.E-04 | 7.E-03 |
| 17644106 | Srrm2 | 1.19 | 8.E-03 | 8.E-02 | 1.14 | 1.E-04 | 6.E-03 |
| 17752199 | Srsf11 | 1.31 | 7.E-06 | 1.E-03 | 1.28 | 5.E-06 | 1.E-03 |
| 17768585 | Srsf6 | 1.17 | 1.E-04 | 6.E-03 | 1.17 | 1.E-03 | 2.E-02 |
| 17715212 | Ssr1 | -1.19 | 2.E-03 | 3.E-02 | -1.23 | 4.E-04 | 1.E-02 |
| 17763529 | Ssrp1 | 1.13 | 4.E-03 | 5.E-02 | 1.14 | 5.E-03 | 5.E-02 |
| 17730206 | St3gal2 | 1.31 | 5.E-04 | 2.E-02 | 1.24 | 9.E-04 | 2.E-02 |
| 17772320 | Stam2 | -1.36 | 8.E-06 | 1.E-03 | -1.23 | 4.E-03 | 4.E-02 |
| 17860585 | Stk16 | -1.2 | 3.E-04 | 1.E-02 | 1.18 | 3.E-03 | 4.E-02 |
| 17771653 | Stom | -1.3 | 2.E-03 | 4.E-02 | -1.34 | 4.E-03 | 5.E-02 |
| 17806744 | Stoml2 | -1.2 | 9.E-05 | 5.E-03 | -1.17 | 3.E-03 | 4.E-02 |
| 17613690 | Strn4 | 1.17 | 8.E-04 | 2.E-02 | 1.19 | 8.E-04 | 2.E-02 |
| 17802785 | Stx12 | -1.14 | 2.E-03 | 3.E-02 | -1.16 | 3.E-03 | 4.E-02 |
| 17769382 | Stx16 | 1.28 | 9.E-04 | 2.E-02 | 1.18 | 5.E-03 | 5.E-02 |
| 17769387 | Stx16 | 1.48 | 2.E-03 | 4.E-02 | 1.29 | 4.E-03 | 5.E-02 |
| 17700024 | Sucla2 | -1.23 | 2.E-04 | 8.E-03 | -1.39 | 1.E-06 | 7.E-04 |
| 17793443 | Suclg2 | -1.22 | 7.E-04 | 2.E-02 | -1.32 | 1.E-04 | 6.E-03 |
| 17833045 | Suox | -1.22 | 5.E-03 | 6.E-02 | -1.42 | 8.E-04 | 2.E-02 |
| 17629590 | Suv420h2 | 1.33 | 5.E-04 | 2.E-02 | 1.29 | 4.E-04 | 1.E-02 |
| 17809882 | Szt2 | 1.33 | 2.E-04 | 8.E-03 | 1.24 | 2.E-05 | 2.E-03 |
| 17624245 | Taf1d | 1.47 | 6.E-04 | 2.E-02 | 1.63 | 7.E-07 | 5.E-04 |
| 17758264 | Taf9 | -1.39 | 5.E-04 | 2.E-02 | -1.39 | 2.E-04 | 6.E-03 |
| 17762332 | Tanc1 | 1.32 | 2.E-05 | 2.E-03 | 1.29 | 8.E-05 | 4.E-03 |
| 17649110 | Taok1 | 1.11 | 4.E-03 | 5.E-02 | 1.12 | 3.E-03 | 4.E-02 |
| 17637288 | Taok2 | 1.36 | 4.E-06 | 1.E-03 | 1.15 | 1.E-03 | 2.E-02 |
| 17690328 | Tapt1 | -1.2 | 7.E-04 | 2.E-02 | -1.37 | 7.E-06 | 1.E-03 |
| 17654312 | Tbc1d24 | 1.23 | 2.E-04 | 9.E-03 | 1.29 | 6.E-03 | 6.E-02 |
| 17862716 | Tbcc | 1.16 | 7.E-03 | 7.E-02 | 1.13 | 7.E-03 | 7.E-02 |
| 17851549 | Tbcel | 1.35 | 2.E-03 | 3.E-02 | 1.25 | 2.E-04 | 6.E-03 |
| 17697484 | Tcea1 | -1.16 | 3.E-05 | 3.E-03 | -1.13 | 2.E-03 | 3.E-02 |
| 17730953 | Tcf25 | 1.11 | 3.E-05 | 3.E-03 | 1.12 | 1.E-04 | 6.E-03 |
| 17719398 | Tdp2 | 1.11 | 4.E-03 | 5.E-02 | 1.21 | 2.E-04 | 7.E-03 |
| 17671773 | Tecpr1 | 1.16 | 6.E-03 | 6.E-02 | 1.22 | 3.E-04 | 8.E-03 |
| 17654672 | Telo2 | 1.13 | 8.E-03 | 8.E-02 | 1.22 | 4.E-04 | 1.E-02 |
| 17652906 | Ten1 | -3.5 | 7.E-04 | 2.E-02 | -1.53 | 1.E-03 | 2.E-02 |
| 17701937 | Tep1 | 1.2 | 9.E-05 | 5.E-03 | 1.17 | 4.E-04 | 1.E-02 |
| 17792617 | Tet3 | 1.33 | 2.E-04 | 9.E-03 | 1.29 | 4.E-05 | 3.E-03 |
| 17630489 | Tex101 | -1.18 | 6.E-03 | 7.E-02 | -1.19 | 1.E-03 | 2.E-02 |
| 17628324 | Tfb1m | -1.23 | 1.E-03 | 2.E-02 | -1.23 | 4.E-03 | 4.E-02 |
| 17705188 | Tgds | 1.13 | 5.E-03 | 6.E-02 | 1.17 | 5.E-04 | 1.E-02 |
| 17717447 | Thnsl1 | 1.35 | 8.E-04 | 2.E-02 | 1.19 | 9.E-03 | 8.E-02 |
| 17810522 | Thrap3 | 1.18 | 1.E-03 | 3.E-02 | 1.16 | 3.E-03 | 4.E-02 |
| 17810525 | Thrap3 | -1.17 | 2.E-03 | 3.E-02 | -1.19 | 5.E-04 | 1.E-02 |
| 17856799 | Thyn1 | 1.23 | 4.E-03 | 5.E-02 | 1.24 | 3.E-03 | 4.E-02 |
| 17785159 | Tia1 | 1.24 | 1.E-03 | 2.E-02 | 1.26 | 6.E-05 | 4.E-03 |
| 17637595 | Tial1 | 1.18 | 5.E-04 | 2.E-02 | 1.16 | 8.E-04 | 2.E-02 |
| 17826210 | Timm13 | 1.17 | 5.E-03 | 6.E-02 | 1.24 | 1.E-03 | 2.E-02 |
| 17665847 | Timmdc1 | -1.25 | 2.E-03 | 3.E-02 | -1.19 | 7.E-03 | 7.E-02 |
| 17870139 | Timmdc1 | -1.24 | 1.E-03 | 3.E-02 | -1.22 | 2.E-04 | 7.E-03 |
| 17857508 | Tjap1 | 1.12 | 6.E-03 | 7.E-02 | 1.25 | 2.E-03 | 3.E-02 |
| 17808766 | Tm2d1 | -1.29 | 1.E-03 | 2.E-02 | -1.33 | 1.E-04 | 5.E-03 |
| 17696114 | Tmed4 | -1.3 | 1.E-04 | 7.E-03 | -1.27 | 1.E-04 | 6.E-03 |
| 17687951 | Tmed5 | -1.2 | 8.E-03 | 7.E-02 | -1.2 | 5.E-03 | 5.E-02 |
| 17718103 | Tmed9 | 1.19 | 1.E-03 | 3.E-02 | 1.23 | 7.E-04 | 1.E-02 |
| 17789755 | Tmem106b | -1.25 | 6.E-03 | 7.E-02 | -1.16 | 1.E-02 | 8.E-02 |
| 17640264 | Tmem179b | -1.21 | 1.E-04 | 6.E-03 | -1.19 | 4.E-03 | 5.E-02 |
| 17815243 | Tmem18 | -1.12 | 2.E-03 | 3.E-02 | -1.16 | 3.E-03 | 4.E-02 |
| 17732130 | Tmem188 | -1.14 | 2.E-03 | 3.E-02 | -1.24 | 3.E-03 | 3.E-02 |
| 17658943 | Tmem199 | -1.15 | 7.E-03 | 7.E-02 | -1.11 | 7.E-03 | 7.E-02 |
| 17759845 | Tmem203 | -1.24 | 4.E-04 | 1.E-02 | -1.17 | 4.E-03 | 5.E-02 |
| 17729370 | Tmem208 | 1.19 | 2.E-03 | 3.E-02 | 1.19 | 4.E-05 | 3.E-03 |
| 17640498 | Tmem216 | -1.26 | 7.E-05 | 5.E-03 | -1.14 | 9.E-03 | 8.E-02 |
| 17843141 | Tmem218 | -1.39 | 2.E-03 | 3.E-02 | -1.31 | 2.E-05 | 2.E-03 |
| 17854257 | Tmem30a | -1.12 | 3.E-03 | 4.E-02 | -1.17 | 3.E-03 | 4.E-02 |
| 17801432 | Tmem53 | -1.45 | 8.E-07 | 4.E-04 | -1.28 | 4.E-03 | 4.E-02 |
| 17775340 | Tmem85 | -1.2 | 1.E-03 | 3.E-02 | -1.26 | 9.E-05 | 4.E-03 |
| 17658444 | Tmem93 | -1.28 | 1.E-03 | 3.E-02 | -1.32 | 5.E-04 | 1.E-02 |
| 17773987 | Tmx2 | -1.31 | 3.E-05 | 3.E-03 | -1.39 | 1.E-04 | 5.E-03 |
| 17708250 | Tnks | 1.16 | 1.E-04 | 7.E-03 | 1.13 | 2.E-03 | 3.E-02 |
| 17732381 | Tnpo2 | 1.15 | 6.E-03 | 6.E-02 | 1.16 | 1.E-04 | 6.E-03 |
| 17620441 | Tnrc6a | 1.22 | 4.E-03 | 5.E-02 | 1.24 | 2.E-05 | 2.E-03 |
| 17696224 | Tns3 | 1.22 | 1.E-04 | 7.E-03 | 1.15 | 3.E-03 | 3.E-02 |
| 17656795 | Tom1l2 | 1.12 | 4.E-04 | 1.E-02 | 1.18 | 1.E-05 | 2.E-03 |
| 17638921 | Tpcn2 | 1.31 | 2.E-03 | 4.E-02 | 1.24 | 3.E-03 | 4.E-02 |
| 17700148 | Tpt1 | 1.29 | 1.E-05 | 2.E-03 | 1.16 | 4.E-03 | 4.E-02 |
| 17791245 | Tra2a | 1.14 | 8.E-03 | 7.E-02 | 1.17 | 4.E-04 | 1.E-02 |
| 17831571 | Trabd | 1.13 | 3.E-03 | 4.E-02 | 1.13 | 5.E-03 | 5.E-02 |
| 17770315 | Traf2 | 1.17 | 8.E-03 | 8.E-02 | 1.12 | 3.E-03 | 4.E-02 |
| 17654433 | Traf7 | 1.21 | 8.E-04 | 2.E-02 | 1.15 | 8.E-03 | 7.E-02 |
| 17848969 | Trak1 | 1.22 | 2.E-04 | 1.E-02 | 1.23 | 1.E-05 | 2.E-03 |
| 17655761 | Trim41 | 1.15 | 8.E-06 | 1.E-03 | 1.16 | 7.E-06 | 1.E-03 |
| 17670641 | Trmt2a | 1.21 | 3.E-05 | 3.E-03 | 1.33 | 4.E-04 | 1.E-02 |
| 17675838 | Trrap | 1.28 | 6.E-05 | 4.E-03 | 1.19 | 2.E-06 | 8.E-04 |
| 17789921 | Tspan12 | -1.26 | 6.E-03 | 6.E-02 | -1.32 | 2.E-04 | 7.E-03 |
| 17836379 | Tspan31 | -1.34 | 4.E-04 | 1.E-02 | -1.5 | 4.E-05 | 3.E-03 |
| 17878725 | Tspan6 | -1.31 | 1.E-05 | 2.E-03 | -1.4 | 1.E-03 | 2.E-02 |
| 17622529 | Tssc4 | 1.14 | 1.E-03 | 3.E-02 | 1.14 | 7.E-03 | 7.E-02 |
| 17839876 | Tubgcp6 | 1.18 | 1.E-03 | 3.E-02 | 1.18 | 1.E-03 | 2.E-02 |
| 17611692 | Tulp4 | 1.29 | 4.E-04 | 1.E-02 | 1.15 | 2.E-04 | 7.E-03 |
| 17800790 | Txndc12 | -1.31 | 7.E-04 | 2.E-02 | -1.29 | 2.E-03 | 3.E-02 |
| 17850265 | Tyk2 | 1.23 | 3.E-04 | 1.E-02 | 1.2 | 1.E-03 | 2.E-02 |
| 17875770 | Uba1 | 1.42 | 7.E-05 | 5.E-03 | 1.34 | 6.E-05 | 4.E-03 |
| 17793522 | Uba3 | -1.16 | 6.E-04 | 2.E-02 | -1.13 | 2.E-03 | 3.E-02 |
| 17801569 | Ubb | 1.24 | 2.E-05 | 2.E-03 | 1.12 | 4.E-04 | 1.E-02 |
| 17802877 | Ubb | -1.12 | 2.E-04 | 1.E-02 | 1.14 | 5.E-03 | 5.E-02 |
| 17742869 | Ube2d3 /// Ube2d2 | -1.1 | 2.E-04 | 1.E-02 | -1.12 | 8.E-04 | 2.E-02 |
| 17742870 | Ube2d3 /// Ube2d2 | -1.13 | 3.E-04 | 1.E-02 | -1.24 | 4.E-04 | 1.E-02 |
| 17861579 | Ube2f | -1.18 | 7.E-03 | 7.E-02 | -1.17 | 7.E-04 | 2.E-02 |
| 17648071 | Ube2g1 | -1.35 | 7.E-06 | 1.E-03 | -1.29 | 8.E-04 | 2.E-02 |
| 17694447 | Ube2k | -1.25 | 4.E-05 | 3.E-03 | -1.17 | 2.E-03 | 3.E-02 |
| 17803594 | Ubr4 | 1.31 | 3.E-08 | 6.E-05 | 1.13 | 1.E-03 | 2.E-02 |
| 17708329 | Ubxn8 | -1.62 | 2.E-04 | 9.E-03 | -1.71 | 8.E-05 | 4.E-03 |
| 17780215 | Uckl1 /// Zfp512b | 1.24 | 7.E-03 | 7.E-02 | 1.22 | 2.E-04 | 7.E-03 |
| 17634269 | Unc45a | 1.15 | 3.E-04 | 1.E-02 | 1.16 | 5.E-04 | 1.E-02 |
| 17656084 | Uqcrq | 1.18 | 3.E-03 | 4.E-02 | 1.25 | 4.E-05 | 3.E-03 |
| 17681931 | Usf1 | 1.18 | 2.E-03 | 4.E-02 | 1.21 | 8.E-04 | 2.E-02 |
| 17631771 | Usf2 | 1.14 | 3.E-03 | 4.E-02 | 1.19 | 3.E-05 | 2.E-03 |
| 17713409 | Usp19 | 1.18 | 3.E-06 | 9.E-04 | 1.15 | 2.E-05 | 2.E-03 |
| 17743517 | Usp33 | 1.13 | 2.E-03 | 3.E-02 | 1.15 | 2.E-04 | 7.E-03 |
| 17666520 | Uts2b | -1.16 | 3.E-03 | 5.E-02 | -1.19 | 6.E-04 | 1.E-02 |
| 17875709 | Uxt | -1.21 | 8.E-06 | 1.E-03 | -1.18 | 1.E-03 | 2.E-02 |
| 17676488 | Vamp7 | -1.17 | 3.E-04 | 1.E-02 | -1.13 | 1.E-05 | 2.E-03 |
| 17792277 | Vamp8 | 1.29 | 2.E-04 | 9.E-03 | 1.33 | 1.E-05 | 2.E-03 |
| 17752661 | Vars2 | 1.34 | 2.E-05 | 2.E-03 | 1.33 | 3.E-04 | 1.E-02 |
| 17722610 | vault RNA | 2.93 | 2.E-09 | 1.E-05 | 2.57 | 6.E-06 | 1.E-03 |
| 17806696 | Vcp | 1.33 | 7.E-04 | 2.E-02 | 1.37 | 1.E-03 | 2.E-02 |
| 17806702 | Vcp | 1.15 | 5.E-03 | 6.E-02 | 1.13 | 9.E-03 | 7.E-02 |
| 17806710 | Vcp | -1.19 | 3.E-03 | 4.E-02 | -1.25 | 9.E-03 | 8.E-02 |
| 17812321 | Vps13d | 1.28 | 1.E-05 | 2.E-03 | 1.14 | 3.E-05 | 3.E-03 |
| 17673745 | Vps37b | 1.22 | 3.E-03 | 4.E-02 | 1.41 | 2.E-05 | 2.E-03 |
| 17719725 | Vps41 | -1.13 | 3.E-04 | 1.E-02 | -1.15 | 5.E-07 | 4.E-04 |
| 17756917 | Vps52 | 1.1 | 7.E-03 | 7.E-02 | 1.18 | 8.E-05 | 4.E-03 |
| 17871088 | Wdr13 | 1.17 | 9.E-04 | 2.E-02 | 1.2 | 2.E-06 | 8.E-04 |
| 17655423 | Wwc1 | 1.24 | 4.E-04 | 1.E-02 | 1.16 | 2.E-03 | 3.E-02 |
| 17880461 | XM_001066874 | -1.15 | 3.E-03 | 4.E-02 | -1.2 | 3.E-03 | 4.E-02 |
| 17793125 | XM_002729398 | 1.21 | 6.E-04 | 2.E-02 | 1.15 | 4.E-03 | 5.E-02 |
| 17700936 | XM_003751432 /// Myst4 | 1.23 | 5.E-04 | 2.E-02 | 1.17 | 2.E-05 | 2.E-03 |
| 17799940 | Y1 | 1.43 | 6.E-03 | 6.E-02 | 2.4 | 1.E-05 | 2.E-03 |
| 17791155 | Y1 | 2.33 | 2.E-07 | 2.E-04 | 2.43 | 4.E-07 | 4.E-04 |
| 17667325 | Yars2 | -1.37 | 3.E-04 | 1.E-02 | -1.23 | 5.E-03 | 5.E-02 |
| 17636536 | Ybx1 | 1.25 | 1.E-03 | 3.E-02 | 1.41 | 2.E-07 | 3.E-04 |
| 17810097 | Ybx1 | 1.12 | 1.E-03 | 2.E-02 | 1.11 | 6.E-04 | 1.E-02 |
| 17656249 | Ybx1 /// Ybx1-ps3 | 1.73 | 1.E-04 | 7.E-03 | 1.17 | 2.E-04 | 7.E-03 |
| 17817443 | Ylpm1 | 1.17 | 9.E-03 | 8.E-02 | 1.16 | 5.E-04 | 1.E-02 |
| 17801970 | Yrdc | 1.22 | 1.E-04 | 7.E-03 | 1.19 | 1.E-03 | 2.E-02 |
| 17780053 | Ythdf1 | 1.22 | 9.E-04 | 2.E-02 | 1.25 | 4.E-03 | 4.E-02 |
| 17647526 | Zbtb4 | 1.16 | 2.E-03 | 4.E-02 | 1.17 | 3.E-04 | 9.E-03 |
| 17811662 | Zbtb40 | 1.23 | 4.E-04 | 1.E-02 | 1.19 | 2.E-03 | 3.E-02 |
| 17842857 | Zbtb44 | 1.16 | 2.E-03 | 4.E-02 | 1.11 | 6.E-03 | 6.E-02 |
| 17833513 | Zbtb7a | 1.25 | 4.E-05 | 3.E-03 | 1.14 | 3.E-04 | 9.E-03 |
| 17684242 | Zc3h11a | 1.22 | 7.E-04 | 2.E-02 | 1.12 | 6.E-03 | 6.E-02 |
| 17700110 | Zc3h13 | 1.11 | 4.E-03 | 5.E-02 | 1.12 | 3.E-04 | 9.E-03 |
| 17830958 | Zc3h7b | 1.28 | 4.E-04 | 1.E-02 | 1.16 | 4.E-03 | 5.E-02 |
| 17734045 | Zcchc14 | 1.25 | 2.E-04 | 9.E-03 | 1.12 | 1.E-02 | 8.E-02 |
| 17679376 | Zcchc2 | 1.21 | 8.E-03 | 7.E-02 | 1.12 | 1.E-03 | 2.E-02 |
| 17771059 | Zdhhc12 | -1.21 | 7.E-03 | 7.E-02 | -1.18 | 2.E-03 | 3.E-02 |
| 17808317 | Zdhhc21 | -1.18 | 8.E-04 | 2.E-02 | -1.18 | 1.E-03 | 2.E-02 |
| 17771066 | Zer1 | 1.27 | 9.E-05 | 5.E-03 | 1.15 | 2.E-03 | 3.E-02 |
| 17860557 | Zfand2b | 1.11 | 9.E-03 | 8.E-02 | 1.2 | 3.E-04 | 8.E-03 |
| 17634709 | Zfand6 | 1.16 | 1.E-03 | 3.E-02 | 1.15 | 1.E-05 | 2.E-03 |
| 17769169 | Zfas1 | 1.89 | 7.E-06 | 1.E-03 | 1.97 | 9.E-07 | 6.E-04 |
| 17769171 | Zfas1 | 2.61 | 6.E-08 | 7.E-05 | 2.09 | 1.E-05 | 2.E-03 |
| 17729875 | Zfhx3 | 1.32 | 8.E-04 | 2.E-02 | 1.24 | 4.E-04 | 1.E-02 |
| 17865490 | Zfp142 | 1.38 | 4.E-04 | 1.E-02 | 1.39 | 1.E-06 | 7.E-04 |
| 17715845 | Zfp192 | 1.29 | 2.E-04 | 8.E-03 | 1.41 | 5.E-05 | 3.E-03 |
| 17702091 | Zfp219 | 1.24 | 5.E-05 | 4.E-03 | 1.3 | 4.E-05 | 3.E-03 |
| 17783195 | Zfp282 | 1.13 | 7.E-04 | 2.E-02 | 1.21 | 2.E-04 | 7.E-03 |
| 17862740 | Zfp318 | 1.24 | 2.E-04 | 9.E-03 | 1.14 | 3.E-03 | 4.E-02 |
| 17629861 | Zfp324 | 1.17 | 3.E-03 | 4.E-02 | 1.21 | 5.E-03 | 5.E-02 |
| 17779303 | Zfp335 | 1.25 | 2.E-04 | 9.E-03 | 1.24 | 2.E-03 | 3.E-02 |
| 17850063 | Zfp426 | 1.12 | 4.E-03 | 5.E-02 | 1.16 | 3.E-03 | 4.E-02 |
| 17671953 | Zfp469 | 1.3 | 2.E-03 | 4.E-02 | 1.2 | 1.E-03 | 2.E-02 |
| 17724586 | Zfp516 | 1.2 | 2.E-03 | 3.E-02 | 1.17 | 8.E-03 | 7.E-02 |
| 17617834 | Zfp592 | 1.42 | 1.E-03 | 2.E-02 | 1.46 | 9.E-05 | 4.E-03 |
| 17644315 | Zfp598 | 1.18 | 7.E-04 | 2.E-02 | 1.12 | 4.E-03 | 5.E-02 |
| 17868745 | Zfp609 | 1.35 | 2.E-04 | 9.E-03 | 1.23 | 3.E-06 | 9.E-04 |
| 17637477 | Zfp629 | 1.15 | 2.E-03 | 3.E-02 | 1.27 | 5.E-04 | 1.E-02 |
| 17637453 | Zfp688 | -1.41 | 1.E-04 | 7.E-03 | -1.21 | 6.E-03 | 6.E-02 |
| 17646126 | Zfp692 | 1.17 | 9.E-03 | 8.E-02 | 1.28 | 7.E-06 | 1.E-03 |
| 17791177 | Zfp777 | 1.14 | 9.E-03 | 8.E-02 | 1.24 | 5.E-03 | 6.E-02 |
| 17822859 | Zfyve26 | 1.21 | 2.E-03 | 3.E-02 | 1.13 | 2.E-03 | 3.E-02 |
| 17722603 | Zmat2 | -1.13 | 2.E-03 | 3.E-02 | -1.14 | 3.E-03 | 4.E-02 |
| 17847455 | Zmynd10 | 1.19 | 4.E-03 | 5.E-02 | 1.45 | 2.E-04 | 7.E-03 |
| 17648107 | Zzef1 | 1.26 | 2.E-05 | 2.E-03 | 1.18 | 1.E-05 | 2.E-03 |
| 17600287 | --- | 1.27 | 5.E-03 | 6.E-02 | 1.36 | 5.E-03 | 5.E-02 |
| 17600475 | --- | 1.57 | 2.E-04 | 1.E-02 | 1.65 | 3.E-03 | 4.E-02 |
| 17600481 | --- | 1.47 | 9.E-04 | 2.E-02 | 1.47 | 9.E-03 | 8.E-02 |
| 17600843 | --- | 1.5 | 3.E-03 | 5.E-02 | 1.65 | 5.E-04 | 1.E-02 |
| 17601095 | --- | -1.45 | 9.E-04 | 2.E-02 | -1.67 | 4.E-05 | 3.E-03 |
| 17601429 | --- | -1.45 | 9.E-04 | 2.E-02 | -1.67 | 4.E-05 | 3.E-03 |
| 17602263 | --- | 1.57 | 6.E-03 | 6.E-02 | 1.39 | 2.E-03 | 3.E-02 |
| 17602433 | --- | 1.4 | 3.E-03 | 4.E-02 | 1.45 | 2.E-04 | 7.E-03 |
| 17602445 | --- | 1.55 | 5.E-04 | 2.E-02 | 1.23 | 7.E-03 | 6.E-02 |
| 17602637 | --- | 2.17 | 3.E-06 | 8.E-04 | 1.59 | 6.E-05 | 4.E-03 |
| 17603029 | --- | 1.65 | 6.E-04 | 2.E-02 | 1.73 | 8.E-03 | 7.E-02 |
| 17603109 | --- | 1.51 | 5.E-04 | 2.E-02 | 1.27 | 1.E-02 | 8.E-02 |
| 17603215 | --- | 1.58 | 5.E-03 | 6.E-02 | 1.74 | 9.E-03 | 8.E-02 |
| 17603265 | --- | 1.44 | 8.E-03 | 8.E-02 | 1.3 | 7.E-03 | 7.E-02 |
| 17603363 | --- | 1.52 | 1.E-05 | 2.E-03 | 1.45 | 2.E-04 | 7.E-03 |
| 17603369 | --- | 1.63 | 2.E-04 | 8.E-03 | 1.48 | 5.E-04 | 1.E-02 |
| 17603377 | --- | 1.46 | 6.E-04 | 2.E-02 | 1.44 | 4.E-03 | 4.E-02 |
| 17603385 | --- | 1.52 | 3.E-06 | 8.E-04 | 1.36 | 8.E-03 | 7.E-02 |
| 17603433 | --- | 1.53 | 2.E-04 | 9.E-03 | 1.55 | 4.E-03 | 5.E-02 |
| 17603445 | --- | 1.88 | 6.E-07 | 4.E-04 | 1.39 | 5.E-03 | 5.E-02 |
| 17603449 | --- | 2.15 | 8.E-05 | 5.E-03 | 1.52 | 9.E-03 | 8.E-02 |
| 17603463 | --- | 1.71 | 2.E-08 | 4.E-05 | 1.33 | 2.E-03 | 3.E-02 |
| 17603489 | --- | 1.4 | 7.E-04 | 2.E-02 | 1.4 | 2.E-04 | 7.E-03 |
| 17603689 | --- | 1.5 | 6.E-03 | 7.E-02 | 1.84 | 3.E-03 | 4.E-02 |
| 17603979 | --- | 1.56 | 2.E-03 | 3.E-02 | 1.49 | 6.E-03 | 6.E-02 |
| 17604357 | --- | 1.64 | 1.E-03 | 3.E-02 | 1.56 | 4.E-03 | 5.E-02 |
| 17604363 | --- | 1.49 | 4.E-04 | 1.E-02 | 1.43 | 7.E-03 | 7.E-02 |
| 17604625 | --- | 1.43 | 6.E-04 | 2.E-02 | 1.3 | 3.E-03 | 4.E-02 |
| 17604681 | --- | 1.81 | 2.E-03 | 4.E-02 | -1.67 | 8.E-03 | 7.E-02 |
| 17604919 | --- | 2.01 | 2.E-03 | 3.E-02 | 1.7 | 4.E-03 | 5.E-02 |
| 17604921 | --- | 1.46 | 2.E-03 | 4.E-02 | 1.75 | 1.E-05 | 2.E-03 |
| 17604931 | --- | 1.49 | 8.E-03 | 8.E-02 | 1.72 | 7.E-03 | 7.E-02 |
| 17605309 | --- | 1.36 | 3.E-03 | 4.E-02 | 1.56 | 6.E-03 | 6.E-02 |
| 17605341 | --- | 1.4 | 8.E-03 | 8.E-02 | 1.56 | 5.E-04 | 1.E-02 |
| 17605817 | --- | 1.61 | 4.E-04 | 1.E-02 | 1.31 | 1.E-03 | 2.E-02 |
| 17605887 | --- | 1.45 | 6.E-03 | 7.E-02 | 1.26 | 7.E-03 | 7.E-02 |
| 17606149 | --- | 1.46 | 2.E-03 | 3.E-02 | 1.43 | 3.E-04 | 9.E-03 |
| 17606233 | --- | 1.48 | 7.E-03 | 7.E-02 | 1.6 | 6.E-03 | 6.E-02 |
| 17606259 | --- | 1.36 | 9.E-03 | 8.E-02 | 1.45 | 2.E-03 | 3.E-02 |
| 17606621 | --- | 1.77 | 3.E-05 | 3.E-03 | 1.67 | 3.E-04 | 1.E-02 |
| 17606629 | --- | 1.4 | 8.E-03 | 8.E-02 | 1.52 | 2.E-03 | 3.E-02 |
| 17606757 | --- | -1.27 | 9.E-03 | 8.E-02 | -1.49 | 3.E-03 | 4.E-02 |
| 17607265 | --- | 1.77 | 3.E-05 | 3.E-03 | 1.67 | 3.E-04 | 1.E-02 |
| 17607273 | --- | 1.4 | 8.E-03 | 8.E-02 | 1.52 | 2.E-03 | 3.E-02 |
| 17607427 | --- | 1.64 | 6.E-03 | 6.E-02 | 1.87 | 4.E-03 | 4.E-02 |
| 17607573 | --- | 1.52 | 8.E-03 | 8.E-02 | 1.69 | 3.E-04 | 9.E-03 |
| 17607655 | --- | 1.39 | 6.E-03 | 6.E-02 | 1.62 | 2.E-05 | 2.E-03 |
| 17607663 | --- | 1.63 | 1.E-03 | 3.E-02 | 1.61 | 1.E-03 | 2.E-02 |
| 17607677 | --- | 1.9 | 1.E-03 | 3.E-02 | 1.51 | 4.E-03 | 4.E-02 |
| 17608857 | --- | 1.37 | 8.E-03 | 7.E-02 | 1.52 | 6.E-04 | 1.E-02 |
| 17608991 | --- | 1.52 | 8.E-03 | 8.E-02 | 1.69 | 3.E-04 | 9.E-03 |
| 17609073 | --- | 1.39 | 6.E-03 | 6.E-02 | 1.62 | 2.E-05 | 2.E-03 |
| 17609081 | --- | 1.63 | 1.E-03 | 3.E-02 | 1.61 | 1.E-03 | 2.E-02 |
| 17609095 | --- | 1.9 | 1.E-03 | 3.E-02 | 1.51 | 4.E-03 | 4.E-02 |
| 17609343 | --- | 1.3 | 1.E-03 | 3.E-02 | 1.34 | 2.E-03 | 3.E-02 |
| 17609349 | --- | 1.47 | 5.E-04 | 2.E-02 | 1.35 | 9.E-03 | 8.E-02 |
| 17609445 | --- | 1.52 | 6.E-03 | 6.E-02 | 1.53 | 8.E-03 | 7.E-02 |
| 17609517 | --- | 1.93 | 8.E-04 | 2.E-02 | 1.65 | 7.E-03 | 7.E-02 |
| 17609521 | --- | 1.62 | 8.E-04 | 2.E-02 | 1.29 | 9.E-03 | 8.E-02 |
| 17609571 | --- | 1.36 | 3.E-03 | 4.E-02 | 1.46 | 2.E-03 | 3.E-02 |
| 17609575 | --- | 1.44 | 2.E-03 | 4.E-02 | 1.51 | 2.E-04 | 7.E-03 |
| 17609591 | --- | 1.59 | 7.E-03 | 7.E-02 | 1.73 | 2.E-03 | 3.E-02 |
| 17609607 | --- | 1.57 | 1.E-03 | 3.E-02 | 1.4 | 2.E-03 | 3.E-02 |
| 17609729 | --- | 1.64 | 6.E-05 | 4.E-03 | 1.53 | 7.E-05 | 4.E-03 |
| 17610031 | --- | 1.63 | 2.E-03 | 3.E-02 | 1.78 | 3.E-04 | 1.E-02 |
| 17627733 | --- | -1.28 | 9.E-03 | 8.E-02 | -1.26 | 3.E-03 | 4.E-02 |
| 17632292 | --- | -1.33 | 2.E-03 | 4.E-02 | -1.27 | 2.E-03 | 3.E-02 |
| 17645302 | --- | 1.3 | 1.E-04 | 7.E-03 | 1.29 | 2.E-04 | 6.E-03 |
| 17671398 | --- | 1.27 | 7.E-04 | 2.E-02 | 1.87 | 8.E-04 | 2.E-02 |
| 17793044 | --- | 1.35 | 1.E-05 | 2.E-03 | 1.77 | 2.E-04 | 7.E-03 |
| 17809820 | --- | 1.45 | 4.E-04 | 1.E-02 | 1.29 | 3.E-03 | 4.E-02 |
| 17812447 | --- | 1.36 | 2.E-03 | 4.E-02 | 1.22 | 5.E-03 | 6.E-02 |
| 17839274 | --- | 1.35 | 2.E-03 | 3.E-02 | 1.96 | 1.E-04 | 6.E-03 |
| 17850777 | --- | -1.2 | 6.E-05 | 4.E-03 | -1.29 | 5.E-07 | 4.E-04 |
| 17868954 | --- | 1.39 | 9.E-03 | 8.E-02 | 1.43 | 6.E-03 | 6.E-02 |
| 17869072 | --- | -1.16 | 6.E-03 | 6.E-02 | -1.2 | 1.E-03 | 2.E-02 |
| 17881275 | --- | -1.15 | 4.E-03 | 5.E-02 | -1.13 | 3.E-03 | 4.E-02 |
| 17883223 | --- | 1.85 | 1.E-06 | 5.E-04 | 1.93 | 1.E-04 | 6.E-03 |
| 17883252 | --- | -1.2 | 5.E-04 | 2.E-02 | -1.47 | 3.E-04 | 9.E-03 |
