# Supplementary material for: Transcriptome profile analysis reflects rat liver and kidney damage following chronic ultra-low dose Roundup exposure
Source: Environ Health. 2015 Aug 25;14:70. doi: 10.1186/s12940-015-0056-1 (PMC4549093; doi:10.1186/s12940-015-0056-1)
Supplement: Additional file 6: — Microarray data is confirmed by RT-qPCR analysis. A total of 18 genes were randomly selected among the 1319 transcript clusters whose expression was commonly up- or downregulated in liver and kidneys as shown by microarray analysis, were chosen for validation by RT-qPCR. Data from the microarray analysis is depicted in grey bars and that from the RT-qPCR in black bars. The RT-qPCR was performed by TaqMan assay in quadruplicate and standardised against 4 reference genes (Gapdh, Hprt1, Actb and Pes1). Two-tailed Student’s t-test was performed comparing the Roundup-treated group to their respective controls (* p <0.05, ** p <0.01, ***p <0.001). The overall pattern of the RT-qPCR confirmed the microarray analysis results. (DOCX 38 kb) [file 12940_2015_56_MOESM6_ESM.docx]

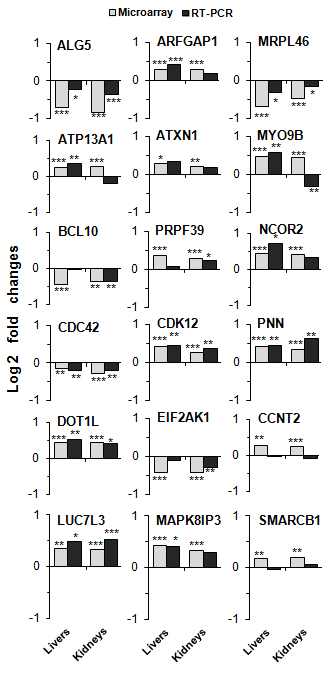


**Additional file 6. Microarray data is confirmed by by RT-qPCR analysis.** A total of 18 genes were randomly selected among the 1319 whose expression was commonly up- or downregulated in liver and kidneys as shown by microarray analysis, were chosen for validation by RT-qPCR. Data from the microarray analysis is depicted in grey bars and that from the RT-qPCR in black bars. The RT-qPCR was performed by TaqMan assay in quadruplicate and standardised against 4 reference genes (*Gapdh*, , *Hprt1*, *Actb* and *Pes1*). Two-tailed Student’s t-test were performed comparing the Roundup-treated group to their respective controls (* p<0.05, ** p<0.01, ***p<0.001). The overall pattern of the RT-qPCR confirmed the microarray analysis results.
