# Supplementary figures and images for: Transcriptome profile analysis reflects rat liver and kidney damage following chronic ultra-low dose Roundup exposure
Source: Environ Health. 2015 Aug 25;14:70. doi: 10.1186/s12940-015-0056-1 (PMC4549093; doi:10.1186/s12940-015-0056-1)

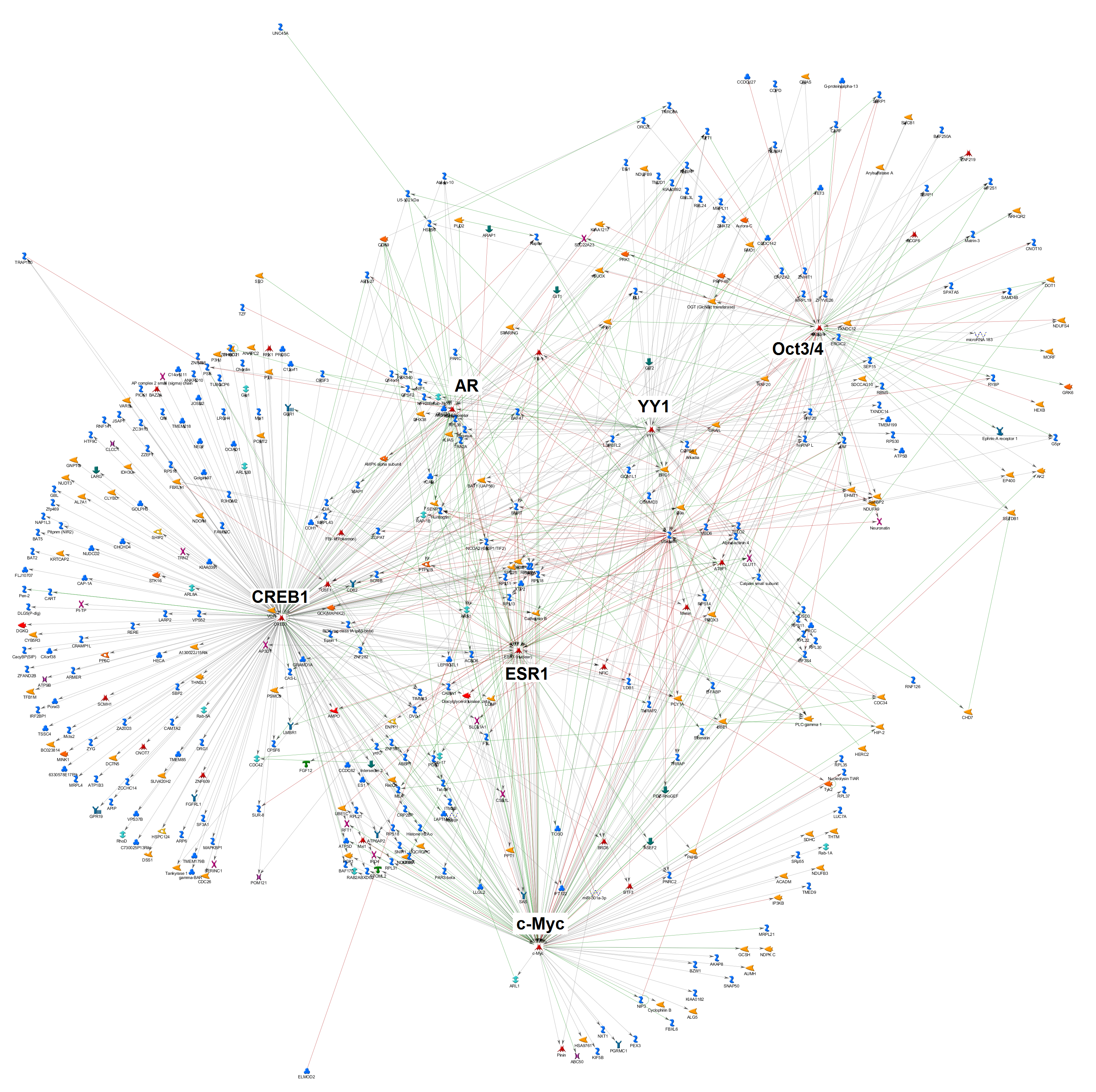

Supplement: Additional file 7: — Transcription factor networks associated with transcriptional alteration of genes commonly disturbed in liver and kidneys. The list of transcript clusters commonly disturbed in liver and kidneys is used for generation of networks using Transcription Regulation algorithm with default settings with MetaCore. (TIFF 4891 kb) [file 12940_2015_56_MOESM7_ESM.tiff]
